# Effects of the World Health Organization Safe Childbirth Checklist on Quality of Care and Birth Outcomes in Aceh, Indonesia: A Cluster-Randomized Clinical Trial

**DOI:** 10.1001/jamanetworkopen.2021.37168

**Published:** 2021-12-03

**Authors:** Lennart Christian Kaplan, Ichsan Ichsan, Farah Diba, Marthoenis Marthoenis, Muhsin Muhsin, Samadi Samadi, Katharina Richert, Suryane Sulistiana Susanti, Hizir Sofyan, Sebastian Vollmer

**Affiliations:** 1 Department of Economics, University of Göttingen, Göttingen, Germany; 2 German Development Institute, Bonn, Germany; 3 Syiah Kuala University, Banda Aceh, Indonesia; 4 Department of Economics, University of Mannheim, Mannheim, Germany; 5 Centre for Modern Indian Studies, University of Göttingen, Göttingen, Germany

## Abstract

**Importance:**

To address major causes of perinatal and maternal mortality, the World Health Organization developed the Safe Childbirth Checklist (SCC), which to our knowledge has been rigorously evaluated only in combination with high-intensity coaching.

**Objective:**

To evaluate the effect of the SCC with medium-intensity coaching on health care workers’ performance of essential birth practices.

**Design, Setting, and Participants:**

This cluster randomized clinical trial without blinding included 32 hospitals and community health centers in the province of Aceh, Indonesia (a medium-resource setting) that met the criterion of providing at least basic emergency obstetric and newborn care. Baseline data were collected from August to October 2016, and outcomes were measured from March to April 2017. Data were analyzed from January 2020 to October 2021.

**Interventions:**

After applying an optimization method, facilities were randomly assigned to the treatment or control group (16 facilities each). The SCC with 11 coaching visits was implemented during a 6-month period.

**Main Outcomes and Measures:**

For the primary outcome, clinical observers documented whether 36 essential birth practices were applied at treatment and control facilities at 1 or more of 4 pause points during the birthing process (admission to the hospital, just before pushing or cesarean delivery, soon after birth, and before hospital discharge). Probability models for binary outcome measures were estimated using ordinary least-squares regressions, complemented by Firth logit and complier average causal effect estimations.

**Results:**

Among the 32 facilities that participated in the trial, a significant increase of up to 41 percentage points was observed in the application of 5 of 36 essential birth practices in the 16 treatment facilities compared with the 16 control facilities, including communication of danger signs at admission (treatment: 136 of 155 births [88%]; control: 79 of 107 births [74%]), measurement of neonatal temperature (treatment: 9 of 31 births [29%]; control: 1 of 20 births [5%]), newborn feeding checks (treatment: 22 of 34 births [65%]; control: 5 of 21 births [24%]), and the rate of communication of danger signs to mothers and birth companions verbally (treatment: 30 of 36 births [83%]; control: 14 of 22 births [64%]) and in a written format (treatment: 3 of 24 births [13%]; control: 0 of 16 births [0%]).

**Conclusions and Relevance:**

In this cluster randomized clinical trial, health facilities that implemented the SCC with medium-intensity coaching had an increased rate of application for 5 of 36 essential birth practices compared with the control facilities. Medium-intensity coaching may not be sufficient to increase uptake of the SCC to a satisfying extent, but it may be worthwhile to assess a redesigned coaching approach prompting long-term behavioral change and, therefore, effectiveness.

**Trial Registration:**

isrctn.org Identifier: ISRCTN11041580

## Key Points

**Question:**

Can the Safe Childbirth Checklist (SCC) supplemented by medium-intensity coaching increase the application of essential birth practices to reduce maternal and perinatal mortality?

**Findings:**

In this cluster randomized clinical trial of 32 health facilities in Aceh, Indonesia, SCC use significantly increased application of 5 of 36 essential birth practices by up to 41 percentage points despite low SCC uptake.

**Meaning:**

The finding of smaller effects of the SCC on essential birth practices in settings with less intensive vs more intensive coaching supports a strengthened SCC intervention while keeping costs manageable.

## Teaser

This cluster randomized clinical trial examines the effect of implementing the Safe Childbirth Checklist with medium-intensity coaching on the performance of essential birth practices in health care facilities in Aceh, Indonesia.

## Introduction

Every year, there are approximately 600 000 intrapartum-related stillbirths worldwide, and approximately 300 000 mothers die during pregnancy or labor.^1,2^ Up to 90% of deaths among neonates occur within the first 48 hours of life.^3,4^ With regard to maternal deaths, more than 40% occur in the intrapartum period and 45% within 24 hours after the neonate is born.^5^ Most of the deaths could be preventable, and instead of access to medical care, quality of care and correct administration of treatment are the major constraints.^6^

The World Health Organization has introduced a Safe Childbirth Checklist (SCC), which targets necessary improvements to quality of care.^7^ The SCC builds on positive achievements of similar checklists in other areas of medicine, such as the Safe Surgery Checklist.^8,9^ Specifically for childbirth, the SCC consists of 4 pages and includes 4 pause points: on admission to the hospital, just before pushing or cesarean delivery, soon after birth, and before hospital discharge. At each pause point, the checklist reminds the health care staff about the essential practices and gives a brief explanation. Users follow the list point by point. The SCC was developed through a combination of expert consultation and field evaluations in 9 countries in Africa, Asia, and the Middle East.^7,10^

In an attempt to assess the SCC’s effectiveness, the World Health Organization invited practitioners and academics to evaluate the checklist in different contexts. Overall, a recent systematic review of the literature reported an association between increased application of essential birth practices and the reduction of stillbirths.^11^

Facility-based evaluations that compared outcomes before vs after use of the SCC have indicated a range of potential benefits associated with use of the checklist. Studies from India, Bangladesh, Namibia, Sri Lanka, and Mexico showed increases in the application of essential birth practices associated with checklist use,^12,13,14,15^ contributing to a reduction of perinatal mortality,^15^ improved team-level communication, quality of care under a high workload,^16^ and a satisfactory experience of birthing assistance among mothers.^17^

These studies, however, could not discern whether other confounding factors affected the results. Experimental studies aim to fill this gap with the provision of causal evidence. When assessing the effectiveness of the SCC, it is relevant to consider that implementation approaches with varying coaching intensity are compared. Low intensity includes monitoring only, whereas high intensity includes more regular (weekly or biweekly) and lengthy coaching visits as well as provision of supplies and equipment.

An evaluation of high-intensity coaching in Uttar Pradesh, India, found significant increases in the use of essential practices; however, these practices diminished after the SCC coaching ceased, and overall, significant effects on perinatal death, maternal death, or severe complications were not found.^18,19,20^ Other studies examining high-intensity coaching showed an 11% reduction in perinatal mortality in Rajasthan, India,^21^ and reduced stillbirths and perinatal mortality in Kenya and Uganda.^22^ With regard to medium-intensity interventions, another trial in Rajasthan showed a significant increase in the use of essential practices, from 6.5 of 28 practices in the control group to 17.9 in the intervention group.^23^ A trial examining low-intensity coaching in Pakistan revealed limited effects on the use of essential practices.^24^ The trials varied in size from 16 to 200 facilities.

The differential results show that the success of the SCC may depend on an adequate level of accompanying coaching, although the most cost-effective extent of coaching that also takes specific skill and resource levels into account has not been identified to our knowledge.^15^ The current trial took the suggested need for complementary training with checklist introduction into account but aimed to find a middle ground of sufficient training, allowing effective checklist use at the lowest possible implementation costs. In addition, moderate coaching costs are crucial to allow sustainable maintenance beyond the study period because effects seem to diminish once the coaching intensity decreases.^20,25^ We evaluated the effect of the SCC with medium-intensity coaching on health care workers’ performance of essential birth practices.

## Methods

This cluster randomized clinical trial was supported by facility leadership and the provincial and district health offices in Aceh, Indonesia, and written informed consent was collected before randomization took place. Before observations, enumerators introduced participating midwives and mothers to the study, described benefits and risks, and provided contact details of the researchers in case participants had questions or wished to withdraw their consent later. In addition, midwives and mothers read and signed individual informed consent forms. The study protocol was screened and approved by the ethics committees of Syiah Kuala University, Banda Aceh, Indonesia, and Georg-August University of Göttingen, Germany, before patient enrollment. After data collection and before data analysis, the study was registered retrospectively (ISRCTN11041580 and AEARCTR-0003548). The trial protocol is given in Supplement 1. The study followed the Consolidated Standards of Reporting Trials (CONSORT) reporting guideline.

### Trial Design, Randomization, and Masking

According to an optimization approach, the research team matched facilities in 2 groups and assigned treatment randomly.^26^ The matching approach minimizes the mean squared error on covariates and potential outcomes between 2 groups before treatment. In contrast to pairwise matching approaches, groupwise matching offers an advantage because attrition of 1 unit would not demand dropping the matched observation. After estimating the optimal groups, we assigned treatment randomly and introduced the SCC in 16 (50%) of the 32 total health care facilities at the facility level. Owing to the comprehensiveness of the intervention, no blinding of study participants was applicable.

### Trial Setup and Participants

**Figure 1.**
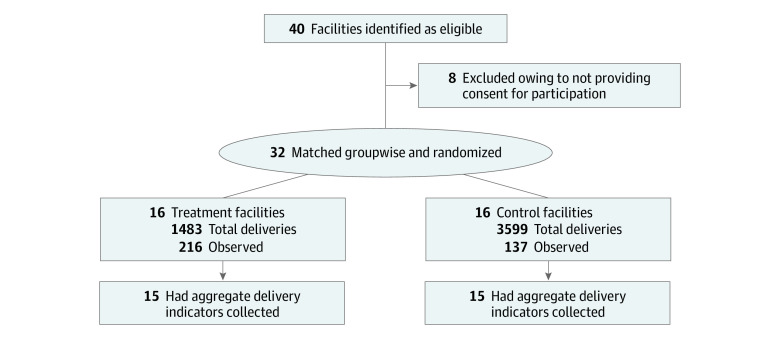
Flow Diagram for Randomization and Data Collection

An international research team from Syiah Kuala University, Georg-August University of Göttingen, and Ruprecht-Karls-Universität Heidelberg conducted the trial. With a neonatal mortality rate of 34 deaths per 1000 live births, Aceh had the nineteenth highest mortality rate among the 34 provinces in Indonesia.^27^ Qualitative research complementing the data analyzed in this article with perspectives from health care workers and mothers underlines the limited quality of care.^28^ Despite those deficiencies, because of the large health system investments after the tsunami in 2005, existing quality management practices, and relatively high levels of equipment and supplies, Aceh was considered a promising setting for SCC introduction.^28,29,30^ Starting in October 2016, the SCC was implemented in the districts of Aceh Besar, Banda Aceh, and Bireuen. Facilities were eligible if they offered at least basic emergency obstetric and newborn care services. Of 40 eligible facilities, 8 did not participate in the study because they did not have any births during the previous month or they asked for financial compensation, which we could not provide (Figure 1). The sampled facilities included hospitals, community health centers (puskesmas), and midwifery clinics, with a total yearly birth volume of approximately 11 000 births.

### Intervention

In collaboration with local midwives, obstetricians, and policy makers, we adapted the SCC to the Acehnese setting in terms of language and local practices as described in eFigure 2 and the Checklist Adjustment section in the eAppendix in Supplement 2. The SCC was translated to Bahasa Indonesia, including backward translation to English for quality checks (the English version is provided in eFigure 2 in Supplement 2). Following the Engage–Launch–Support model of the accompanying BetterBirth Trial in India,^20^ we introduced the SCC in October 2016. Coaches provided motivation and information on correct checklist use during a 2-hour launch event. During the launch event, a responsible lead person (checklist quality coordinator) was chosen at all facilities to supervise use of the SCC. During the 6 months of observation, trained coaches visited each health care facility 11 times to provide new SCC copies, collect completed SCCs, and provide feedback. Coaching was supported by 2 meetings with the checklist quality coordinators to exchange best practices (the trial protocol in Supplement 1 and eTable 8 in Supplement 2 provide more details about the trial). Although no other intervention took place in the selected facilities at the same time, the SCC treatment added to existing recording and quality-management practices.

### Data Collection and Outcomes

Between August and October 2016, the research team collected baseline data, including location of the facility, accreditation status, private or public ownership, insurance coverage, and facility type. Outcomes were measured from March to April 2017. Treatment assignment balanced these variables across the treatment and control groups, and none of the differences were statistically significant. At the final data collection in April 2017, 1 control facility was lost to follow-up because it closed after trial initiation and 1 treatment facility was excluded because it did not report any births during the observation period. As primary outcomes, we collected the SCC use at each of the 4 pause points during the birthing process and the application of essential birth practices (measured separately by item). We trained a group of nurses and midwives intensively to ensure a coherent recording for observations. To reduce bias from increased efforts to prove the SCC’s effectiveness or ineffectiveness, we introduced the observations to health care workers as a general screening of birth practices and did not highlight the evaluation of SCC use. To reduce observation bias, observation teams were present for long-term observations (usually 24 hours a day for 7 days). We deployed observers in a subset of 9 treatment and 8 control facilities with sufficient case numbers, resulting in a random sample of 353 births observed (137 in control facilities and 216 in treatment facilities). The number of observed births per pause point was lower because changes in observation team shifts and referrals prevented us from observing the full birthing process for all births. As secondary outcomes, we collected facility-level mortality and numbers of complications from the facility registries.

### Sample Size

Assuming an SCC use rate of 50% in treatment facilities and a doubling of the use of essential birth practices as suggested in previous trials,^12,23^ the study was designed and powered for analyses of the essential birth practices (eTable 1 in Supplement 2). The study was not powered to detect effects on morbidity and mortality (eTable 2 in Supplement 2).

### Statistical Analysis

Data were analyzed from January 2020 to October 2021. As a primary outcome measure, we examined the application of essential practices.^20,23^ The basic intention-to-treat (ITT) estimates used an approach of a binary outcome of essential practices on a binary indicator of treatment assignment via ordinary least-squares regressions, and thus, they represent linear probability models for binary outcome measures. For a second set of regressions, we considered a vector of covariates at the facility level (facility type, urban or rural location, provision of comprehensive emergency obstetric and newborn care services, and district as indicated by a binary variable). Owing to the random treatment assignment, covariates were not strictly required, and we reported results with and without those variables. The ITT estimates in the main results considered the initial random treatment assignment at the facility level irrespective of health care workers’ use of the checklist. Because the results indicated a lower rate of checklist use than expected, we decided post hoc to conduct an analysis of adherence. This analysis aimed to estimate the complier average causal effect (CACE) on the individuals who adhered to the intervention (ie, the CACE).^31^ A checklist was considered to be adhered to when the observers noted that the attending staff had filled out the SCC or were observed referring to the SCC during the birth process. The complier average causal effect estimator builds on a 2-step approach.^32^ Adherence to the treatment was predicted in the first stage. In the second stage, essential birth practices (birth level) and health outcomes (facility level) were regressed on the predicted adherence.

Despite the aforementioned caveat regarding statistical power, we also estimated treatment effects for morbidity and mortality outcomes. We used equations 1 to 3 in the eAppendix in Supplement 2 and accounted for the rare events nature of mortality via a penalized maximum likelihood logistic (Firth logit) regression.^33^ If applicable, clustered SEs accounted for intracluster correlation at the facility level. Given the multiple hypotheses tested, we also analyzed whether effects were significant at the 10% level when adjusting for the false discovery rate using the Benjamini-Hochberg method.^25,34^ Analyses were performed using Stata, version 16.0 (StataCorp LLC). Significance was set at 2-tailed *P* < .10.

For a qualitative analysis, F.D. and S.S.S. conducted 2 complementary focus group discussions with midwives at treatment facilities concerning their experience with the SCC. Questions focused on the coaching process and the perceived quality of care.

## Results

### Facility Characteristics

A total of 32 facilities (16 treatment and 16 control) participated in the trial. Baseline demographic data on health outcomes and facility characteristics are presented in Table 1. The main variables of interest were annual birth volume (mean [SD], 348.75 [677.04] births), maternal mortality (mean [SD], 1.26 [3.96] deaths per 100 000 individuals), neonatal mortality (mean [SD], 0.02 [0.01] deaths per 1000 individuals), and stillbirth rates (mean [SD], 0.01 [0.02] stillbirths per 1000 deliveries). When considering hospitals and primary health care facilities, variables had large SDs. However, given our matching approach, variables were balanced. Facility-specific numbers are provided in eTable 7 in Supplement 2.

**Table 1.** Balance of Facility Characteristics

Variable	Facilities^a^			Difference between treatment and control	*P* value
	All (N = 32)	Treatment (n = 16)	Control (n = 16)		
Binary variables and values^b^					
Hospital (1) and primary health care (0)	0.38 (0.49)	0.44 (0.51)	0.33 (0.49)	0.10	.57
Bireuen (1) and Aceh Besar and Banda Aceh (0)	0.41 (0.50)	0.38 (0.50)	0.47 (0.52)	−0.09	.62
Urban (1) and rural (0)	0.41 (0.50)	0.38 (0.50)	0.47 (0.52)	−0.09	.62
Public (1) and private (0)	0.66 (0.48)	0.56 (0.51)	0.80 (0.41)	−0.24	.17
CEmONC 24 h (1)^c^	0.25 (0.44)	0.19 (0.40)	0.33 (0.49)	−0.15	.37
Insurance (Badan Penyelenggara Jaminan Sosial) coverage	0.91 (0.30)	0.88 (0.34)	1.00 (0.00)	−0.13	.17
Annual births	348.75 (677.04)	258.88 (404.53)	467.67 (898.19)	−208.79	.41
Maternal mortality, deaths per 100 000 individuals	1.26 (3.96)	0.78 (2.60)	1.89 (5.25)	−1.12	.46
Stillbirth, deaths per 1000 individuals	0.01 (0.02)	0.01 (0.02)	0.01 (0.03)	0.00	.80
Neonatal mortality, deaths per 1000 individuals	0.02 (0.01)	0.00 (0.01)	0.00 (0.01)	0.00	.75

Abbreviation: CEmONC, comprehensive emergency obstetric and newborn care.

^a^ Data are presented as mean (SD) number of facilities unless otherwise indicated. The original treatment assignment also included complication and morbidity rates, which are not reported for brevity. Given the balancing approach, there were no statistically significant differences with respect to those variables.

^b^ The 0 and 1 indicate binary and categorical variables with 1 and 0 as a value. For example, if the variable equals 1, the observation is a hospital; if the variable equals 0, the observation is a primary health care facility.

^c^ The CEmONC covers safe blood transfusion and performance of cesarean sections in addition to basic emergency obstetric and newborn care.

### Adherence to Checklist Use

The staff in treatment facilities filled out 1036 SCCs for 1483 births for an adherence rate of 70%. During observations, practitioners used the SCC in 72 of 277 births (26%), which suggests that SCCs were partly filled out after birth. Observed checklist use during birth might be a more relevant uptake indicator.

### Adherence to Essential Birth Practices

We observed 277 birth practices at the time of admission to the hospital, 210 just before pushing (or before cesarean delivery), and 207 soon after birth. Observations before hospital discharge were more challenging to conduct because many facilities in Aceh provided postnatal care in a unit distinct from the birthing unit. We collected data for 58 mothers in total. Regression results in Table 2 and Table 3 are based on individual birth observations but can be interpreted as the proportion of births in which the different essential practices were conducted. Results for the ITT analysis are provided in Table 2 and for the CACE analysis in Table 3. Results are shown both with and without covariates.

**Table 2.** Main Regression Results for Performance of Specific Essential Practices From the ITT Analysis

Essential birth practice	Proportion of births observed, No. (%)		ITT regression coefficient (95% CI)		Benjamini-Hochberg significance
	Control	Treatment	Without covariates	With covariates	
**At hospital admission**					
Soap and water used	58/112 (52)	79/165 (48)	−0.04 (−0.22 to 0.15)	−0.02 (−0.16 to 0.13)	No
Gloves worn	100/105 (96)	133/138 (97)	0.01 (−0.06 to 0.08)	0.02 (−0.04 to 0.08)	No
Referral checked	99/112 (88)	142/156 (91)	0.03 (−0.18 to 0.23)	−0.08 (−0.20 to 0.03)	No
Partograph started	21/92 (23)	16/108 (15)	−0.08 (−0.19 to 0.03)	−0.03 (−0.15 to 0.09)	No
Maternal temperature taken	25/109 (23)	30/145 (21)	−0.02 (−0.24 to 0.20)	0.05 (−0.06 to 0.15)	No
Maternal blood pressure taken	102/108 (94)	125/151 (83)	−0.12 (−0.26 to 0.03)	0.01 (−0.08 to 0.10)	No
Birth companion encouraged	103/107 (96)	151/157 (96)	−0.00 (−0.03 to 0.03)	−0.01 (−0.06 to 0.04)	No
Danger signs informed	79/107 (74)	136/155 (88)	0.14 (0.14 to 0.14)^a^	0.11 (−0.02 to 0.24)^b^	Yes
**Just before pushing (or cesarean delivery)**					
Soap and water used	45/71 (63)	36/71 (51)	−0.13 (−0.57 to 0.31)	−0.05 (−0.54 to 0.43)	No
Gloves worn	67/71 (94)	68/72 (94)	0.00 (−0.05 to 0.05)	−0.02 (−0.13 to 0.08)	No
Maternal temperature taken	12/71 (17)	3/68 (4)	−0.13 (−0.42 to 0.17)	−0.01 (−0.18 to 0.16)	No
Maternal blood pressure taken	30/69 (43)	24/68 (35)	−0.08 (−0.39 to 0.23)	0.04 (−0.58 to 0.67)	No
Assistant ready	69/69 (100)	67/71 (94)	−0.06 (NA)	−0.12 (−0.31 to 0.08)	No
Practitioner checked for multiple birth after birth of firstborn	62/69 (90)	59/65 (91)	0.01 (−0.14 to 0.16)	−0.01 (−0.20 to 0.18)	No
Oxytocin administered	67/70 (96)	61/64 (95)	−0.00 (−0.08 to 0.07)	−0.06 (−0.27 to 0.15)	No
Newborn dried	72/72 (100)	68/70 (97)	−0.03 (NA)	−0.07 (−0.22 to 0.09)	No
Reaction to neonatal danger signs	49/72 (68)	32/70 (46)	−0.22 (−0.52 to 0.08)	−0.02 (−0.19 to 0.15)	No
All supplies available	10/137 (7)	6/73 (8)	0.02 (−0.11 to 0.14)	0.01 (−0.12 to 0.15)	No
**Soon after birth (within 1 h)**					
Mother checked for bleeding	66/70 (94)	105/110 (95)	0.00 (−0.06 to 0.06)	−0.00 (−0.24 to 0.24)	No
Maternal temperature taken	10/66 (15)	15/106 (14)	−0.01 (−0.17 to 0.15)	0.09 (−0.24 to 0.42)	No
Maternal blood pressure taken	34/68 (50)	60/108 (56)	0.07 (−0.43 to 0.56)	0.05 (−1.13 to 1.24)	No
Newborn weight taken	67/69 (97)	136/137 (99)	0.02 (−0.06 to 0.11)	0.01 (−0.10 to 0.12)	No
Newborn temperature taken	6/65 (9)	39/133 (29)	0.20 (−0.01 to 0.41)^b^	0.14 (−0.09 to 0.37)	No
Newborn respiratory rate taken	21/70 (30)	79/137 (58)	0.28 (0.07 to 0.50)^c^	0.09 (−0.22 to 0.41)	No
Skin-to-skin care					
Initiated	46/66 (70)	58/106 (55)	−0.15 (−0.45 to 0.15)	−0.20 (−0.45 to 0.05)	No
Maintained for 1 h	29/63 (46)	36/111 (32)	−0.15 (−0.34 to 0.05)	−0.17 (−0.41 to 0.07)	No
Breastfeeding initiated	35/66 (53)	51/110 (46)	−0.07 (−0.27 to 0.14)	−0.13 (−0.31 to 0.05)	No
**Before discharge**					
Maternal temperature taken	2/20 (10)	2/27 (7)	−0.03 (−0.27 to 0.22)	−0.02 (−0.48 to 0.43)	No
Maternal blood pressure taken	11/21 (52)	14/27 (52)	−0.01 (−0.15 to 0.14)	−0.05 (−0.31 to 0.22)	No
Mother checked for bleeding	15/21 (71)	26/29 (90)	0.18 (−0.10 to 0.46)	0.08 (−0.16 to 0.33)	No
Newborn temperature taken	1/20 (5)	9/31 (29)	0.24 (0.01 to 0.47)^c^	0.38 (0.38 to 0.38)^a^	Yes
Newborn feeding checked	5/21 (24)	22/34 (65)	0.41 (0.06 to 0.76)^c^	0.17 (−0.00 to 0.35)^b^	Yes
Family planning discussed	4/22 (18)	11/26 (42)	0.24 (−0.12 to 0.61)	0.09 (−0.18 to 0.35)	No
Follow-up arranged	12/20 (60)	22/28 (79)	0.19 (−0.12 to 0.49)	0.03 (−0.51 to 0.58)	No
Danger signs					
Verbal	14/22 (64)	30/36 (83)	0.20 (−0.24 to 0.63)	0.24 (0.03 to 0.44)^c^	Yes
Written	0/16 (0)	3/24 (12)	0.13 (NA)	0.14 (0.00 to 0.28)^c^	Yes

Abbreviations: ITT, intention to treat; NA, not applicable.

^a^ *P* < .01.

^b^ *P* < .10.

^c^ *P* < .05.

**Table 3.** Main Regression Results for Performance of Specific Essential Practices From the CACE Analysis

Essential birth practice	Proportion of births observed, No. (%)		CACE regression coefficient (95% CI)		Benjamini-Hochberg significance
	Control	Treatment	Without covariates	With covariates	
**At hospital admission**					
Soap and water used	58/112 (52)	79/165 (48)	−0.10 (−0.56 to 0.37)	−0.05 (−0.47 to 0.37)	No
Gloves worn	101/105 (96)	134/138 (97)	0.02 (−0.16 to 0.21)	0.05 (−0.12 to 0.22)	No
Referral checked	99/112 (88)	142/156 (91)	0.07 (−0.46 to 0.59)	−0.25 (−0.60 to 0.10)	No
Partograph started	21/92 (23)	16/108 (15)	−0.20 (−0.47 to 0.08)	−0.10 (−0.46 to 0.26)	No
Maternal temperature taken	25/109 (23)	30/145 (21)	−0.04 (−0.58 to 0.50)	0.13 (−0.16 to 0.43)	No
Maternal blood pressure taken	102/108 (94)	125/151 (83)	−0.30 (−0.68 to 0.08)	0.03 (−0.24 to 0.30)	No
Birth companion encouraged	103/107 (96)	151/157 (96)	−0.00 (−0.08 to 0.08)	−0.03 (−0.18 to 0.11)	No
Danger signs informed	79/107 (74)	136/155 (88)	0.35 (0.35 to 0.35)^a^	0.31 (−0.05 to 0.67)^b^	Yes
**Just before pushing (or cesarean delivery)**					
Soap and water used	45/71 (63)	36/71 (51)	−0.47 (−2.11 to 1.17)	−0.31 (−3.21 to 2.58)	No
Gloves worn	67/71 (94)	68/72 (94)	0.00 (−0.18 to 0.19)	−0.14 (−0.79 to 0.51)	No
Maternal temperature taken	12/71 (17)	3/68 (4)	−0.47 (−1.60 to 0.66)	−0.08 (−1.13 to 0.97)	No
Maternal blood pressure taken	30/69 (43)	24/68 (35)	−0.30 (−1.43 to 0.83)	0.28 (−3.71 to 4.26)	No
Assistant ready	69/69 (100)	67/71 (94)	−0.21 (−0.61 to 0.19)	−0.70 (−1.87 to 0.47)	No
Second newborn checked	62/69 (90)	59/65 (91)	0.04 (−0.62 to 0.69)	−0.06 (−1.24 to 1.12)	No
Oxytocin administered	67/70 (96)	61/64 (95)	−0.02 (−0.33 to 0.30)	−0.37 (−1.62 to 0.89)	No
Newborn dried	72/72 (100)	68/70 (97)	−0.13 (−0.44 to 0.19)	−0.42 (−1.38 to 0.54)	No
Reaction to neonatal danger signs	49/72 (68)	32/70 (46)	−0.98 (−2.29 to 0.33)	−0.15 (−1.24 to 0.95)	No
All supplies available	10/137 (7)	6/73 (8)	0.06 (−0.41 to 0.53)	0.07 (−0.80 to 0.94)	No
**Soon after birth (within 1 h)**					
Mother checked for bleeding	66/70 (94)	105/110 (95)	0.01 (−0.22 to 0.24)	−0.00 (−0.95 to 0.95)	No
Maternal temperature taken	10/66 (15)	15/106 (14)	−0.04 (−0.67 to 0.59)	0.40 (−1.00 to 1.80)	No
Maternal blood pressure taken	34/68 (50)	60/108 (56)	0.24 (−1.58 to 2.06)	0.21 (−4.46 to 4.89)	No
Newborn weight taken	67/69 (97)	136/137 (99)	0.08 (−0.24 to 0.40)	0.02 (−0.41 to 0.46)	No
Newborn temperature taken	6/65 (9)	39/133 (29)	0.77 (−0.05 to 1.59)^b^	0.63 (−0.43 to 1.69)	No
Newborn respiratory rate taken	21/70 (30)	79/137 (58)	1.11 (0.27 to 1.95)^c^	0.37 (−0.91 to 1.65)	No
Skin-to-skin care					
Initiated	46/66 (70)	58/106 (55)	−0.60 (−1.79 to 0.59)	−0.83 (−1.87 to 0.20)	No
Maintained for 1 h	29/63 (46)	36/111 (32)	−0.58 (−1.37 to 0.21)	−0.77 (−1.83 to 0.29)	No
Breastfeeding initiated	35/66 (53)	51/110 (46)	−0.27 (−1.07 to 0.54)	−0.54 (−1.30 to 0.23)	No
**Before discharge**					
Maternal temperature taken	2/20 (10)	2/27 (7)	−0.07 (−0.68 to 0.55)	−0.09 (−1.82 to 1.64)	No
Maternal blood pressure taken	11/21 (52)	14/27 (52)	−0.01 (−0.37 to 0.34)	−0.13 (−0.84 to 0.58)	No
Mother checked for bleeding	15/21 (71)	26/29 (90)	0.53 (−0.29 to 1.35)	0.26 (−0.51 to 1.03)	No
Newborn temperature taken	1/20 (5)	9/31 (29)	0.70 (0.02 to 1.38)^c^	1.31 (1.31 to 1.31)^a^	Yes
Newborn feeding checked	5/21 (24)	22/34 (65)	1.23 (0.18 to 2.27)^c^	0.58 (−0.01 to 1.17)^b^	Yes
Family planning discussed	4/22 (18)	11/26 (42)	0.62 (−0.32 to 1.56)	0.34 (−0.66 to 1.34)	No
Follow-up arranged	12/20 (60)	22/28 (79)	0.50 (−0.33 to 1.32)	0.10 (−1.48 to 1.68)	No
Danger signs					
Verbal	14/22 (64)	30/36 (83)	0.55 (−0.67 to 1.77)	0.73 (0.08 to 1.37)^c^	Yes
Written	0/16 (0)	3/24 (13)	0.44 (−0.28 to 1.15)	0.57 (0.02 to 1.12)^c^	Yes

Abbreviation: CACE, complier average causal effect.

^a^ *P* < .01.

^b^ *P* < .10.

^c^ *P* < .05.

After correction for false-discovery rates, significantly positive effects were revealed for 5 of 36 essential practices, including communication of danger signs at admission (treatment: 136 of 155 births [88%]; control: 79 of 107 births [74%]), measurement of neonatal temperature (treatment: 9 of 31 births [29%]; control: 1 of 20 births [5%]), newborn feeding checks (treatment: 22 of 34 births [65%]; control: 5 of 21 births [24%]), and the rate of communication of danger signs to mothers and birth companions verbally (treatment: 30 of 36 births [83%]; control: 14 of 22 births [64%]) and in a written format (treatment: 3 of 24 births [12%]; control: 0 of 16 births [0%]) (Table 2). Specifically, there was a statistically significant positive effect on the degree to which mothers or their companions were informed at hospital admission about the identification of danger signs (coefficient without covariates: 0.35 [95% CI, 0.35 to 0.35]; coefficient with covariates: 0.31 [95% CI, −0.05 to 0.67]). Whereas this information was provided in 79 of 107 births (74%) in control facilities, the proportion was 136 of 155 births (88%) in treatment facilities.

No effect was observed at the pause point just before pushing (or before cesarean delivery) or soon after birth (within 1 hour) when adjusting for multiple hypotheses testing. Adherence rates were 4% (9 of 210 births) for the pause point just before pushing (or before cesarean delivery) and 17% (35 of 207 births) for the pause point soon after birth (within 1 hour).

At the final pause point (before hospital discharge), we identified positive treatment effects on newborns’ quality of care: newborns’ temperatures were taken in 1 of 20 births (5%) in the control facilities compared with 9 of 31 births (29%) in the treatment facilities, and the frequency of newborn feeding checks was 41 percentage points higher in treatment facilities (22 of 34 newborns [65%]) than in control facilities (5 of 21 newborns [24%]). Improvements were measured both for the treatment group in general and among only facilities that adhered to the checklist. The SCC adherence rate was lowest (21% [12 of 58 births]) at the hospital discharge pause point. Consistent with the findings from the first pause point, increases in treatment facilities were found in the rates of verbal (from 14 of 22 births [64%] in the control group to 30 of 36 births [83%] in the treatment group) and written (from 0 of 16 births in the control group to 3 of 24 births [13%] in the treatment group) communication of danger signs at the fourth pause point.

Consistent with the quantitative findings, the qualitative research findings indicated the role of the SCC as a reminder, particularly with regard to the measurement of vital signs. During focus group discussions, midwives reported that easy-to-communicate danger signs were a major job aid. However, in line with low observed adherence rates, the qualitative research results suggested that a perceived overload in paperwork constrained uptake of the SCC.

### Mortality and Morbidity

**Figure 2.**
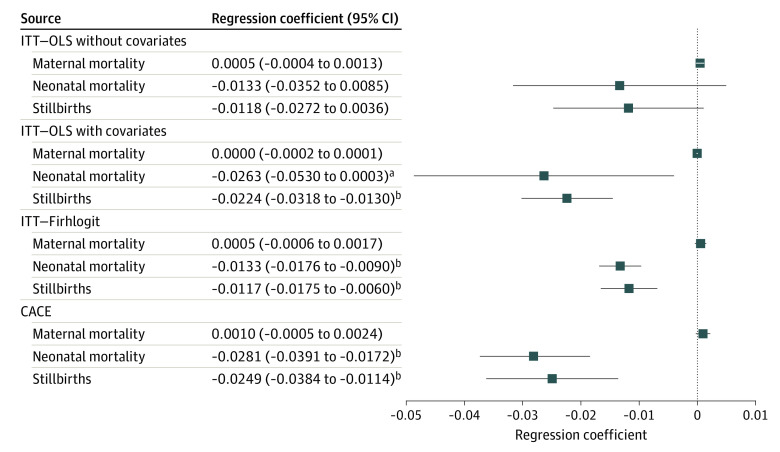
Main Regression Results for Health Outcomes Squares indicate coefficient estimates, and whiskers indicate 90% CIs. CACE, complier average causal effect; ITT, intention to treat; and OLS, ordinary least squares. ^a^*P* < .10. ^b^*P* < .01.

Results for the secondary outcomes of maternal and perinatal mortality for 5778 births for the corresponding estimators are shown in Figure 2. Only 1 maternal death was recorded in all facilities during the 6-month intervention period; thus, the point estimate was not statistically significant.

In contrast, the point estimates for neonatal mortality and stillbirths were consistently negative. Whereas the ITT estimate was not significant, coefficients were significant after we accounted for covariates, considered the rare events nature of mortality via a Firth logit estimator, or used the CACE estimator. Coefficients ranged from a reduction in the rate of stillbirths from –0.012 (95% CI, –0.018 to –0.006) with Firth logit to –0.025 (95% CI, –0.038 to –0.011) with the CACE approach. For neonatal mortality rates, those estimates ranged from –0.013 (95% CI, –0.018 to –0.009) with Firth logit to –0.028 (95% CI, –0.039 to –0.017) with for the CACE approach (eTables 3 and 6 in Supplement 2). This outcome suggests that the SCC may reduce neonatal mortality and stillbirths, particularly because point estimates were largest once we considered facilities that adhered to the checklist (Figure 2).

We also calculated estimates at the facility level (eTable 4 in Supplement 2), which allowed us to consider complication rates (a list of considered complications is provided in eTable 9 in Supplement 2). Although facility-level results were generally consistent with the main results, the individual-level regressions were more comparable with the data from the primary outcome analyses. For robustness, eFigure 1 and eTable 5 in Supplement 2 show the results of constraining the secondary outcome regressions to the reduced sample of the primary outcome analyses, which were generally consistent with the previous findings.

## Discussion

Responding to the need for improvements in the quality of care during childbirth, particularly in resource-constrained settings, the World Health Organization designed the SCC as a low-cost intervention to improve safety in the birth process.^6^ In a medium-resource setting, this study found limited effects of the SCC with medium-intensity coaching on the application of essential birth practices. Adherence increased for 5 of 36 birth practices, specifically regarding newborn care and communication of danger signs. This result is specifically interesting because several mothers indicated during the qualitative research at baseline (ie, before treatment introduction) that they felt insufficiently informed about danger signs.^28^ Although effects on complications and maternal mortality were not significant in the ITT analysis, we found a statistically significant reduction in neonatal mortality among facilities that adhered to the checklist. Adherence with the SCC was 39%, comparable with the rate found in other evaluations with limited coaching intensity^16^ but lower than that in a trial with more intensive training and coaching, which reported adherence of 86%.^23^ Similarly, studies^22^ with substantially more intensive and frequent SCC coaching (eg, the BetterBirth Trial in Uttar Pradesh, India^20^) also found greater increases in application of essential practices. However, it seems important to consider observation bias because the assessed application of SCC items substantially differs when observations are made by coaches vs independent observers,^20,35^ as was found in this trial. Accompanying qualitative research suggested that a perceived overload in paperwork constrained uptake of the SCC.^36^

### Limitations

This study has limitations. Because the trial was well powered for adherence to essential practices but not for mortality outcomes, the mortality results should be interpreted with caution. Moreover, we measured effects of the intervention for 6 months, whereas long-term effects might increase or decrease over time owing to learning curves or the phasing out of coaching.^20^

## Conclusions

In this cluster randomized clinical trial, health facilities that implemented the SCC with medium-intensity coaching had an increased rate of application for 5 of 36 essential birth practices compared with the control facilities. Based on the findings, medium-intensity coaching may not be sufficient to increase uptake of the SCC to a satisfying extent. The significant health effects among patients at the facilities that adhered to the checklist, however, suggest that a redesigned coaching approach capable of prompting long-term behavioral change may be worthwhile.^20^ In addition to a strengthened coaching approach, our qualitative research findings suggest aligning the SCC with existing quality management and recording tools (eg, attaching it to the patient file) to improve ease of checklist use and prevent an overload of paperwork.^36^

## Notes

Supplement 1.
Trial Protocol

Supplement 2. **eAppendix.** Supplementary Materials **eFigure 1.** Mortality and Morbidity (Subsample of Facilities Where Observations Were Conducted) **eFigure 2.** Adapted SCC **eTable 1.** Minimal Detectable Effect Sizes for Applied Essential Practices (Different % of Take-up) **eTable 2.** Minimal Detectable Effect Sizes for Health Outcomes **eTable 3.** Mortality (Individual Births) **eTable 4.** Mortality and Morbidity (Facility Level Rates) **eTable 5.** Mortality and Morbidity (Subsample of Facilities Where Observations Were Conducted) **eTable 6.** Point Estimates for Covariates **eTable 7.** Case Numbers per Facility **eTable 8.** Depiction of Safe Childbirth Checklist and Research Team Exposure Among Treatment and Control Group **eTable 9.** Background Information—Complications

Supplement 3.
Data Sharing Statement

## References

[1] Hug L, Sharrow D, Yo D. Levels & trends in child mortality. United Nations Inter-agency Group for Child Mortality Estimation Report 2017. Accessed February 20, 2021. https://documents1.worldbank.org/curated/en/358381508420391876/pdf/120551-REVISED-PUBLIC-IGME-report-2017-child-mortality-final.pdf

[2] Alkema L, Chou D, Hogan D, ; United Nations Maternal Mortality Estimation Inter-agency Group Collaborators and Technical Advisory Group. Global, regional, and national levels and trends in maternal mortality between 1990 and 2015, with scenario-based projections to 2030: a systematic analysis by the UN Maternal Mortality Estimation Inter-Agency Group. Lancet. 2016;387(10017):462-474. doi:10.1016/S0140-6736(15)00838-7 26584737 PMC5515236

[3] Liu L, Oza S, Hogan D, . Global, regional, and national causes of child mortality in 2000-13, with projections to inform post-2015 priorities: an updated systematic analysis. Lancet. 2015;385(9966):430-440. doi:10.1016/S0140-6736(14)61698-6 25280870

[4] Lawn JE, Lee ACC, Kinney M, . Two million intrapartum-related stillbirths and neonatal deaths: where, why, and what can be done? Int J Gynaecol Obstet. 2009;107(suppl 1):S5-S18, S19. doi:10.1016/j.ijgo.2009.07.016 19815202

[5] Requejo JH, Bryce J, Barros AJ, . Countdown to 2015 and beyond: fulfilling the health agenda for women and children. Lancet. 2015;385(9966):466-476. doi:10.1016/S0140-6736(14)60925-9 24990815 PMC7613194

[6] Kruk ME, Gage AD, Arsenault C, . High-quality health systems in the Sustainable Development Goals era: time for a revolution. Lancet Glob Health. 2018;6(11):e1196-e1252. doi:10.1016/S2214-109X(18)30386-3 30196093 PMC7734391

[7] Spector JM, Agrawal P, Kodkany B, . Improving quality of care for maternal and newborn health: prospective pilot study of the WHO Safe Childbirth Checklist program. PLoS One. 2012;7(5):e35151. doi:10.1371/journal.pone.0035151 22615733 PMC3353951

[8] Gawande A. The Checklist Manifesto. Metropolitan Books; 2009.

[9] Haynes AB, Weiser TG, Berry WR, ; Safe Surgery Saves Lives Study Group. A surgical safety checklist to reduce morbidity and mortality in a global population. N Engl J Med. 2009;360(5):491-499. doi:10.1056/NEJMsa0810119 19144931

[10] Spector JM, Lashoher A, Agrawal P, . Designing the WHO Safe Childbirth Checklist program to improve quality of care at childbirth. Int J Gynaecol Obstet. 2013;122(2):164-168. doi:10.1016/j.ijgo.2013.03.022 23742897

[11] Tolu LB, Jeldu WG, Feyissa GT. Effectiveness of utilizing the WHO Safe Childbirth Checklist on improving essential childbirth practices and maternal and perinatal outcome: a systematic review and meta-analysis. PLoS One. 2020;15(6):e0234320. doi:10.1371/journal.pone.0234320 32530940 PMC7292415

[12] Hirschhorn LR, Semrau K, Kodkany B, . Learning before leaping: integration of an adaptive study design process prior to initiation of BetterBirth, a large-scale randomized controlled trial in Uttar Pradesh, India. Implement Sci. 2015;10(117):117. doi:10.1186/s13012-015-0309-y 26271331 PMC4536663

[13] Deshmukh VL, Chahande PD, Shirale T, Yeilka KY. WHO Childbirth Checklist 2012: impact on maternal and newborn care evaluation. Int J Res Anal. 2016;4(1):113-129.

[14] Nababan HY, Islam R, Mostari S, . Improving quality of care for maternal and newborn health: a pre-post evaluation of the Safe Childbirth Checklist at a hospital in Bangladesh. BMC Pregnancy Childbirth. 2017;17(1):402. doi:10.1186/s12884-017-1588-x 29202714 PMC5716057

[15] Kabongo L, Gass J, Kivondo B, Kara N, Semrau K, Hirschhorn LR. Implementing the WHO Safe Childbirth Checklist: lessons learnt on a quality improvement initiative to improve mother and newborn care at Gobabis District Hospital, Namibia. BMJ Open Qual. 2017;6(2):e000145. doi:10.1136/bmjoq-2017-000145 28959784 PMC5574260

[16] Patabendige M, Senanayake H. Implementation of the WHO Safe Childbirth Checklist program at a tertiary care setting in Sri Lanka: a developing country experience. BMC Pregnancy Childbirth. 2015;15(12):12. doi:10.1186/s12884-015-0436-0 25648543 PMC4324022

[17] Molina R, Villar de Onis J, Reyes A, . Implementation of an adapted Safe Childbirth Checklist in rural Chiapas, Mexico: an evaluation study. Lancet Glob Health. 2017;5(suppl1):S26. doi:10.1016/S2214-109X(17)30133-X

[18] Kara N, Firestone R, Kalita T, ; BetterBirth Trial Group. The BetterBirth program: pursuing effective adoption and sustained use of the WHO Safe Childbirth Checklist through coaching-based implementation in Uttar Pradesh, India. Glob Health Sci Pract. 2017;5(2):232-243. doi:10.9745/GHSP-D-16-00411 28655801 PMC5487086

[19] Semrau KE, Hirschhorn LR, Kodkany B, . Effectiveness of the WHO Safe Childbirth Checklist program in reducing severe maternal, fetal, and newborn harm in Uttar Pradesh, India: study protocol for a matched-pair, cluster-randomized controlled trial. Trials. 2016;17(1):576. doi:10.1186/s13063-016-1673-x 27923401 PMC5142140

[20] Semrau KEA, Hirschhorn LR, Marx Delaney M, ; BetterBirth Trial Group. Outcomes of a coaching-based WHO Safe Childbirth Checklist program in India. N Engl J Med. 2017;377(24):2313-2324. doi:10.1056/NEJMoa1701075 29236628 PMC5672590

[21] Varghese B, Copas A, Kumari S, . Does the Safe Childbirth Checklist (SCC) program save newborn lives? evidence from a realistic quasi-experimental study, Rajasthan, India. Matern Health Neonatol Perinatol. 2019;5(1):3. doi:10.1186/s40748-019-0098-4 30867935 PMC6397441

[22] Walker D, Otieno P, Butrick E, ; Preterm Birth Initiative Kenya and Uganda Implementation Research Collaborative. Effect of a quality improvement package for intrapartum and immediate newborn care on fresh stillbirth and neonatal mortality among preterm and low-birthweight babies in Kenya and Uganda: a cluster-randomised facility-based trial. Lancet Glob Health. 2020;8(8):e1061-e1070. doi:10.1016/S2214-109X(20)30232-1 32710862 PMC7388203

[23] Kumar S, Yadav V, Balasubramaniam S, . Effectiveness of the WHO SCC on improving adherence to essential practices during childbirth, in resource constrained settings. BMC Pregnancy Childbirth. 2016;16(1):345. doi:10.1186/s12884-016-1139-x 27825321 PMC5101814

[24] Kuhnt J, Vollmer S. WHO Safe Childbirth Checklist in Pakistan—Evidence From a Randomized Controlled Trial. Mimeo; 2017. 10.1016/j.ssmph.2023.101495 PMC10550752 37808230

[25] Anderson ML. Multiple inference and gender differences in the effects of early intervention: a reevaluation of the Abecedarian, Perry Preschool, and Early Training projects. J Am Stat Assoc. 2008;103(484):1481-1495. doi:10.1198/016214508000000841

[26] Schneider SO, Schlather M. A new approach to treatment assignment for one and multiple treatment groups. Courant Research Center Discussion Paper No. 228. 2017. Accessed August 10, 2020. http://www2.vwl.wiso.uni-goettingen.de/courant-papers/CRC-PEG_DP_228b.pdf

[27] United Nations Development Programme. Provincial human development report: Aceh 2010. United Nations Development Programme. 2010. Accessed September 18, 2020. http://hdr.undp.org/sites/default/files/nhdr_aceh_2010_english.pdf

[28] Doria S, Diba F, Susanti SS, Vollmer S, Monfared IG. Mothers’ experiences of quality of care and potential benefits of implementing the WHO Safe Childbirth Checklist: a case study of Aceh Indonesia. BMC Pregnancy Childbirth. 2019;19(1):461. doi:10.1186/s12884-019-2625-8 31795951 PMC6891962

[29] Diba F, Ichsan I, Muhsin M, . Healthcare providers’ perception of the referral system in maternal care facilities in Aceh, Indonesia: a cross-sectional study. BMJ Open. 2019;9(12):e031484. doi:10.1136/bmjopen-2019-031484 31818837 PMC6924809

[30] Galvin G, Hirschhorn LR, Shaikh M, . Availability of safe childbirth supplies in 284 facilities in Uttar Pradesh, India. Matern Child Health J. 2019;23(2):240-249. doi:10.1007/s10995-018-2642-7 30430350 PMC6394529

[31] King G, Gakidou E, Imai K, . Public policy for the poor? a randomised assessment of the Mexican universal health insurance programme. Lancet. 2009;373(9673):1447-1454. doi:10.1016/S0140-6736(09)60239-7 19359034

[32] Imbens GW, Rubin DB. Estimating outcome distributions for compliers in instrumental variables models. Rev Econ Stud. 1997;64(4):555-574. doi:10.2307/2971731

[33] Firth D. Bias reduction of maximum likelihood estimates. Biometrika. 1993;80:27-38. doi:10.1093/biomet/80.1.27

[34] Benjamini Y, Hochberg Y. Controlling the false discovery rate. J R Stat Soc Ser B. 1995;57:289-300.

[35] Marx Delaney M, Maji P, Kalita T, . Improving adherence to essential birth practices using the WHO Safe Childbirth Checklist with peer coaching: experience from 60 public health facilities in Uttar Pradesh, India. Glob Health Sci Pract. 2017;5(2):217-231. doi:10.9745/GHSP-D-16-00410 28655800 PMC5487085

[36] Kaplan L, Kuhnt J, Richert K, Vollmer S. How to increase the uptake of development interventions? considering the theory of planned behaviour. Deutsches Institut für Entwicklungspolitik discussion paper 10. 2019. Accessed March 20, 2020. https://www.die-gdi.de/uploads/media/DP_10.2019.pdf doi:10.23661/dp10.2019

