# Supplementary material for: Effects of the World Health Organization Safe Childbirth Checklist on Quality of Care and Birth Outcomes in Aceh, Indonesia: A Cluster-Randomized Clinical Trial
Source: JAMA Netw Open. 2021 Dec 3;4(12):e2137168. doi: 10.1001/jamanetworkopen.2021.37168 (PMC8642783; doi:10.1001/jamanetworkopen.2021.37168)
Supplement: Supplement 1. — Trial Protocol [file jamanetwopen-e2137168-s001.pdf]

GENERAL INFORMATION	
**Protocol Number**: **18/04/2016**	
**Protocol Title: Evaluating the Impact of the WHO Safe Childbirth Checklist on the Quality of Care and Birth Outcomes in Public Health Facilities**	
**Version Date: 18th April 2016**	**Version Number: 1**
**Principal Investigators:**<br>J-Prof. Dr. Sebastian Vollmer<br>Platz der Göttinger Sieben 3<br>Raum 2.147<br>37073 Göttingen<br>Tel. +49 (0)551 / 39-20245<br>Fax +49 (0)551 / 39-20417<br>svollmer@uni-goettingen.de	

Table of content

I. List of Abbreviations 3
1. Summary of the project 4
2.1 Scientific background and rationale for the study 5
2.2 Significance of the research, and how it will add to existing knowledge 6
2.3 Locations of the research 6
3. Study Design 6
3.1 Study design type 6
3.2 Intervention – WHO Safe Childbirth Checklist 7
3.2.1 Content and Development 7
3.2.2 Use in SCC Intervention Units (Health Facilities) 7
3.2.3 Public Availability 8
3.3 Outcome Measures 8
3.4 Study Duration 8
3.5 Number of Participants 8
3.6 Inclusion Criteria 9
3.7 Exclusion Criteria 9
3.8 Study Procedures 9
4. Risks 10
4.1 Foreseeable risks, discomforts, and inconveniences to participants 10
4.2 Provisions in place to minimize risk 10
5. Benefits 10
5.1 Potential benefits of study participation 10
5.2 Potential benefits of the research to the local community and/or society 11
6. Consent Process 11
6.1 Institutional Consent 11
6.2 Health Institutions' Consent 11
6.3 Health Personnel Consent 12
6.4 Patient Consent 12
6.5 Delivery Skills Assessment Consent 12
7. Participant Privacy and Data Confidentiality 12
8. Statistical Analysis Plan 13
9. Research Staff 13
9.1 Main Investigation Team 13
9.2 Extended Research Team 13
9.3 Coordination Group 13
9.4 Technical Advisory Group 14
9.5 Field Investigators 14
10. Reportable Events 14
11. Vulnerable Populations 14
12. Financing 14
13. Sharing Study Results 14
II. Literature 16
III. Appendix 17

I. List of Abbreviations

KfW	Kreditbank für Wiederaufbau
LHV	Lady Health Visitor
MDG	Millennium Development Goals
OSCE	Objective Structured Clinical Examination
SCC	Safe Childbirth Checklist
TAG	Technical Advisory Group
UN	United Nations
WHO	World Health Organization

1. Summary of the project

The study aims at identifying the possible causal effect of the implementation of the adapted WHO Safe Childbirth Checklist in health facilities on the quality of care during birth and on birth outcomes, including the successful delivery of essential childbirth practices, birth complications and maternal and neonatal health outcomes. The WHO Safe Childbirth Checklist (SCC) has been developed under the WHO since 2008 to improve childbirth safety in low-resource settings. It is a 29-item list of evidence-based practices that target the major killers of mothers and newborns specifically in low- and medium-income countries. The WHO is now leading a global collaborative effort to further evaluate the SCC program in a range of contexts and invites practitioners and academics worldwide to participate in the evaluation of the instrument and to extend the evidence. The University of Goettingen is part of the WHO SCC Collaboration Network and jointly with the University Syiah Kuala, Banda Aceh support the rollout and evaluation of the SCC in health facilities in Aceh province in Indonesia.

In August and September 2016 we plan to introduce the SCC together with varying training intensity in several health facilities that are offering delivery services in Banda Aceh and Pidie/ Bireuën regency. We will randomly start to implement the SCC together with a more intensive checklist-specific training in about 50% of the health institutions selected. This study design allows us to establish two groups of health facilities (treatment and control group), which are subject to the same external conditions, except the treatment. In this way it becomes possible to causally identify the effect of the intervention on the selected outcomes by ruling out other potential channels that are unrelated to the treatment. Main outcomes will be maternal and neonatal mortality rates and birth complications as well as the applied essential practices listed in the checklist. If the treatment proves to be effective all participating health facilities will gain access to the instrument.

We will start in July 2016 with the adaptation process of the WHO SCC to the Indonesian context. This will include a content-wise adaptation through an expert group including local practitioners as well as the translation into Bahasa Indonesia. Crucial for the checklist's implementation is strong political support and leadership as well as local ownership with the respective health facility. To ensure a successful adaptation of the checklist in the treatment facilities all health personnel concerned with deliveries will participate in an introductory seminar where the checklist items and their use are explained in detail. This seminar will also be used to motivate the health care workers to use the checklist in their everyday work and increase their acceptance for the instrument.

Partly, the data that we will use for our analysis is already recorded in each health facility. This includes (among others) neonatal and maternal death rates, stillbirths, and some details on birth complications. In order to ensure comprehensive and consistent data we will start with workshops on data quality and data collection in all participating health facilities. Within a baseline survey in July 2016 basic characteristics of the health facility will be collected. Data collection will be on-going during the study period and focus group discussions with health personnel working at the treatment facilities will allow us to improve the adaptation and implementation process. Through repeated on-site visits the quality of the data collected by the health facilities will be monitored.

By comparing outcomes between control and treatment groups at different points in time we will be able to draw conclusions on the impact of the SCC on the outcomes and measure the exact size of the effect. Random sampling and random assignment of the health facilities to treatment and control groups will allow us to interpret mean differences in outcomes as causal effects of the intervention.

Specific Aims: Test whether an adaptation of the WHO Safe Childbirth Checklist in health facilities in Aceh province affects the quality of care during birth and birth outcomes, including rate of birth complications, maternal and neonatal morbidity and mortality rates.

2. Background

2.1 Scientific background and rationale for the study

Reductions of maternal and child mortality are both part of the Millennium Development Goals (MDGs) which highlights their importance in development policy. In 2013, worldwide 6.3 million children under the age of five died as a result of largely preventable causes. Almost half of those deaths occur during the neonatal period (Liu et al., 2014). While between 25–45% of all neonatal deaths occur within the first 24 hours after birth, up to 90% take place within the first 48 hours of newborn life (Lawn et al., 2005; Lawn et al., 2009). Every year there are 1.2 million intrapartum-related stillbirths and every day about 800 women die during pregnancy or birth. In addition, most maternal deaths occur within a rather narrow time frame: more than 40% of directly caused maternal deaths occur during the intrapartum period and 45% of maternal deaths during the postpartum period occur within the first 24 hours after delivery (WHO, 2010). The maternal mortality ratio in low- and middle-income countries is still 15 times higher than in high-income countries (United Nations MDG5 Fact Sheet, 2013). Motherless children have far lower chances of survival. In addition, there are more than one million children that survived birth complications, like birth asphyxia, but develop illnesses, which often imply learning difficulties and other disabilities. Over 300 million women have long- and short-term pregnancy- or childbirth-related sicknesses that the family is unprepared for and often has strong adverse effects on the newborn's health and survival chances (WHO, 2005). In the last years there have been improvements in most maternal and child health indicators worldwide. But neonatal mortality stagnated in most parts and compared to reductions in infant mortality rates progress was very slow. Indonesia has the highest neonatal mortality rate among the South East Asian countries (WHO & UN Children's Fund, 2004) with around 20 per 1,000 live births according to IDHS in 2012 (National Research Council, 2013). According to Population Census data the maternal mortality rate in Indonesia stood at 263 per 100,000 live births in 2010. 62% of maternal deaths occurred during the postpartum (puerperium) period, 25% during pregnancy, and 13% at delivery (National Research Council, 2013). Aceh Province has reported high prevalence of neonatal deaths with 40 per 1,000 live births and hence lies above the Indonesia national average (Ramadhan & Thabrany, 2013).

Practitioners and researchers agree on the overarching importance of safe childbirth to reduce the risks for mother and newborn. While nowadays more births take place in an institutional setting, morbidity and mortality rates have been slow to fall (Lim et al., 2010; Powell-Jackson et al., 2009; Ekirapa-Kiracho et al., 2011). Poor-quality care during birth, also during institutional births, remains and is a major contributing factor to easily preventable maternal and newborn harm, like excessive bleeding or infections. Inadequate hygienic conditions in the health institutions and low expertise of the health personnel reinforce those deficiencies. Providing high-quality delivery care based on proven clinical practices present according to Spector et al. (2013) a great opportunity to reduce the maternal and neonatal mortality and morbidity burden. Integration of checklists into clinical practice has been shown to reduce deaths and complications in intensive care medicine and surgery. Checklists bundle essential tasks into a practical format consisting of actionable items and hence, help the users to remember essential complex or neglected tasks.

Several features of childbirth make a checklist-based strategy promising: the major causes of maternal and perinatal mortality are well described; most deaths occur within a narrow time window (24 to 48 hours after birth); international guidelines for best practices exist but are not followed; and proven interventions are relatively inexpensive and easy to perform, but can be difficult to remember and execute in proper sequence (Spector et al., 2012). Additionally, most of those deaths occur in low resource settings. In the context of maternal and child health, the SCC is such a low-cost initiative that in theory can be implemented in hospitals in resource-poor settings regardless of other medical equipment. The SCC is intended for use at four critical junctures in clinical care around the time of birth: on admission of the mother to the birth facility; at the time the mother begins to push (or before caesarean delivery); soon after birth (within one hour); and before discharge. Items on the SCC

specifically address the major causes of maternal and newborn death in low- and middle-income countries. For women, these are postpartum haemorrhage, infection, obstructed labour, and hypertensive-related disorders. For newborns, these are infections, intrapartum-related hypoxic events (previously referred to as "birth asphyxia"), and complications of prematurity. Fresh stillbirths are addressed through inclusion of items that support improved intrapartum management.

2.2 Significance of the research, and how it will add to existing knowledge

While poor quality care in public health institutions has been acknowledged to be a major reason for maternal and neonatal harm, no widely applicable and effective method to encounter and solve these problems currently exists (Spector et al., 2012). In order to evaluate the possible effectiveness of the SCC and to determine limiting or enhancing environments to its successful adaptation, the WHO invites practitioners and researchers worldwide to implement the instrument in widely diverse settings. Until now, there is only evidence on the checklist's effectiveness coming from single high-level hospitals that introduced the instrument in their obstetrics unit. There have been no academics involved that secured a high-quality study set-up, implementation, and evaluation. Hence, there is missing evidence on the causal effect of the checklist on maternal and neonatal health outcomes. Currently, there is one large study underway implemented by Ariadne Labs (including the Brigham and Women's Hospital and the Harvard School of Public Health) supported by the Bill and Melinda Gates Foundation in Uttar Pradesh in India that uses a randomized control design (http://www.ariadnelabs.org/programs/betterbirth/). It is highly important to advance, the external validity of the instrument by adding information on the implementation success in different countries and levels of health facilities. Hence, as the rigorous scientific knowledge about the effectiveness of the SCC is still very limited, our study results will play an important part in the further development of an instrument to fight high maternal and neonatal mortality and morbidity rates and the decision to support the checklist's further expansion. Our study will add to existing studies by examining the checklist's effectiveness in the middle-income setting of Indonesia. Moreover, we will focus especially on the checklist's interaction with training and its impact on communicational and organizational structures.

2.3 Locations of the research

The study will be conducted in several health facilities in Banda Aceh and Pidie/Bireuën regency that are offering delivery services. If the study proves that the checklist is effective, all of the participating health facilities will adapt the instrument.

3. Study Design

3.1 Study design type

The study will be designed as a randomized controlled trial. It will comprise health facilities that are offering delivery services in Banda Aceh and Pidie/Bireuën regency. We will randomly start to implement the SCC together with a checklist specific training in about 50% of the health institutions selected for the study. This study design allows us to establish two groups of health facilities (treatment and control group) to causally identify the effect of the intervention on the selected outcomes. If the instrument proves to be effective all participating health facilities will gain access to the instrument.
During the study period we will collect quantitative data from the health facilities (e.g. number of birth complications, morbidity and mortality rates of mother and neonate, stillbirth rate) and complement this by qualitative data through focus group discussions and interviews with health personnel and patients.

Specific research questions are: Do maternal mortality and child mortality decrease in health facilities that adopt the WHO Safe Childbirth Checklist? If this is the case: By how much do maternal mortality/morbidity and child mortality/morbidity decrease in facilities that adopt the WHO Safe Childbirth Checklist? Does the effectiveness of the WHO Safe Childbirth Checklist in reducing mortality and morbidity outcomes depend on cultural context, social context, environmental circumstances (e.g., organization & communication in the delivery team), education/ training or economic wellbeing?

3.2 Intervention – WHO Safe Childbirth Checklist

3.2.1 Content and Development

The SCC has been developed by the WHO since 2008 and includes in its pilot edition 29 items (see Appendix A for pilot edition of SCC). Those items refer to essential childbirth practices that are evidence-proven and part of international guidelines for safe delivery.
The checklist development began with a comprehensive background document cataloguing the major causes of severe harm to women and newborns in low- and middle-income countries and outlining specific childbirth care practices with evidence-based patient safety benefits. The routine sequence of events during institutional childbirth was analysed and moments in the flow of care when a health worker could potentially review a checklist were considered. Themes for systematic improvements emerged that could be applied to each of these periods in the childbirth continuum, and specific evidence-based care practices were identified. Delegates discussed the flow of patient care during institutional births and proposed a draft set of "pause points", which are periods in the clinical workflow when it would be sensible for checklist users to pause and ensure that essential tasks were completed. There were four pause points identified: on admission of the mother; at the time the mother begins to push (or before caesarean delivery); soon after birth (within one hour); and before discharge. Afterwards, the delegates populated the developing checklist with the highest-impact safety practices identified in the evidence. Following this, for a period of six months beginning in January 2010, the applicability of the draft SCC was evaluated by frontline workers at 17 sites in nine countries in Sub-Saharan Africa (Kenya, Tanzania, Ghana, Nigeria, Mali), Asia (India, China, Pakistan), and the Middle East (Egypt). The aim was to obtain feedback from would-be users of the program in order to improve the tool and implementation strategy before pilot testing. Field collaborators worked with childbirth teams at their institutions to evaluate the draft SCC, and in the course of doing so were asked to modify the checklist as needed to suit their local practice.
It is recognized that a "one size fits all" checklist would not be feasible for all settings, and the SCC is intentionally not comprehensive. Modification is encouraged to reflect local practice and foster ownership, and may include content changes to specific checklist items or to the qualifying caption boxes. We are planning to adapt the pilot edition SCC to the Indonesian context with the help of experts, including renowned paediatricians and gynaecologists, midwives, lady health workers, and more coming from different health facility levels.

3.2.2 Use in SCC Intervention Units (Health Facilities)

The SCC shall be introduced in the obstetrics unit in the health facilities and is intended for use at four critical junctures in clinical care around the time of birth: on admission of the mother; at the time the mother begins to push (or before caesarean delivery); soon after birth (within one hour); and before discharge.

3.2.3 Public Availability

The WHO Safe Childbirth Checklist is only available to the collaboration members of the WHO SCC network. Until the beginning of 2015 the WHO invited members to use the SCC while exploring implementation factors. Registration for the Collaboration has now closed as the WHO draws closer to the official launch of the SCC.

3.3 Outcome Measures

The rates of maternal and neonatal mortality rates as well as stillbirths in health facilities will be assessed. This data is already reported for governmental usage in the majority of health facilities. Here, the quality of the data collection by each Intervention Unit needs to be ensured. If possible it will be tried to follow-up on patient fatality cases after discharge to be able to report postpartum mortality rates adequately. Another important outcome is birth complications, which can lead to short- and long-term morbidities of mother and child. Many checklist items are aiming at minimizing easily preventable conditions, like birth asphyxia, bacterial sepsis, and hypothermia. Related to this, we will assess whether the different checklist items, e.g., use of partograph, medication or breastfeeding are realized. We will use this information to estimate the average rate of successful delivery of essential childbirth practices per patient within each SCC Intervention Unit. This can give us an indication on the usability of the checklist items. Finally, first evidence indicated that the checklist could favourably affect the institutional setting and the communication in the health facility. Also the organizational structure of the delivery team might change. More specifically, we would like to put a specific focus on the empowerment of nurses and midwives through the use of the checklist. In this regard we would like to examine also feed-back effects as the checklist’s effectiveness might be contingent on the change in communication and organizational structure within the delivery teams.

3.4 Study Duration

The first focus group discussions with experts to adapt the checklist to the local context and workshops on data collection to ensure the data quality will start in July 2016. Afterwards, the baseline data collection, including a needs-assessment and the collection of primary and secondary outcome variables will be conducted in all SCC Intervention Units. Additionally, a random sample of patients will be interviewed. Approximately in August and September 2016, the checklist will be introduced together with a complementary training of different intensity to the random set of SCC Intervention Units in the treatment group. The primary outcome measures will be recorded continuously by all health facilities and monitored regularly by a team of enumerators. By early 2017 the endline data collection will take place, including primary and secondary outcome variables and another patient survey. This will be followed by an analysis of results. In the case that the intervention proves to be effective, the remaining SCC Intervention Units will adapt the instrument.

3.5 Number of Participants

The study will comprise all public health facilities on the level of hospitals (rumah sakit) and community health centres (puskesmas) in Banda Aceh and the regency of Pidie/Bireuën. Questionnaires with a subset of health personnel of each participating health facility will be conducted. Additionally, questionnaires with randomly selected patients will be undertaken.

3.6 Inclusion Criteria

All public health facilities in Banda Aceh and Pidie/Bireuën regency, including hospitals (rumah sakit) and community health centers (puskesmas) will be included in the study population. In this way the study can collect evidence, both in an urban setting (Banda Aceh) and a more rural region (Pidie/Bireuën).

3.7 Exclusion Criteria

Health facilities that below the level of hospitals and community health centres (puskesmas) will not be considered, as a sufficient number of cases per observation unit is needed to ensure an empirically meaningful analysis.

3.8 Study Procedures

In order to properly randomize across all SCC Intervention Units, data on staffing and equipment at health facilities as well as socio-economic patient characteristics will be used. If the woman gave her consent, the health institutions will provide us with her contact details. Out of those patients a randomly selected sample will be drawn for follow-up interviews. Some of our main outcome variables are already reported by all health facilities to the governmental agencies. Hence, in order to ensure the quality of the data reported and the comparability across institutions, in each health facility a person responsible for data collection will be selected, who then will attend a workshop on data quality and data collection. The additional data (especially secondary outcomes) will be collected in each SCC Intervention Unit by our trained enumerators. Basic characteristics of the health institution (e.g., number of beds and patients, number of deliveries, number of employees, characteristics of staff [temporary/permanent; turnover rates; profession, responsibilities], participation in other programs, organizational features, structure and use of referral system), as well as on stocks and supplies availability, equipment, birth complications, and the successful delivery of essential childbirth practices will be collected. This information is simultaneously used to conduct a needs-assessment related to essential childbirth practices. Within the health facilities, the interviewer will also ask for the satisfaction of health personnel, leadership characteristics, teamwork and hierarchies, and communication structures. Here, the written consent of the personnel will be asked and the reason for the questionnaire will be explained. Interviewers will point out that the participation is voluntary and that they may drop out at any point in time. The collection of the patient data will be conducted at the patients' home. If this turns out to be impossible, interviews might be conducted via phone. The interviewer will explain the content and scope of the study to the patient and will then ask for consent to participate in the study. Interviewers will point out that the study participation is voluntary and that they may drop out at any point in time. Afterwards the enumerators will interview the patient to collect data on her and the fathers' socioeconomic and educational background, household characteristics, birth and health history, prenatal care and her satisfaction with the birth process in the respective health facility.

After the consent of the health facility leadership to implement the SCC into clinical practice, the instrument will be introduced in all SCC Intervention Units within a standardized workshop. The instrument's use will be explained in detail and the checklist's adaptation will be practically tried out. The aim of the workshop is primarily to make sure that the checklist as such is well understood and secondly, that the motivation for the new instrument is high to ensure the checklist's use in practice. The training itself will be subject to validation via a "Delivery Skills Assessments", which will be conducted prior to the training (baseline skills) and around 4 to 6 months after the training (long-term skill gains). The assessment might include a practical evaluation of health personnel's every-day clinical work using an OSCE (Objective Structured Clinical Examination) or similar format. The OSCE is a modern type of examination often used in health sciences. It is designed to test clinical skill performance and competence in skills such as communication, clinical examination, medical

procedures and interpretation of diagnostic results. The OSCE would comprise a circuit of short (5-15min) stations, in which each candidate is examined on a one-to-one basis with one or two impartial examiners and simulated patients (actors or simulators). Each station has a different examiner. Candidates rotate through the stations and complete all the stations on their circuit. In this way, all the candidates take the same standardized stations, enabling a fair peer comparison. They will be developed, standardized and conducted by an experienced group of experts in this field. The SCC will be adapted as new standard operational procedure and hence, be obligatory to use for the health personnel. The enforcement of the use crucially depends on the leadership of the SCC Intervention Units.

Experts will visit each Intervention Unit one to two months after the checklist's introduction in order to respond to questions and problems with the instrument's use and to monitor the quality of the data reported by the institution. In case of urgent questions concerning the checklist's adaptation or use, a local contact person will be provided for the entire study period.

4. Risks

4.1 Foreseeable risks, discomforts, and inconveniences to participants

There are no foreseeable risks emerging from the SCC. Its content is based on proven international best practices. For a detailed description of the SCC please refer to section 3.1 and to appendix B. The implementation of the checklist within the SCC Intervention Units may cause some discomfort to health personnel, as they will have to get used to its standardized application in their everyday work. Questions on their work satisfaction and motivation might also cause discomfort.

4.2 Provisions in place to minimize risk

The possible high returns from using the checklist will be strongly emphasized in the introductory workshops. The interviewer will also talk about the current levels of maternal and neonatal morbidity and mortality rates within the regencies, their causes, and stressing the importance of the health personnel and power they have to reduce those. It will also be pointed out that the checklist will not be used to monitor the work of the health personnel at any time but instead that it is a tool solely developed to serve them as a reminder and hence, support them in their everyday work. The interviewer will try to motivate the health personnel and assist them with questions and concerns on the instrument's use and implementation in their everyday work. It has been shown in previous studies that the health personnel usually receive the SCC adaptation and the complementary training positively. The questionnaire (work satisfaction, motivation, leadership) will be conducted in privacy and the anonymity of the answers will be stressed. Additionally, the interviewer will point out that there will be no adverse consequences for them, also if they decide not to take part. These questions will also only be asked twice within the study period, once at the start of the instrument's use and once at the end of the study.

5. Benefits

5.1 Potential benefits of study participation

Previous studies on other checklists (e.g. surgical checklist) have shown that the checklist's adaptation led to reductions of complications arising during complex processes at the health facility and to decreased morbidity and mortality outcomes. The birth process resembles surgical procedures in its complexity and in the existence of proven interventions to reduce adverse outcomes but the difficulty to remember all essential steps to take. First studies on the SCC report an increase in the essential

childbirth practices that are included in the checklist. There is a strong established relationship between those practices and maternal and neonatal health outcomes. Hence, if the checklist is adapted rigorously, this shall be reflected in improved maternal and neonatal health outcomes in health facilities' patients and therefore, have a direct benefit for the respective patients. There shall be a decrease of birth complications and an increase of the delivery of essential childbirth practices and of the availability of stocks and supplies. Some studies have also shown a more efficient work organization within the health facility and better communication patterns among health personnel as an outcome of the checklist.

5.2 Potential benefits of the research to the local community and/or society

Research findings will be shared early on and on a regular basis with the health facility and personnel. If positive effects can be shown the leadership might be motivated to continue the checklist's use. This will then have an obvious benefit for the pregnant women and newborn in the local communities and their families. The number of post-natal check-ups might increase due to positive experiences of the women during the birth process and as the checklist reminds the health personnel to make an appointment with the mother. This might lead to improved long-term health outcomes of mother and child through, e.g., vaccination. The provincial and national government will also be informed regularly about the study findings and might accordingly decide to expand the instrument's use to other regencies, which would then lead to greater benefit for the Indonesian society. Improved birth outcomes at public health facilities as well as an increased delivery of essential health practices might also trigger the government to strengthen programs motivating pregnant women to give birth in health facilities instead of at home. Furthermore, families might be more interested and willing to attend the institutional health system, not only for births but also for other health issues.

6. Consent Process

6.1 Institutional Consent

The research project is carried out jointly by a team of the German Georg-August University and the Indonesian Syiah Kuala University within the framework of a Memorandum of Understanding between both universities. Furthermore, the study is supported by the local health minister Dr. Yani.

6.2 Health Institutions' Consent

As both roll-out and evaluation of the Safe Childbirth Checklist will be conducted jointly with Syiah Kuala University, we will cooperate with its teaching health facilities. For non-associated health facilities we will obtain written consent from the health facility leadership to implement the SCC in their institution and to conduct the survey within the study period. For the exact wording of the information document please refer to appendix C. We will obtain written consent from the health facility leadership of all participating health institutions to implement the SCC in their institution and to conduct the survey within the study period. For the exact wording of the information document please refer to appendix C.

6.3 Health Personnel Consent

As the instrument's adaptation lies within the scope of decision of the health facility leadership, we do not have to demand additional permission for the checklist's introduction from the health personnel. But before each questionnaire, we will obtain written consent to participate in the survey from the respective health workers. Interviewers will be carefully selected and trained not to exert any pressure on the health personnel to participate in the study. They will inform the health personnel that they are free to choose to drop out of the study at any point. The consent and the questions will be asked in a private room. For the exact wording of the information document please refer to appendix D.

6.4 Patient Consent

The health personnel will ask the patients consent to pass on their contact information to our research team. Out of those patients a randomized sample will be chosen for additional interviews. At the beginning of the interview, we will obtain written consent from the patients for the conduction of the survey. In case patients are illiterate we will ask for verbal consent. In case of unwritten consent, it will be signed by the person taking the consent and will be witnessed by a second person. For the exact wording of the information document please refer to appendix E. Interviewers will be carefully selected and trained not to exert any pressure on the patients to participate in the study. They will inform the patients that they are free to choose to drop out of the study at any point.

6.5 Delivery Skills Assessment Consent

To validate the outcomes of the treatment primary health facilities' personnel, written consent will be obtained for a "Delivery Skills Assessment", which might include a practical evaluation of every-day clinical work using a standardized OSCE (Objective Structured Clinical Examination) or similar format. Assessors will be carefully selected and trained not to exert any pressure on the health personnel to participate in the study. They will inform them that they are free to choose to drop out of the study at any point. The consent will be asked in a private room. For the exact wording of the information document please refer to appendix F.

7. Participant Privacy and Data Confidentiality

There are three major steps to protect participants' privacy interests. First, participants can decide not to enrol in the study. Second, if they enrol, interviews will take place in private rooms. Third we will make sure that all collected information is anonymous, meaning that all individual, household, village and health facility identifiers will be removed from the data, replaced by identification numbers and stored in a password-secured external drive. The individual and health facility data will be exclusively used to identify the health personnel and the facility for the follow-up surveys. All information collected on paper will be scanned and safely stored at the University of Goettingen in a password-secure external drive. Information on paper will be locked and destroyed after the completion of the study. Anonymized data will be stored on the local server of the University of Goettingen. It will be kept there for research reasons only. Anonymized data will be made publicly available after the study is finalized.

8. Statistical Analysis Plan

Random sampling and random assignment of Intervention Units to treatment and control group will allow us to interpret differences in mean outcomes as causal effects of the intervention. By comparing outcomes between the Intervention Units that adapted the instrument to those that did not, we will be able to draw conclusions of the intent-to-treat effect within the facilities and the size of the effect. Standard errors will be clustered at type of Intervention Unit (health facility levels).

$$Y_i = \alpha + \beta(T_i) + \delta X_{i,j} + \varepsilon_{ij}$$

Y is the respective health outcome (e.g. maternal or neonatal mortality rate or birth complication rate). T is a dummy, which indicates if the health facility was in the treatment group. i indexes the health facility, j indexes the patient. β measures the intent-to-treat effect. X encompasses different control variables at the SCC Intervention Unit and patient level. Hence, we will also test if effects differ by different intervention unit and by mean patient characteristics.

9. Research Staff

9.1 Main Investigation Team

Professor Dr. Sebastian Vollmer is the principal investigator of this project. He is Assistant Professor of Development Economics at the University of Goettingen and Adjunct Assistant Professor of Global Health at the Harvard T.H. Chan School of Public Health. He has extensively worked on issues related to health in low- and middle-income countries. Katharina Richert is a research associate and PhD candidate at Heidelberg University. Conducting her own research in Indonesia in the framework of the Postgraduate Training Programme of the German Development Institute Ms Richert acquired knowledge about the local context, which will be of large value for the underlying study. Lennart Kaplan is a PhD student and this project is part of his dissertation research. Mr Kaplan and Ms Richert will closely monitor and supervise the survey from Germany and Indonesia. Jana Kuhnt is a PhD student of Sebastian Vollmer and this project is part of her dissertation research. Ms. Kuhnt worked in several low- and middle-income countries. Ms. Kuhnt will support the analysis from Göttingen.

9.2 Extended Research Team

Farah Dibah is part of the research team from Syiah Kuala University. She holds a M.Sc. in Public Health and supports the data collection in Aceh province. Daria Anfilogova, a Master student in Development Economics from Georg-August University of Goettingen, will support the team in the baseline data collection and checklist implementation during July-September 2016.

9.3 Coordination Group

A 'Coordination Group' is currently being formed by Syiah Kuala University. The Coordination Group will provide support and guidance during the conceptualization and implementation of the WHO Safe Childbirth initiative in Indonesia.

More specifically, the duties and responsibilities of the group will be to:

- Provide technical advice and regular feedback to the research team
- Help with administrative tasks at Aceh province
- Establishing contacts with heads of health
- Recruit local enumerators
- Choose experts for SCC adaption (Technical Advisory Group)
- Monitoring/ Regular visits at health facilities between October 2016 and February 2017

9.4 Technical Advisory Group

A 'Technical Advisory Group' (TAG) is currently being formed, comprising local experts in the field of maternal and neonatal health. Participants come from various backgrounds, including midwives, obstetricians, gynaecologists, and also political actors. The TAG will be primarily responsible for adapting the checklist to the local context. The current WHO version of the checklist is only a pilot version that needs to be adapted to the local conditions, respecting the individual situations within the different health facilities. This adaptation process is an important step for this project to induce strong local ownership and hence, facilitate the usability of and the motivation for the checklist.

9.5 Field Investigators

Local medical staff will support the introduction and implementation of the instrument in the SCC Intervention Units. They will receive an intensive training and be supervised closely. Local field investigators will be trained on the protocol and questionnaire to conduct in the health facilities and among the patients. The field investigators will be closely monitored by the local staff of Syiah Kuala University and the University of Goettingen.

10. Reportable Events

None

11. Vulnerable Populations

The study precisely addresses public health facilities, serving a vulnerable group of pregnant women. The proposed intervention, the adaptation of the SCC, shall decrease adverse health outcomes for mother and newborn caused by the birth process.

12. Financing

Funding for the study comes from the Volkswagen Foundation.

13. Sharing Study Results

Results of the study will be published in peer-reviewed journals and as described in 5.2 will also be used to inform provincial and national government in order to provide them with concrete evidence required to determine whether to scale up the intervention to other regencies and provinces.

14. Research Collaboration

The "Evaluating the Impact of the WHO Safe Childbirth Checklist on the Quality of Care and Birth Outcomes in Health Facilities in Aceh Province" project will be conducted jointly between the Georg-August University of Goettingen, Germany and the Syiah Kuala University (UNSYIAH) in Banda Aceh, Indonesia. This project will profit from the long-standing relationship between both universities that facilitates an intensive cooperation and adds to the already existent exchange and collaboration. A strong network of former exchange students from UNSYIAH has been formed in Banda Aceh, which the project now can draw from.

Both partners will implement the project jointly in Aceh province. The University of Goettingen will ensure the financing of the study and take the lead in developing the study design, methodology, and will be responsible for data analysis. The Syiah Kuala University will support the local project implementation and its sustainable adaptation in the Aceh province. This includes gaining the political support and facilitating the dissemination of study results.

The joint project between the Local Government of Aceh Province (Ministry of Health) and the KfW "Reengineering and Improving the Hospital and Referral System in Selected Aceh" offers interesting opportunities for cooperation and might enable a scale-up of the study within the Aceh province. Alternatively, a potential cooperation with the WHO in the framework of its "Quality of Care" program might offer future opportunities for scaling-up the geographical checklist coverage.

15. Schedule

	2015			2016				2017	
	II	III	IV	I	II	III	IV	I	II
FFM Mission to choose local partners	■								
Get political support (national & district level	■	■	■	■					
Choose expert panel for SCC adaptation					■				
Develop instruments for data collection					■				
Adress HF and choose HFs that will participate in study					■				
Preparation of Workshop to adapt SCC					■	■			
Develop materials for SCC introduction					■	■			
Select and train enumerators					■	■			
Select treatment and control group of HFs						■			
Adapt WHO SCC to local context & translation						■			
Conduct data quality training						■			
Conduct baseline survey						■			
Implementation WHO SCC						■			
Endline data collection									■

II. Literature

Ekirapa-Kiracho, E., Waiswa, P., Rahman, M.H., Makumbi, F., Kiwanuka, N. (2011). Increasing access to institutional deliveries using demand and supply side incentives: early results from a quasi-experimental study. *BMC international health and human rights,* 11 Suppl 1: S11.

Lawn, J.E., Cousens, S., Zupan, J. (2005). "4 million neonatal deaths :When? Where? Why?". The Lancet, 365(9462): 891-900.

Lawn, J.E., Lee, A.C., Kinney, M., Sibley, L., Carlo, W.A., Paul, V.K., Darmstadt, G.L. (2009). Two million intrapartum-related stillbirths and neonatal deaths: where, why, and what can be done?. *International Journal of Gynecology & Obstetrics*, 107: 5-19.

Lim S.S., Dandona L., Hoisington J.A., James S.L., Hogan M.C., et al. (2010) India's Janani Suraksha Yojana, a conditional cash transfer programme to increase births in health facilities: an impact evaluation. *The Lancet,* 375: 2009–2023.

Liu, L., S. Oza, D. Hogan, J. Perin, I. Rudan, J. E. Lawn, S. Cousens, C. Mathers, R. E. Black (2014). Global, regional, and national causes of child mortality in 2000-13, with projections to inform post-2015 priorities: an updated systematic analysis. *The Lancet*, *385*(9966): 430-440.

National Research Council (2013). Reducing Maternal and Neonatal Mortality in Indonesia. Saving Lives, Saving the Future. Joint Committee on Reducing Maternal and Neonatal Mortality in Indonesia. Washington D.C.: The National Academies Press.

Powell-Jackson, T., Morrison, J., Tiwari, S., Neupane, B.D., Costello, A.M. (2009). The experiences of districts in implementing a national incentive programme to promote safe delivery in Nepal. *BMC health services research,* 9: 97.

Spector, Jonathan M., Angela Lashoher, Priya Agrawal, Claire Lemer, Gerald Dziekan, Rajiv Bahl, Matthews Mathai, Mario Merialdi, William Berry, Atul A. Gawande (2013). Designing the WHO Safe Childbirth Checklist program to improve quality of care at childbirth. *International Journal of Gynecology & Obstetrics*. 122(2): 164-168.

Spector, Jonathan M., Priya Agrawal, Bhalchandra S. Kodkany, Stuart Lipsitz, Angela Lashoher, Gerald Dziekan, Rajiv Bahl, Mario Merialdi, Matthews Mathai, Claire Lemer, Atul A. Gawande. (2012). Improving quality of care for maternal and newborn health: prospective pilot study of the WHO safe childbirth checklist program. *PLoS One*. 7(5): e35-151.

United Nations (2013). MDG 5: Improve maternal health: Fact Sheet. Available from http://www.un.org/millenniumgoals/news.shtml. [Accessed: 30.03.2014].

World Health Organization (2010). Countdown to 2015 decade report (2000–2010) with country profiles: taking stock of maternal, newborn and child survival. WHO, Geneva, Switzerland.

World Health Organization (2005). World Health Report: Make every mother and child count. WHO, Geneva, Switzerland.

III. Appendix

A. WHO Safe Childbirth Checklist (pilot version)
B. WHO Safe Childbirth Checklist Manual
C. Health Institution Consent Form
D. Health Personnel Consent Form
E. Patient Consent Form for Follow-Up Contact
F. Patient Consent Form
G. Delivery Skills Assessment Consent Form

Appendix A: WHO Safe Childbirth Checklist (pilot version)

Before Birth | SAFE CHILDBIRTH CHECKLIST - PILOT EDITION

World Health Organization

1. On admission

Does Mother need referral?
- ☐ No
- ☐ Yes, organized

Check your facility's criteria

Partograph started?
- ☐ No: Will start when ≥ 4 cm
- ☐ Yes

Start plotting when cervix ≥4 cm, then cervix should dilate ≥1 cm/hr
- Every 30 min: plot HR, contractions, fetal HR
- Every 2 hrs: plot temperature
- Every 4 hrs: plot BP

Does Mother need to start:

Antibiotics?
- ☐ No
- ☐ Yes, given

Give antibiotics to Mother if any of:
- Mother's temperature ≥38°C
- History of foul-smelling vaginal discharge
- Rupture of membranes >18 hrs

Magnesium sulfate?
- ☐ No
- ☐ Yes, given

Give magnesium sulfate to Mother if any of:
- Diastolic BP ≥110 mmHg and 3+ proteinuria
- Diastolic BP ≥90 mmHg, 2+ proteinuria, and any: severe headache, visual disturbance, epigastric pain

Antiretrovirals?
- ☐ No, confirmed HIV negative
- ☐ Yes, given
- ☐ If status unknown, HIV test ordered

- Mothers with CD4 ≤350 or clinical diagnosis require treatment
- Mothers with CD4 >350 require prophylaxis

☐ **Confirm supplies are available to clean hands and wear gloves for each vaginal exam**

☐ **Encourage Birth Companion to be present at birth**

☐ **Confirm that Mother or Companion will call for help during labour if needed**

Call for help if any of:
- Bleeding
- Severe abdominal pain
- Severe headache or visual disturbance
- Unable to urinate
- Urge to push

Completed by: ____________

2. Just before pushing (or before Caesarean)

Does Mother need to start:

Antibiotics?
- ☐ No
- ☐ Yes, given

Give antibiotics to Mother if any of:
- Mother's temperature ≥38°C
- History of foul-smelling vaginal discharge
- Rupture of membranes >18 hrs
- Caesarean section

Magnesium sulfate?
- ☐ No
- ☐ Yes, given

Give magnesium sulfate to Mother if any of:
- Diastolic BP ≥100 mmHg and 3+ proteinuria
- Diastolic BP ≥ 90 mmHg, 2+ proteinuria, and any: severe headache, visual disturbance, epigastric pain

Confirm essential supplies are at bedside and prepare for delivery:

for Mother
- ☐ Gloves
- ☐ Alcohol-based handrub or soap and clean water
- ☐ Oxytocin 10 units in syringe

Prepare to care for Mother immediately after birth:
Confirm single baby only (not multiple birth)
1. Give oxytocin within 1 minute after birth
2. Deliver placenta
3. Massage uterus after placenta is delivered
4. Confirm uterus is contracted

for Baby
- ☐ Clean towel
- ☐ Sterile blade to cut cord
- ☐ Suction device
- ☐ Bag-and-mask

Prepare to care for Baby immediately after birth:
1. Dry baby, keep warm
2. If not breathing, stimulate and clear airway
3. If still not breathing:
   – clamp and cut cord
   – clean airway if necessary
   – ventilate with bag-and-mask
   – shout for help

☐ **Assistant identified and ready to help at birth if needed?**

Completed by: ____________

This checklist is not intended to be comprehensive and should not replace the patient chart or partogragh. Additions and modifications to fit local practice are encouraged. For more information on recommended use of the checklist, please refer to the "Safe Childbirth Checklist Manual" at: www.who.int/patientsafety.

© WHO, 2012 WHO/IER/PSP/2012.15

After Birth | SAFE CHILDBIRTH CHECKLIST - PILOT EDITION

World Health Organization

3. Soon after birth (within 1 hour)

Is Mother bleeding abnormally?
- ☐ No
- ☐ Yes: Shout for help

If bleeding abnormally:
- Massage uterus
- Consider more uterotonic
- Start IV
- Treat cause: uterine atony, retained placenta/fragments, vaginal tear, uterine rupture

Does Mother need to start:

Antibiotics?
- ☐ No
- ☐ Yes, given

Give antibiotics to Mother if placenta manually removed or if Mother's temperature ≥38°C and any of:
- Chills
- Foul-smelling vaginal discharge

Magnesium sulfate?
- ☐ No
- ☐ Yes, given

Give magnesium sulfate to Mother if any of:
- Diastolic BP ≥110 mmHg and 3+ proteinuria
- Diastolic BP ≥90 mmHg, 2+ proteinuria, and any: severe headache, visual disturbance, epigastric pain

Does Baby need:

Referral?
- ☐ No
- ☐ Yes, given

Check your facility's criteria.

Antibiotics?
- ☐ No
- ☐ Yes, given

Give Baby antibiotics if antibiotics given to Mother, or if Baby has any of:
- Respiratory rate >60/min or <30/min
- Chest in-drawing, grunting, or convulsions
- Poor movement on stimulation
- Baby's temp <35°C (and not rising after warming), or Baby's temp ≥38°C

Special care/monitoring?
- ☐ No
- ☐ Yes, organized

Arrange special care/monitoring for Baby if any:
- More than 1 month early
- Birth weight <2500 grams
- Needs antibiotics
- Required resuscitation

Antiretrovirals?
- ☐ No
- ☐ Yes, organized

If Mother HIV+, follow local guidelines for Baby (prophylaxis to be started within 12 hours after birth)

☐ **Started breastfeeding and skin-to-skin contact** (if Mother and Baby well)

☐ **Confirm Mother/Companion will call for help if danger signs present**

Completed by: ____________

4. Before discharge

Is Mother's bleeding controlled?
- ☐ No: Treat and delay discharge
- ☐ Yes

Mother to start antibiotics?
- ☐ No
- ☐ Yes: Give and delay discharge

Give antibiotics to Mother if her temperature ≥38°C and any:
- Chills
- Foul-smelling vaginal discharge

Baby to start antibiotics?
- ☐ No
- ☐ Yes: Give antibiotics, delay discharge, give special care

Give antibiotics to Baby if any of:
- Respiratory rate >60/min or <30/min
- Chest in-drawing, grunting, convulsions
- Poor movement on stimulation
- Baby's temp <35°C (and not rising after warming), or temp ≥38°C
- Stopped breastfeeding well
- Umbilicus redness extending to skin or draining pus

Is Baby feeding well?
- ☐ No: Establish good breastfeeding practices and delay discharge
- ☐ Yes

If Mother HIV positive, Mother and Baby have ARVs for 6 weeks?
- ☐ Yes

☐ **Discuss and offer family planning options to Mother**

☐ **Arrange follow-up and confirm Mother/Companion will seek help if danger signs are present after discharge**

Completed by: ____________

DANGER SIGNS

Mother *has any of:*
- Bleeding
- Severe abdominal pain
- Severe headache or visual disturbance
- Breathing difficulty
- Fever or chills
- Difficulty emptying bladder

Baby *has any of:*
- Fast/difficult breathing
- Fever
- Unusually cold
- Stops feeding well
- Less activity than normal
- Whole body becomes yellow

Responsibility for the interpretation and use of the material in this checklist lies with the reader. In no event shall the World Health Organization be liable for damages arising from its use. This pilot edition is for research purposes and is under refinement by a WHO-led international collaborative. For more information visit www.who.int/patientsafety.

Appendix B: WHO Safe Childbirth Checklist Manual

Safe Childbirth Checklist Manual

Improving Health for Mothers and Newborns

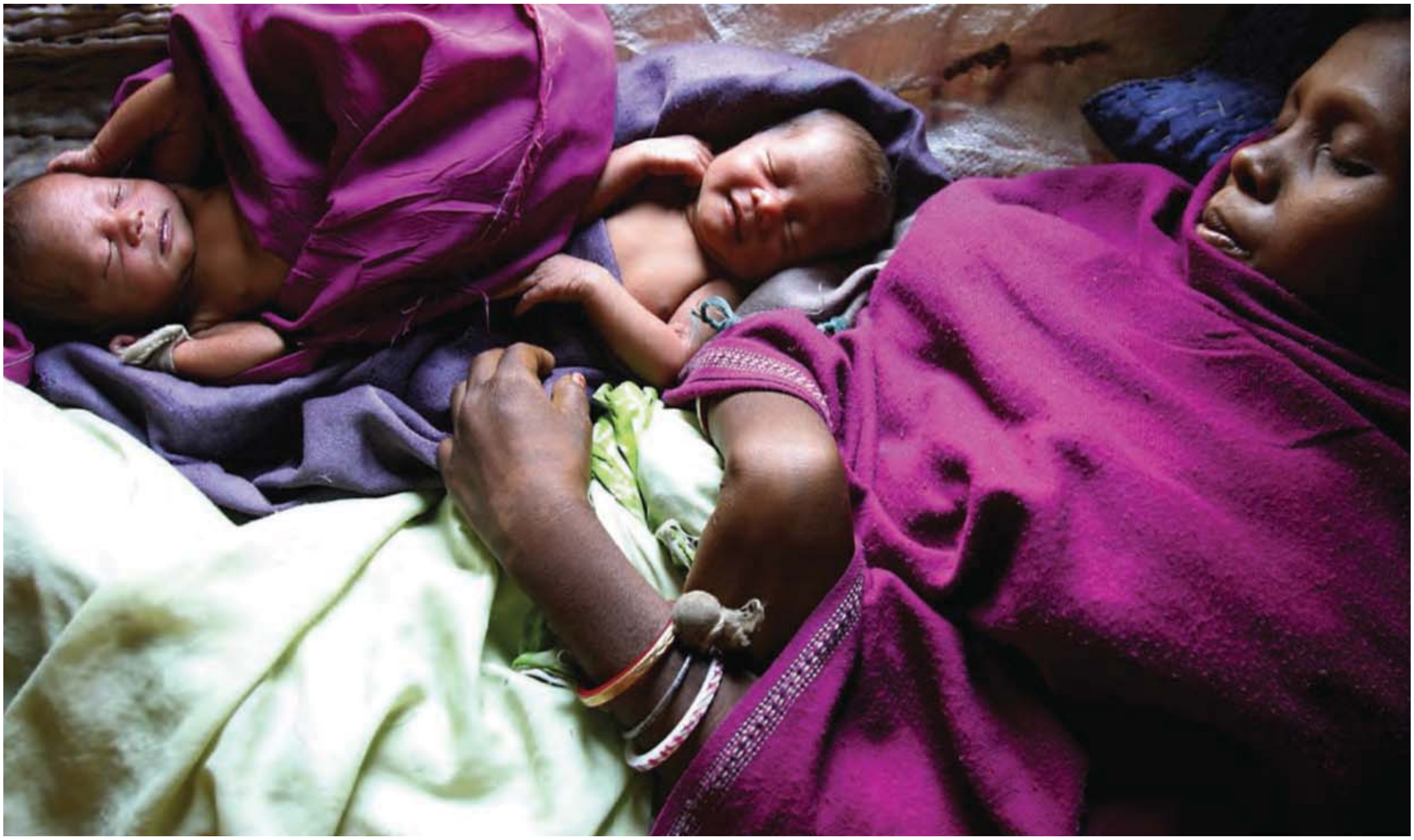

PILOT EDITION

World Health Organization

Safe Childbirth Checklist Manual

Improving Health for Mothers and Newborns

PILOT EDITION

THIS PILOT EDITION IS FOR RESEARCH PURPOSES

WHO Library Cataloguing-in-Publication Data

Safe childbirth checklist manual: improving health for mothers and newborns: pilot edition.

1. Parturition. 2. Maternal welfare.
3. Obstetric labor complications - prevention and control. 4. Infant, Newborn.
5. Patient care - standards. 6. Handbooks.
7. Developing countries. I.World Health Organization.

ISBN 978 92 4 150414 0 (NLM classification: WQ 300)

© World Health Organization 2012

All rights reserved. Publications of the World Health Organization are available on the WHO web site (www.who.int) or can be purchased from WHO Press, World Health Organization, 20 Avenue Appia, 1211 Geneva 27, Switzerland (tel.: +41 22 791 3264; fax: +41 22 791 4857; e-mail: bookorders@who.int). Requests for permission to reproduce or translate WHO publications – whether for sale or for noncommercial distribution – should be addressed to WHO Press through the WHO web site (http://www.who.int/about/licensing/copyright_form/en/index.html).
The designations employed and the presentation of the material in this publication do not imply the expression of any opinion whatsoever on the part of the World Health Organization concerning the legal status of any country, territory, city or area or of its authorities, or concerning the delimitation of its frontiers or boundaries. Dotted lines on maps represent approximate border lines for which there may not yet be full agreement.

The mention of specific companies or of certain manufacturers' products does not imply that they are endorsed or recommended by the World Health Organization in preference to others of a similar nature that are not mentioned. Errors and omissions excepted, the names of proprietary products are distinguished by initial capital letters.

All reasonable precautions have been taken by the World Health Organization to verify the information contained in this publication. However, the published material is being distributed without warranty of any kind, either expressed or implied. The Safe Childbirth Checklist – Pilot Edition presented in this document is not intended to be comprehensive and should not replace the patient chart or partograph. This pilot edition of the Checklist is for research purposes and is under refinement by a WHO-led international collaborative. The responsibility for the interpretation and use of the material lies with the reader. In no event shall the World Health Organization be liable for damages arising from its use.

Cover photograph reproduced with kind permission of UNICEF:
© UNICEF/NYHQ2005-2410/Anita Khemka

Designed by CommonSense, Greece

Printed by the WHO Document Production Services, Geneva, Switzerland

Table of Contents

About this manual 4

Introduction 5

How to use the Checklist 10

How to implement the Checklist 19

Acknowledgements 21

References 23

About this manual

This manual is a guide to support implementation of the Safe Childbirth Checklist programme. The primary target audience is health-care workers, administrators, and policy-makers that are working to improve childbirth care in institutional deliveries in low- and middle-income countries. The design is such that it can also be adapted for high-income countries.

The first part of this manual reviews the Safe Childbirth Checklist programme and makes available a reproducible copy of the checklist. The second part of this manual explains how to use the checklist, provides a detailed description of the checklist's four pause points and specific checklist items, and gives references for the evidence-based practices listed on the checklist. The last part of this manual reviews how to implement the checklist programme in institutions – since sustained adoption of checklist use by health-care workers is ultimately crucial to the programme's success.

This manual and the checklist are also available online at: www.who.int/patientsafety.

Introduction

Childbirth is a complex process. It can be difficult for health-care workers to remember to do everything that is needed to be sure the woman and baby receive the safest care possible. The Safe Childbirth Checklist is a simple tool to help health-care workers provide high quality care during institutional births – from the time the woman is admitted, through childbirth, until the woman and baby are safely discharged home.

Checklists prompt users to remember to complete essential tasks and have long been integral to maintaining safety in industries such as aviation. In recent years, checklists have also been found to improve safety in health. Trials of checklist programmes in intensive care medicine and surgery have demonstrated significant reductions in complications and deaths.[1,2]

Building on these successes, WHO – in consultation with nurses, midwives, obstetricians, paediatricians, patient safety experts and patients from around the world – developed the WHO Safe Childbirth Checklist programme to help health-care workers improve adherence to proven maternal and newborn care practices. Identifying effective and scalable methods to save lives at birth is a global priority to support progress towards Millennium Development Goals 4 and 5.

At the programme's core is the Safe Childbirth Checklist, a list of evidence-based practices derived from WHO guidelines that target the major global causes of maternal deaths (haemorrhage, infection, obstructed labour and hypertensive disorders), intrapartum-related stillbirths (inadequate intrapartum care) and neonatal deaths (intrapartum-related events, infection and complications of prematurity) - see Figure 1. Each checklist item is a critical action that, if missed, can lead to severe harm. Checklist additions and modifications to fit local practice are encouraged.

The Safe Childbirth Checklist (version 1.0) demonstrably improved health-care worker practice in pilot testing.[3] Large-scale evaluation of the programme's impact on outcomes is currently under way. Analysis of its effects on the daily operations of institutions and of its usability in various settings is encouraged by WHO. It is hoped that users of this checklist programme will contribute evidence of its effect by sharing their experience and lessons learned from implementation of the programme with WHO and the WHO Safe Childbirth Checklist Collaborative.[a]

---

[a] WHO has established the Safe Childbirth Collaborative to build synergies in learning about the adaptation, implementation and integration of the Safe Childbirth Checklist into clinical practice. To learn more about this knowledge sharing platform and to join it, but also to learn more about the ethical issues concerning maternal and newborn care, please visit: www.who.int/patientsafety.

THIS PILOT EDITION IS FOR RESEARCH PURPOSES

Figure 1: Safe Childbirth Checklist - Pilot Edition

Before Birth | SAFE CHILDBIRTH CHECKLIST - PILOT EDITION

1. On admission

Does Mother need referral?
- ☐ No
- ☐ Yes, organized

Check your facility's criteria

Partograph started?
- ☐ No: Will start when ≥ 4 cm
- ☐ Yes

Start plotting when cervix ≥4 cm, then cervix should dilate ≥1 cm/hr
- Every 30 min: plot HR, contractions, fetal HR
- Every 2 hrs: plot temperature
- Every 4 hrs: plot BP

Does Mother need to start:

Antibiotics?
- ☐ No
- ☐ Yes, given

Give antibiotics to Mother if any of:
- Mother's temperature ≥38°C
- History of foul-smelling vaginal discharge
- Rupture of membranes >18 hrs

Magnesium sulfate?
- ☐ No
- ☐ Yes, given

Give magnesium sulfate to Mother if any of:
- Diastolic BP ≥110 mmHg and 3+ proteinuria
- Diastolic BP ≥90 mmHg, 2+ proteinuria, and any: severe headache, visual disturbance, epigastric pain

Antiretrovirals?
- ☐ No, confirmed HIV negative
- ☐ Yes, given
- ☐ If status unknown, HIV test ordered

- Mothers with CD4 ≤350 or clinical diagnosis require treatment
- Mothers with CD4 >350 require prophylaxis

☐ **Confirm supplies are available to clean hands and wear gloves for each vaginal exam**

☐ **Encourage Birth Companion to be present at birth**

☐ **Confirm that Mother or Companion will call for help during labour if needed**

Call for help if any of:
- Bleeding
- Severe abdominal pain
- Severe headache or visual disturbance
- Unable to urinate
- Urge to push

Completed by: ____________

This checklist is not intended to be comprehensive and should not replace the patient chart or partogragh. Additions and modifications to fit local practice are encouraged. For more information on recommended use of the checklist, please refer to the "Safe Childbirth Checklist Manual" at: www.who.int/patientsafety.

World Health Organization

2. Just before pushing (or before Caesarean)

Does Mother need to start:

Antibiotics?

☐ No

☐ Yes, given

Give antibiotics to Mother if any of:
- Mother's temperature ≥38°C
- History of foul-smelling vaginal discharge
- Rupture of membranes > 18 hrs
- Caesarean section

Magnesium sulfate?

☐ No

☐ Yes, given

Give magnesium sulfate to Mother if any of:
- Diastolic BP ≥100 mmHg and 3+ proteinuria
- Diastolic BP ≥ 90 mmHg, 2+ proteinuria, and any: severe headache, visual disturbance, epigastric pain

Confirm essential supplies are at bedside and prepare for delivery:

for Mother

☐ Gloves

☐ Alcohol-based handrub or soap and clean water

☐ Oxytocin 10 units in syringe

Prepare to care for Mother immediately after birth:
Confirm single baby only (not multiple birth)
1. Give oxytocin within 1 minute after birth
2. Deliver placenta
3. Massage uterus after placenta is delivered
4. Confirm uterus is contracted

for Baby

☐ Clean towel

☐ Sterile blade to cut cord

☐ Suction device

☐ Bag-and-mask

Prepare to care for Baby immediately after birth:
1. Dry baby, keep warm
2. If not breathing, stimulate and clear airway
3. If still not breathing:
   – clamp and cut cord
   – clean airway if necessary
   – ventilate with bag-and-mask
   – shout for help

☐ Assistant identified and ready to help at birth if needed?

Completed by: ____________

THIS PILOT EDITION IS FOR RESEARCH PURPOSES

After Birth | SAFE CHILDBIRTH CHECKLIST - PILOT EDITION

3. Soon after birth (within 1 hour)

Is Mother bleeding abnormally?
- ☐ No
- ☐ Yes: Shout for help

If bleeding abnormally:
- Massage uterus
- Consider more uterotonic
- Start IV
- Treat cause: uterine atony, retained placenta/fragments, vaginal tear, uterine rupture

Does Mother need to start:

Antibiotics?
- ☐ No
- ☐ Yes, given

Give antibiotics to Mother if placenta manually removed or if Mother's temperature ≥38°C and any of:
- Chills
- Foul-smelling vaginal discharge

Magnesium sulfate?
- ☐ No
- ☐ Yes, given

Give magnesium sulfate to Mother if any of:
- Diastolic BP ≥110 mmHg and 3+ proteinuria
- Diastolic BP ≥90 mmHg, 2+ proteinuria, and any: severe headache, visual disturbance, epigastric pain

Does Baby need:

Referral?
- ☐ No
- ☐ Yes, given

Check your facility's criteria.

Antibiotics?
- ☐ No
- ☐ Yes, given

Give Baby antibiotics if antibiotics given to Mother, or if Baby has any of:
- Respiratory rate >60/min or <30/min
- Chest in-drawing, grunting, or convulsions
- Poor movement on stimulation
- Baby's temp <35°C (and not rising after warming), or Baby's temp ≥38°C

Special care/monitoring?
- ☐ No
- ☐ Yes, organized

Arrange special care/monitoring for Baby if any:
- More than 1 month early
- Birth weight <2500 grams
- Needs antibiotics
- Required resuscitation

Antiretrovirals?
- ☐ No
- ☐ Yes, organized

If Mother HIV+, follow local guidelines for Baby (prophylaxis to be started within 12 hours after birth)

☐ **Started breastfeeding and skin-to-skin contact** (if Mother and Baby well)

☐ **Confirm Mother/Companion will call for help if danger signs present**

Completed by: ____________

Responsibility for the interpretation and use of the material in this checklist lies with the reader. In no event shall the World Health Organization be liable for damages arising from its use. This pilot edition is for research purposes and is under refinement by a WHO-led international collaborative. For more information visit www.who.int/patientsafety.

World Health Organization

4. Before discharge

Is Mother's bleeding controlled?

☐ No: Treat and delay discharge
☐ Yes

Mother to start antibiotics?

☐ No
☐ Yes: Give and delay discharge

Give antibiotics to Mother if her temperature ≥38°C and any:
- Chills
- Foul-smelling vaginal discharge

Baby to start antibiotics?

☐ No
☐ Yes: Give antibiotics, delay discharge, give special care

Give antibiotics to Baby if any of:
- Respiratory rate >60/min or <30/min
- Chest in-drawing, grunting, convulsions
- Poor movement on stimulation
- Baby's temp <35°C (and not rising after warming), or temp ≥38°C
- Stopped breastfeeding well
- Umbilicus redness extending to skin or draining pus

Is Baby feeding well?

☐ No: Establish good breastfeeding practices and delay discharge
☐ Yes

If Mother HIV positive, Mother and Baby have ARVs for 6 weeks?

☐ Yes

☐ **Discuss and offer family planning options to Mother**

☐ **Arrange follow-up and confirm Mother/Companion will seek help if danger signs are present after discharge**

Completed by: ____________

DANGER SIGNS

***Mother** has any of:*
- Bleeding
- Severe abdominal pain
- Severe headache or visual disturbance
- Breathing difficulty
- Fever or chills
- Difficulty emptying bladder

***Baby** has any of:*
- Fast/difficult breathing
- Fever
- Unusually cold
- Stops feeding well
- Less activity than normal
- Whole body becomes yellow

THIS PILOT EDITION IS FOR RESEARCH PURPOSES

How to use the Checklist

How to use the Checklist

Childbirth is characterized by events that are both routine and unexpected. Complications can happen at any time. In order to provide the safest care possible for women and babies, health-care workers must deliver proven clinical practices continuously during the entire childbirth process, from the time the woman is admitted to the facility for childbirth, through delivery, and until the mother and baby are safely discharged home. The Safe Childbirth Checklist was designed to help health-care workers to ensure that high impact practices are performed at critical junctures during childbirth. The checklist is intended to be used at four points in time ("pause points"). At each pause point, a set of essential practices ("checklist items") should be completed.

One checklist should be used for every mother and her baby with each checklist item being marked with a pen when that item is completed. The health-care worker who is caring for the woman and baby at the time a pause point occurs should be responsible for completing the checklist at that point in time. Checklist users may be nurses, midwives, physicians, or other clinicians.

The recommended approach for using the checklist is for health-care workers to first conduct their normal practice and then use the checklist to verify that the checklist items have been correctly performed.

The four pause points

"Pause points" are specific points in time when health staff are asked to pause (i.e., temporarily stop) whatever else they are doing and check that essential clinical practices have been completed. Pause points happen at critical junctures in care when complications can be averted or adequately treated. They also take place at times when it is convenient for health-care workers to check the woman and baby.

The Safe Childbirth Checklist is intended for use at four pause points during institutional births:

1. On admission;
2. Just before pushing (or before Caesarean section);
3. Soon after birth (within 1 hour); and
4. Before discharge.

In many facilities, the pause points will not all occur in the same room. For instance, in some facilities pause point 1 will take place at the admission desk, pause point 2 will take place in the labour room, pause point 3 will take place in the postpartum bay, and pause point 4 will take place on the postpartum ward. In other facilities, in particular those that are small and have only a labour room, all pause points will occur in a single area. Determination of where health-care workers will conduct checks at each of the 4 pause points will need to be individually adapted to local settings. If the pause points take place in separate areas, then it is important that the checklist accompany the woman and baby when they are moved from room to room. In many situations this can be achieved by keeping the checklist with the woman's chart or medical record.

Checklist items

"Checklist items" are evidence-based practices that should be completed at each pause point. Items on the Safe Childbirth Checklist specifically address the major global causes of maternal and newborn deaths. Successful completion of checklist items by health-care workers will help keep the woman and baby safe.

The checklist items are not intended to be comprehensive; it would not be possible to list on a single checklist all practices that are required at

each birth. The checklist does, however, list a core set of practices that are proven to reduce maternal and newborn harm. The practices described in the checklist items should be conducted at each and every birth.

Supplemental information is provided on the checklist for many items in order to increase its usefulness. This information is located adjacent to the checklist item it describes. For instance, the supplemental information for the checklist item relating to the partograph describes how the partograph should be used, and the supplemental information for checklist items relating to medications describes the administration indications. Health-care workers should refer to the supplemental information as needed. After repeated use of the checklist, users may come to memorize the supplemental information. In this situation, users should still run the checklist by reviewing and marking each checklist item to be sure that all essential practices are conducted.

Several checklist items require administration of medications such as antibiotics, magnesium sulphate, antiretrovirals, and oxytocin. Specific antibiotics and antiretrovirals are not listed on the Safe Childbirth Checklist because different facilities may have access to different types of antibiotics and may follow different guidelines. Selection of antibiotics and antiretrovirals should be made according to WHO or local guidelines. Similarly, dosages and treatment courses for all medications should be aligned with WHO or local guidelines.

Each item on the Safe Childbirth Checklist is described in detail below.

Pause Point 1: On admission

Checking the mother at the time of admission is important to detect and treat complications that she may already have, to confirm whether she needs to be referred to another facility, to prepare her (and her companion) for labour and delivery, and to educate her (and her companion) about danger signs for which she should call for help.

Does Mother need referral?

Mothers with complications, or those at high risk of complications, may require referral to another facility to ensure they receive safe care. The checklist user should confirm whether the mother needs referral to another facility by reviewing the facility's criteria for referral. If indicated, the health-care worker should take immediate action to organize safe transfer. The health-care worker should communicate the reason for referral to the mother (and birth companion) and to health-care workers at the facility to which she is being referred. Posting a list of referral criteria in the admission area can serve as a useful reference for health-care workers and help them to rapidly identify mothers that should be referred.

Partograph started?

The partograph is a one-page tool used to assess labour progress. The alert and action lines on the partograph help health-care workers to recognize and take action to manage prolonged and obstructed labour. Studies have shown that use of the partograph can help to prevent prolonged labour, reduce operative intervention, and improve neonatal outcomes.[4] The checklist user should start the partograph when a mother's cervical dilation is 4 centimetres or more (i.e., when she is in active labour).[5,6] The mother's cervix should then dilate at a rate of at least 1 centimetre per hour. Every 30 minutes, the health-care worker should plot the mother's heart rate, contraction pattern, and the fetal heart rate on the partograph. Every 2 hours the mother's temperature should be plotted. Every 4 hours the mother's blood pressure should be plotted. If the mother is not in active labour at the time of admission, then a partograph should be attached to her chart or medical record and started when her cervical dilatation reaches 4 centimeters.

Additional information about the partograph is available at: http://www.who.int/maternal_child_adolescent/news_events/news/2010/distance_learning/en/ and http://whqlibdoc.who.int/hq/1993/WHO_ FHE_MSM_93.9.pdf.

THIS PILOT EDITION IS FOR RESEARCH PURPOSES

Does Mother need to start antibiotics?

Antibiotics prevent and treat bacterial infections. If a pregnant woman has an infection, or has risk factors for infection, then antibiotic treatment will help to prevent infection-related complications in her, in the fetus, and in the newborn.[7] The checklist user should confirm whether the mother needs antibiotics at the time of admission and, if indicated, the antibiotics should be immediately administered. Antibiotics should be administered if the mother has a temperature of 38°C or higher, foul-smelling vaginal discharge, or rupture of the membranes for more than 18 hours.[5,6]

Does Mother need to start magnesium sulphate?

Pre-eclampsia is a severe form of hypertension in pregnancy. Prophylactic treatment of mothers who have pre-eclampsia with magnesium sulphate will help to prevent hypertension-related complications in her (specifically, eclamptic fits or seizures), in the fetus, and in the newborn. The checklist user should confirm whether the mother needs magnesium sulphate at the time of admission and, if indicated, the magnesium sulphate should be urgently administered. Magnesium sulphate should be administered if the mother has diastolic blood pressure at or over 110 mmHg with 3+ proteinuria or if her diastolic blood pressure is at or over 90 mmHg with 2+ proteinuria and any signs of pre-eclampsia (severe headache, visual disturbance or epigastric pain).[5,8]

Does Mother need to start antiretrovirals?

Mothers who are positive for the Human Immunodeficiency Virus (HIV) can become very sick, and HIV can be passed from positive mothers to their babies. If a pregnant woman has HIV then lifelong antiretroviral treatment (ART) will help to prevent infection-related complications for her, the fetus, and for the newborn. It will also reduce the risk of transmission to her baby. Prophylaxis with antiretrovirals to mothers who are not eligible for ART will help to prevent virus transmission to newborns. The checklist user should confirm whether the mother needs antiretrovirals (ART or prophylaxis) at the time of admission and, if indicated, the antiretrovirals should be immediately administered.

HIV-positive mothers who have a CD4 cell count equal to or less than 350 cells/mm^3 or have a clinical diagnosis of the Acquired Immunodeficiency Syndrome (AIDS) should be started on lifelong ART.[9] If a pregnant woman is found to be HIV-positive and eligible for ART, then antiretroviral drugs should be given according to national protocol. HIV-positive mothers who need ART should continue treatment throughout labour, birth, breastfeeding, and thereafter.

Prophylactic doses of antiretrovirals should be administered to HIV-positive mothers who have a CD4 cell count greater than 350 cells/mm^3 and no clinical diagnosis of AIDS. HIV-positive mothers that need prophylaxis should continue prophylaxis throughout labour, birth and breastfeeding.

In countries recommending ART for all HIV-positive pregnant women irrespective of CD4 count or clinical staging, treatment should start as soon as possible after HIV status is confirmed and appropriate counselling and explanation.

If the mother's HIV status is unknown at the time of admission, then an HIV test should be immediately obtained if possible according to locally recommended practices. Every mother's HIV status should be documented in the medical record. It is important that other health-care workers who care for the mother and baby know about the mother's HIV status so that appropriate management of the mother and baby after birth can be assured.*

Confirm supplies are available near bedside to clean hands and wear gloves for each vaginal exam

Health-care workers with unclean hands can transmit infections to mothers and babies. Good hand hygiene practices help to prevent avoidable infections. Health-care workers should thus use an alcohol-based hand rub, or thoroughly wash their hands with soap and clean water every time before and after they have contact with a mother or newborn. Any time a health-care worker has contact with secretions from a mother or newborn (for example, during vaginal exams) then health-care workers should thoroughly wash their hands and also wear clean gloves.[5,6,10] Health-care workers should also clean their hands before any clean aseptic procedure.

Hygiene supplies (i.e. soap and clean running water or alcohol-based hand rub, and clean gloves) must be readily available and accessible at all times to help health-care workers to adhere to good hand hygiene practices.

Correct techniques for hand hygiene using alcohol-based hand rub or soap and clean water are available at: http://www.who.int/gpsc/5may/tools/workplace_reminders/en/

* Concerning ethical implications related to HIV testing, please refer to the website www.who.int/patientsafety.

Encourage Birth Companion to be present at birth

Birth companions provide support to the mother during labour, childbirth, and postpartum. They can also help to recognize danger signs, alert the health-care worker in the case of an emergency, and care for the baby. Examples of possible birth companions are family members, spouses, friends, community health workers, doulas, or staff members.

Evidence shows that birth companions can help to improve health outcomes. The presence of birth companions increases the likelihood that the mother will have a spontaneous vaginal delivery instead of a caesarean, vacuum, or forceps birth.[10] Mothers with birth companions have also been shown to need fewer pain medications, be better satisfied with their delivery experience, and have slightly shorter labours. Babies can also benefit. Studies have shown that newborns' 5-minute Apgar Scores are better and there is improved maternal bonding postnatally when birth companions are present.[5,6,10,11]

The checklist user should encourage the presence of a birth companion during labour, birth, and the postpartum and postnatal periods. If a birth companion is present at the time of admission then the birth companion should be encouraged to stay through the entire childbirth process. If a birth companion is not present at the time of admission, then the mother should be encouraged to identify a birth companion if possible.

Confirm that Mother or Companion will call for help during labour if needed

Complications are unpredictable and can happen at any time during childbirth. In general, complications become more difficult to manage the longer they go undetected and untreated. It is therefore important for health-care workers to detect and treat complications as soon as possible.

"Danger signs" are clinical signs and symptoms that indicate a complication may be developing or is already present. Many times health-care workers will recognize danger signs directly. Sometimes, however, health-care workers will be attending to other delivery cases or will be otherwise distracted at the time that a danger sign develops in a mother or baby. In such situations, it is important that the mother (and birth companion) alert health-care workers to the presence of danger signs. Mothers (and birth companions) should therefore be educated to recognize danger signs and to alert a health-care worker immediately in the event that a danger sign occurs. Health-care workers are encouraged to share their own names with the mother and birth companion since this usually helps the mother and the birth companion to feel more comfortable asking for help.

The checklist user should tell the mother (and birth companion) at the time of admission to alert a health-care worker immediately if any of the following danger signs develop during labour: bleeding, severe abdominal pain, severe headache or visual disturbance, or inability to urinate. The checklist user should also tell the mother to alert a health-care worker when she feels the urge to push since this means the baby will likely be born soon.

Pause Point 2: Just before pushing (or before Caesarean)

Checking the mother just before pushing (or before Caesarean) is important to detect and treat complications that can occur during labour and to prepare for routine events and possible crisis situations that may occur after birth.

Does Mother need to start antibiotics?

As described above, antibiotics prevent and treat bacterial infections. If a labouring mother has an infection, or has risk factors for infection, then antibiotic treatment will help to prevent infection-related complications in her, in the fetus, and in the newborn.[7] The checklist user should confirm whether the mother needs antibiotics at the time that pushing starts and, if indicated, the antibiotics should be immediately administered. Antibiotics should be administered if the mother has a temperature of 38°C or higher, foul-smelling vaginal discharge, or rupture of the membranes for more than 18 hours.[5,6] Antibiotics should also be administered if the mother will be undergoing a Caesarean section delivery.[7]

Does Mother need to start magnesium sulphate?

As described above, pre-eclampsia is a severe form of hypertension in pregnancy. Prophylactic treatment of mothers who have pre-eclampsia with magnesium sulphate will help to prevent hypertension-related complications in her (specifically, eclamptic fits or seizures), in the fetus, and in the newborn. The checklist user should confirm whether the mother needs magnesium sulphate at the time that pushing starts and, if indicated, the magnesium sulphate should be immediately administered. Magnesium sulphate

THIS PILOT EDITION IS FOR RESEARCH PURPOSES

should be administered if the mother has diastolic blood pressure at or over 110 mmHg with 3+ proteinuria or if her diastolic blood pressure is at or over 90 mmHg with 2+ proteinuria and any signs of pre-eclampsia (severe headache, visual disturbance or epigastric pain).[5,8]

Confirm essential supplies are at bedside and prepare for delivery

The moment of birth and the first few minutes after birth are the highest risk periods for complications in the mother and the baby. Crisis situations can evolve quickly and put the mother and baby at very high risk of complications or death. In general, health-care workers will not have enough time to prepare once a crisis situation has started. Health-care workers must therefore prepare beforehand for both routine care and potential crisis situations *at every birth* in order to keep the mother and baby safe.

There are two ways in which health-care workers must be prepared at the time of birth. Specifically, health-care workers must prepare essential supplies and also prepare themselves to take essential actions. The essential supplies must always be clean, functioning, and ready to use before the birth occurs. Actions must be performed immediately or complications can develop. Health-care workers must therefore remember the essential actions *before* birth–so that they can quickly complete them at the time of birth and in the first few minutes after birth. More information about care that should be provided to the mother and baby at the time of birth can be found at:
http://whqlibdoc.who.int/publications/2006/924159084X_eng.pdf

For Mother:

At the start of pushing (or before Caesarean), health-care workers should confirm that the following essential supplies for the mother are at the bedside and ready to be used at the time of birth: gloves; soap and clean water with single-use towels, or alcohol-based hand rub; and oxytocin (10 international units in a syringe).

The use of gloves, soap and clean water, single-use towels and alcohol-based hand rub is to ensure good hand hygiene practices during delivery to prevent infection in the mother and baby. The use of oxytocin is to help the uterus to contract to prevent postpartum bleeding. At the start of pushing health-care workers should also review the steps involved to care for the mother immediately after birth. Essential actions for the mother immediately after birth will help to ensure safe expulsion of the placenta and prevent postpartum bleeding. The first step is to be sure there are no additional babies to be delivered. The second step is to administer 10 IU of oxytocin intramuscularly to the mother within 1 minute of delivery. If oxytocin is not available, alternative medicines may be used (a list of alternatives is available at:
http://whqlibdoc.who.int/publications/2009/9789241598514_eng.pdf). The third step is to clamp and cut the cord before ensuring complete delivery of the placenta. The fourth step is to massage the uterus immediately after the delivery of placenta. This technique helps the uterus to contract and will help to prevent bleeding. Finally, the health-care worker should feel the uterus to be sure that it remains contracted.

For Baby

At the start of pushing (or before Caesarean), health-care workers should confirm that the following essential supplies for the baby are at the bedside and ready to be used at the time of birth: clean towel, sterile blade to cut cord, suction device, and bag-and-mask.

The use of a clean towel to dry the baby immediately after birth will help to keep the baby warm since amniotic and vaginal fluid on the baby can promote potentially harmful cooling as the fluid evaporates.[5,12] Also, the process of drying the baby provides stimulation for the baby that will help to signal the baby to cry or breathe.

The use of a sterile blade to cut the cord will help to prevent infection in the newborn baby (unsterile blades risk transmitting infection to the baby).[5,12] A tie or cord clamp should be placed around the cord before cutting in order to prevent bleeding.[5,12] Evidence suggests that the best time to clamp and cut the cord is 1-3 minutes after the baby is born.[13] This length of time allows the right amount blood to enter the baby's circulation.

The use of a suction device to clear secretions from the baby's mouth and nose will be important if the baby's airway is obstructed and the baby fails to immediately cry or breathe at birth. The use of a bag-and-mask device will be important if the baby requires resuscitation to begin crying or breathing.[13]

At the start of pushing health-care workers should also review the steps involved to care for the baby immediately after birth. Essential actions for

the baby immediately after birth will help to ensure a successful transition to extrauterine life. The period of 1 minute following birth is called the "golden minute" for the baby because the baby must start crying or breathing by approximately 1 minute of age in order to be safe.[13] If the baby does not cry or breathe spontaneously within 1 minute of birth, then health-care workers must quickly give assistance.

The first step after birth for all babies is to immediately dry and keep the baby warm. Keeping the baby warm can be accomplished by putting the baby skin-to-skin on the mother or covering the baby with a warm, dry cloth. If the baby cries and appears healthy, then routine care can be provided. If the baby does not cry or breathe, then the health-care worker should stimulate the baby by rubbing the baby's back. If the baby still does not cry or breathe, then the health-care worker should quickly clamp and cut the umbilical cord, clean or suction the baby's mouth and nose if they are obstructed, urgently ventilate the baby with a bag-and-mask, and call for help.

Most babies that do not cry or breathe at birth will begin to do so when they are stimulated. Babies that do not respond to stimulation will almost always start to cry or breathe when positive pressure ventilation is appropriately delivered with a bag-and-mask.[5, 12, 13, 14]

Assistant identified and informed to be ready to help at birth if needed?

As described above, the moment of birth and the first few minutes after birth are the highest risk period for complications in the mother and the baby and preparation is paramount. Health-care workers must prepare beforehand for possible crisis situations at birth in order to keep the mother and baby safe. Having an assistant available in the event that a crisis situation occurs is also important. They can perform several complimentary roles including assessing the mother or baby, starting IVs, administering medications, organizing referrals, and calling for additional help.

At the start of pushing, health-care workers should identify an assistant who is informed that the birth will happen soon, who will stay nearby, and be ready to help at birth if needed. The assistant can be another health-care worker or, in settings where there are staff shortages, the assistant can be the birth companion or another layperson (in this case, the assistant will not be expected to perform medical tasks such as starting IVs or administering medications, but can help with gathering supplies, calling for additional help, and other tasks).[5,8]

Pause Point 3: Soon After Birth (Within 1 Hour)

Checking the mother and baby soon after birth (within 1 hour) is important to detect and treat complications that can happen after delivery, and to educate the mother (and her companion) about danger signs for which she should call for help.

Is Mother bleeding abnormally?

Abnormal postpartum bleeding is a major complication that must be detected and treated early. Postpartum bleeding can occur because of several different conditions including uterine atony, retained placenta or placental fragments, a vaginal tear, or uterine rupture. Abnormal bleeding is defined by a blood loss of 500 ml or more, or any blood loss in which the mother's condition deteriorates, particularly if she is anaemic (if a mother is severely anaemic, the threshold for initiating action may be much lower than 500 ml).[15, 16]

The checklist user should assess the mother for abnormal bleeding soon after birth (within 1 hour) and perform the following actions if the mother is bleeding abnormally: massage the uterus, consider the administration of more uterotonic such as oxytocin, start an IV and give IV fluids, and treat the specific cause of the abnormal bleeding.[8, 11, 13, 15, 17]
Additional information about managing postpartum haemorrhage is available at: http://whqlibdoc.who.int/publications/2009/9789241598514_eng.pdf.

Does Mother need to start antibiotics?

As described above, antibiotics prevent and treat bacterial infections. If a mother in the postpartum period has an infection, or has risk factors for infection, then antibiotic treatment will help to prevent infection-related complications.[7]
The checklist user should confirm whether the mother needs antibiotics soon after birth (within 1 hour) and, if indicated, the antibiotics should be immediately administered. Antibiotics should be administered if the mother's placenta was manually removed, or if she has a temperature of 38°C or higher and chills or foul-smelling vaginal discharge.[5, 6, 11, 16]

THIS PILOT EDITION IS FOR RESEARCH PURPOSES

Does Mother need to start magnesium sulphate?

As described above, pre-eclampsia is a severe form of hypertension in pregnancy. Prophylactic treatment of mothers who have pre-eclampsia with magnesium sulphate will help to prevent hypertension-related complications (specifically, eclamptic fits or seizures). Hypertensive disease in pregnancy can still be a problem after delivery of the baby; up to a third of eclamptic fits occur after childbirth. The checklist user should confirm whether the mother needs magnesium sulphate soon after birth (within 1 hour) and, if indicated, the magnesium sulphate should be immediately administered. Magnesium sulphate should be administered if the mother has diastolic blood pressure at or over 110 mmHg with 3+ proteinuria or if her diastolic blood pressure is at or over 90 mmHg with 2+ proteinuria and any signs of pre-eclampsia (severe headache, visual disturbance or epigastric pain).[5,8]

Does baby need referral?

Babies with complications may require referral to another facility to ensure they receive safe care.[5,12,15,17] The checklist user should confirm whether the baby needs referral to another facility by reviewing the facility's criteria for referral. If referral is indicated, the health-care worker should take immediate action to organize safe transfer. The health-care worker should communicate the reason for referral to the mother and to health-care workers at the facility to which the baby is being referred. Posting a list of referral criteria in the postnatal area can serve as a useful reference for health-care workers and help them to rapidly identify babies that should be referred.

Does Baby need antibiotics?

As described above, antibiotics prevent and treat bacterial infections. If a baby has an infection, or has risk factors for infection, then antibiotic treatment will help to prevent infection-related complications.[7] Babies are particularly susceptible to infections because their immune systems are relatively weak. It is essential that babies with an infection or risk factors for an infection be treated immediately. The checklist user should confirm whether the baby needs antibiotics soon after birth (within 1 hour) and, if indicated, the antibiotics should be immediately administered. The baby needs antibiotics if antibiotics were administered to the mother, or if the baby has any of the following: respiratory rate > 60 per minute or < 30 per minute; chest in-drawing, grunting, or convulsions; poor movement on stimulation; or temperature < 35°C (and not rising after warming) or temperature ≥ 38°C.[5,11,18]

Does Baby need special care or monitoring?

Some babies may have risk factors that do not meet criteria for referral, but for which special care or monitoring is required to be sure that the baby stays safe. For example, small or premature babies may appear healthy, but they are in fact much more susceptible to complications in the first hours and days after birth. Checklist users should confirm whether the baby needs special care or monitoring soon after birth (within 1 hour) and, if indicated, the special care or monitoring should be immediately arranged. Special care or monitoring should be given if the baby is born more than 1 month early, has a birth weight < 2500 grams, needs antibiotics, or required resuscitation to help cry or breathe at birth.[5,13,17,19,20]

Does Baby need antiretrovirals?

HIV infection can be transmitted to babies from mothers who are HIV-positive. Administering antiretroviral prophylaxis to babies immediately after birth and throughout the breastfeeding period can help to decrease the risk of HIV transmission.[9,21] Checklist users should confirm before delivery whether the baby needs antiretroviral prophylaxis and, if indicated, antiretroviral prophylaxis should be given as soon after birth as possible (within 4-6 hours). Thereafter, if the mother is HIV-positive, antiretroviral prophylaxis should be administered according to local guidelines.

Start breastfeeding and skin-to-skin contact (if mother and baby are well)

Early breastfeeding is good for both babies and mothers. Evidence suggests that early breastfeeding within 1 hour of birth helps the baby to establish good bonding with the mother. Early breastfeeding may also stimulate uterine contraction for the mother through maternal hormone release and help to prevent postpartum vaginal bleeding.[18,22,23]

Babies are highly susceptible to cold stress. Complications can happen quickly if a baby's core temperature falls below the normal range. Skin-to-skin contact of the baby with the mother is the best method for keeping the baby warm. To give skin-to-skin contact, the baby's skin should be placed against the mother's skin, and then a clean sheet or blanket should be wrapped around the mother and the baby together. Immediate skin-to-skin contact after delivery also helps to promote bonding between the baby and the mother.[5,12,13,17,18,20]

If the mother and baby are well, the checklist user should confirm that breastfeeding and skin-to-skin contact has been started soon after birth (within 1 hour).

Confirm Mother/Companion will call for help if danger signs present

As described above, complications are unpredictable and can happen at any time during the childbirth process. This is true for both mothers and babies. Mothers (and birth companions) should be educated to recognize danger signs and to alert a health-care worker immediately in the event that a danger sign occurs.

The checklist user should tell the mother (and birth companion) soon after birth (within 1 hour) to alert a health-care worker immediately if any of the following danger signs for the mother develop in the postpartum period: bleeding, severe abdominal pain, severe headache or visual disturbance, difficulty breathing, fever or chills, or difficulty emptying bladder.[5, 12, 13, 17, 18]

The checklist user should also tell the mother (and birth companion) soon after birth (within 1 hour) to alert a health-care worker immediately if any of the following danger signs for the baby develop in the postnatal period: fast breathing or difficulty breathing, fever, unusually cold, stops feeding well, less activity than normal, or whole body becomes yellow.[5, 12, 13, 17, 18]

Pause Point 4: Before Discharge

Checking the mother and baby before discharge is important to be sure that the mother and baby are healthy before discharge, that follow-up has been arranged, that family planning options have been discussed and offered, and to educate the mother (and her companion) about danger signs after discharge for which immediate skilled care is needed.

Is Mother's bleeding controlled?

As described above, abnormal postpartum bleeding is a major complication that must be detected and treated early. The checklist user should confirm whether the mother's bleeding is controlled before discharge. This can be accomplished by asking the mother about her blood loss and by examining the mother. The health-care worker should examine the mother's abdomen to be sure that the uterus is contracted and check blood loss from the vagina.[15] If the mother's bleeding is not controlled, then the mother should be treated and the mother's discharge should be delayed. Under no circumstances should the mother be discharged home with uncontrolled bleeding.

Does Mother need to start antibiotics?

Antibiotics are needed to treat infections that develop in the mother in the postpartum period. Puerperal sepsis is a major cause of maternal infection after delivery. Other potential infections are mastitis or wound infection after a Caesarean section. The checklist user should confirm whether the mother needs antibiotics before discharge and, if indicated, the antibiotics should be immediately administered and discharge should be delayed. Antibiotics should be administered and discharge delayed if the mother has a temperature of 38°C or higher and chills or foul-smelling vaginal discharge.[5, 6, 11, 16]

Does Baby need to start antibiotics?

Antibiotics are needed to treat infections that develop in the baby in the postnatal period. Bacterial sepsis is a major cause of death in newborn babies.[23] The checklist user should confirm whether the baby needs antibiotics before discharge and, if indicated, the antibiotics should be immediately administered, discharge should be delayed, and special care or monitoring should be given. The baby needs antibiotics if any of the following are present: respiratory rate > 60 per minute or < 30 per minute; chest in-drawing, grunting, or convulsions; poor movement on stimulation; temperature < 35°C (and not rising after warming) or temperature ≥ 38°C; stopped breastfeeding well; or umbilicus redness extending to skin or draining pus.[5, 12, 18]

Is Baby feeding well?

The checklist user should confirm that adequate breastfeeding has been established before the mother and baby are discharged from the birth facility. In the event that breastfeeding is not possible, then the checklist user should confirm that the baby is bottle feeding adequately. Signs of feeding well in the baby are active feeding every 1-3 hours with frequent urination or stooling.[5, 13, 17, 18] If the baby is not feeding well, then help should be given to the mother and the baby to establish good feeding and the discharge should be delayed.

If mother is HIV positive, do mother and baby have ARVs for 6 weeks?

The checklist user should have confirmed by now whether the mother is HIV-positive and whether treatment or prophylaxis with antiretrovirals are indicated according to local guidelines. If the mother is HIV-positive, a six week supply of antiretrovirals should be given to the mother

THIS PILOT EDITION IS FOR RESEARCH PURPOSES

and baby and follow-up for continued HIV management should be arranged.[6]

Discuss and offer family planning options to Mother

Family planning can help to prevent unwanted pregnancies and help to keep the mother safe in the future. Checklist users should confirm that family planning options have been discussed with and offered to the mother before discharge. Ideally, mothers should be given at least two family planning options. Family planning options may include condoms, intra-uterine devices, long-acting injectable progesterone (DMPA), oral contraceptives, and tubal ligation.

Intra-uterine devices can be inserted immediately after childbirth or after six weeks postpartum. Insertion of an intra-uterine device within 10 minutes of placenta delivery is best, but it can also be inserted up to 48 hours postpartum with low levels of expulsion.[25]

Use of progestogen-only methods, with the exception of the levonorgestrel-bearing intra-uterine device, are not usually recommended for mothers who are less than 6 weeks postpartum and breastfeeding, unless other more appropriate methods are unavailable or unacceptable. Beyond 6 weeks postpartum, there is no restriction for the use of progestogen only contraceptive methods among breastfeeding mothers. The levonorgestrel-bearing intra-uterine device is not usually recommended for the first 4 postpartum weeks, unless other more appropriate methods are unavailable or unacceptable. Beyond 4 weeks postpartum, there is no restriction on its use.[26]

Use of combined hormonal contraceptives depends upon whether or not the mother is breastfeeding or not. For mothers who are not breastfeeding, use of combined hormonal contraceptives containing estrogen should generally be avoided for the first 21 days postpartum. In addition, mothers who are not breastfeeding and have additional risk factors for venous thromboembolism should not use them in the first 21 days postpartum.[27] Between 21 and 42 days postpartum, combined hormonal contraceptives can generally be used by mothers who are not breastfeeding, although for some of these mothers with additional risk factors for thromboembolism, these methods should not be used unless other more appropriate methods are not available.

Combined hormonal contraceptives are not recommended for women who are breastfeeding during the first six weeks postpartum, and this method should generally not be used by breastfeeding mothers prior to six months postpartum. Additional information on medical eligibility criteria for contraceptive use is available at http://whqlibdoc.who.int/publications/2010/9789241563888_eng.pdf.

If the mother wishes to have tubal ligation, it may be advantageous to schedule this procedure before she is discharged.

Women should be allowed to decide freely regarding the family planning options that best suit them, but should be encouraged to consult with relatives if they wish to.

Checklist users should also take the opportunity before discharge to discuss with the mother optimal birth spacing. After a live birth, the recommended interval before attempting the next pregnancy is at least 24 months in order to reduce the risk of adverse maternal, perinatal and infant outcomes.[5,12,28]

Arrange follow-up and confirm Mother/Companion will seek help if danger signs are present after discharge

Even if the mother and baby appear healthy at the time of discharge, complications can occur after the mother and baby are discharged home. Routine follow-up for both the mother and baby is necessary so that health-care workers can detect and treat complications early.

Mothers (and birth companions) should also be educated to recognize danger signs themselves for which skilled care should be sought after discharge.

The checklist user should tell the mother (and birth companion) before discharge to alert a health-care worker immediately if any of the following danger signs occur in the mother: bleeding, severe abdominal pain, severe headache or visual disturbance, difficulty breathing, fever or chills, or difficulty emptying bladder.[5,12,13,17,18]

The checklist user should tell the mother (and birth companion) before discharge to alert a health-care worker immediately if any of the following danger signs occur in the baby: fast breathing or difficulty breathing, fever, unusually cold, stops feeding well, less activity than normal, or whole body becomes yellow.[12,13,17,18]

How to implement the Checklist

The way in which the Safe Childbirth Checklist is introduced to health-care workers is important. Sustained adoption will only be achieved if health-care workers genuinely appreciate that the programme can help them to provide safer care for mothers and babies.

The pilot test of the Safe Childbirth Checklist successfully incorporated an implementation method based on a well-described model for behaviour change in health-care settings. This model is characterized by engagement with hospital administration and staff, review of baseline deficiencies in care, quality improvement and checklist training, supervised use of the checklist, and ongoing monitoring and feedback.[29-31]

Specific implementation packages should be designed and adapted to maximize cultural relevance and appropriateness. In general, checklist implementation programmes are more likely to be successful if the following components are integrated:

Engagement

Local ownership of the programme is essential. Implementers are encouraged to engage staff at all levels of the facility's health-care system early on when establishing the programme. This includes administrators, clinical leaders, health-care workers and patients. Discussing how the checklist is expected to improve patient safety and quality of care is an important first step.[31]

Where possible, giving all health-care workers who are involved with childbirth activities the opportunity to fully learn about the programme before it is implemented will help to promote successful uptake.

As a way of demonstrating institutional engagement with the checklist programme, it will be especially helpful if facility leaders publicly embrace the programme. These leaders will send a powerful message of support for the programme to the rest of the staff if they use the checklist themselves and monitor progress of the programme's implementation.[32]

Some facilities may select one or more members of the local staff to be "implementation leads" or "project champions" that take an active role in guiding each step of the programme's rollout.

Review of current deficiencies

Knowledge of deficiencies in practice can be a powerful motivator for health-care workers to change their behaviour for the better. If possible, implementers should work with local facility leaders to provide health-care workers with baseline data that highlight performance gaps in current clinical practices and outcomes (it is important that this be done in a non-threatening manner). Being clear about deficiencies that exist before the checklist programme is rolled out can also help to provide benchmarks against which to measure improvement after the programme is implemented – the ability to show improvement as the programme moves forward is a powerful tool for engagement and sustainability.

Checklist modification

Implementers are encouraged to work with local facility leaders and staff to modify the checklist as needed to suit the local setting. The Safe Childbirth Checklist was developed according to WHO guidelines and international standards of care. For this reason, significant modification of the checklist may hamper its efficacy. However, minor modification of the checklist may be required to ensure its consistency with local practice, guidelines, and culture. The recommended process by which to modify the checklist is as follows:

1. Evaluate whether modifications are needed

THIS PILOT EDITION IS FOR RESEARCH PURPOSES

2. Prioritize potential modifications
3. Assess the impact of potential modifications on usability
4. Modify the actual checklist

Common ways in which the checklist might be modified are addition of the facility's specific criteria for referral of the mother or baby, and adapting indications for medication administration (i.e., antibiotics, magnesium sulphate, and antiretrovirals) according to local guidelines. The process of checklist modification can also contribute to increased local ownership over the programme, which will help lead to sustained adoption of the checklist.

Training and launch

Implementers should work with facility leaders to provide training that covers all aspects of the Safe Childbirth Checklist, including the pause points, checklist items, and supplemental information. Training may incorporate materials such as handouts, lectures, and/or an instructional video. Hands-on simulation is generally considered to be an essential component of training.

Introduction of the checklist at an official launch event attended by all health facility personnel involved in childbirth activities can serve important purposes. Such an event can further engage the facility staff in the programme, provide the opportunity for additional education regarding why health-care workers' participation is essential, provide a forum in which potential barriers and enablers of the programme can be addressed, and generally create an atmosphere of excitement around this new quality improvement programme that can help to improve health for mothers and babies.

In the days following the launch event, implementers and/or selected facility staff are encouraged to be readily available to help answer questions or troubleshoot problems that arise when the checklist is used by health-care workers, and to closely monitor and record implementation barriers and successes.

Ongoing support and evaluation

Implementers and facility leaders are encouraged to provide a mechanism for ongoing programme support. Supervised use of the checklist, ongoing coaching in its correct use, and troubleshooting implementation barriers will help to achieve sustained adoption of the programme. If possible, objective assessment of the programme's impact on health-care workers' practices or health outcomes of mothers and babies should be made.

If areas of improvement are identified, these should be shared with health-care workers regularly in order to help to promote continued programme success.

Support to Checklist users

It is important that personnel using this Checklist understand that its purpose is to improve birth practices and not to find fault with or penalize individuals, and that they receive training on how to use it.

The responsible officers in health-care institutions using the Checklist need to clarify who in the team should implement it, and once identified, the Checklist user should be given sufficient power to alert other members of the team to comply with the various items on the Checklist.

Acknowledgements

WHO Safe Childbirth Checklist Development Group

The WHO Safe Childbirth Checklist Programme is the result of a collaboration between the WHO Patient Safety Programme, the WHO Department of Maternal, Newborn, Child and Adolescent Health, the WHO Department of Reproductive Health and Research, and the Harvard School of Public Health.

WHO wishes to thank the following individuals for their contribution in this work

Core WHO Checklist Development Group

Atul Gawande and Jonathan Spector, *Harvard School of Public Health, USA*

Angela Lashoher, Gerald Dziekan and Itziar Larizgoitia, *WHO Patient Safety Programme*

Matthews Mathai and Rajiv Bahl *WHO Department of Maternal, Newborn, Child, and Adolescent Health*

Mario Merialdi, *WHO Department of Reproductive Health and Research*

WHO Safe Childbirth Checklist and Manual Drafting Group

WHO wishes to express its gratitude to the following individuals who contributed as part of the larger collaboration network of international experts in maternal and newborn health towards the development of the Safe Childbirth Checklist programme, and who provided valuable input and collaborated in the testing of the checklist.

Priya Agrawal
Harvard School of Public Health, USA

Atte Aitkoski
Nkoaranga Village, Arusha, Tanzania

Sabaratnam Arulkumaran
St George's Hospital Medical School, UK

Mavis Asiedu-Asamoah
Ikraram, Nigeria

Angela Bader
Brigham and Women's Hospital, USA

Mohamed Bassiouny
Mansoura, Egypt

B Benachinamaradi
Medical Officer in Charge, Gokak, India

William Berry
Harvard School of Public Health, USA

Shereen Bhutta
Jinnah Postgraduate Medical Centre, Pakistan

Zulfiqar Bhutta
Aga Khan University Medical Centre, Pakistan

Ann Blanc
EngenderHealth, USA

Dan Boorman
Boeing, USA

Waldemar Carlo
University of Alabama at Birmingham, USA

Susan Crowther
New Zealand College of Midwives and Midwifery Council, New Zealand

Gary Darmstadt
Bill and Melinda Gates Foundation, USA

Louise Day
LAMB Integrated Rural Health and Development, Bangladesh

Mark Dybul
The Children's Investment Fund Foundation, UK

Lizzie Edmondson
Tennessee Coalition Against Domestic and Sexual Violence, USA

Barbara Farlow
Patients for Patient Safety, Canada

Zhao Gengli
Peking University First Hospital, China

Kathleen Hill
University Research Co., LLC, USA

THIS PILOT EDITION IS FOR RESEARCH PURPOSES

Elizabeth Hizza
Dar es Salaam, Tanzania

Justus Hofmeyr
University of the Witwatersrand, South Africa

Narayan Honnungar
Jawaharlal Nehru Medical College, India

Julia Hussein
Initiative for Maternal Mortality Programme Assessment (IMMPACT), UK

Atanu Kamar Jana
Christian Medical College, India

Cate Kamau
Aqua Essential Services, Kenya

Khalid Khan
University of Birmingham, UK

Grace Kodindo
Mailman School of Public Health, Columbia University, USA

Bhala Kodkany
KLE University Research Foundation, India

Barbara Kwast
Averting Maternal Death and Disability (AMDD), The Netherlands

Tina Lavender
University of Manchester, UK

Claire Lemer
North West London National Health Service, UK

Bridget Lynch
International Confederation of Midwives, The Netherlands

Rashad Massoud
University Research Co., LLC, USA

Jane Machira
Nyen Provincial General Hospital, Kenya

Claudia Morrissey
Save The Children/Saving Newborn Live, USA

Elizabeth Morse
Brigham and Women's Hospital, USA

Susan Niermeyer
American Academy of Pediatrics, USA

Leonard Okoko
Kitui District Hospital, Kenya

Vinod Paul
All India Institute of Medical Sciences, India

Parmanaban Packirisamy
National Health Systems Resource Centre for Training and Research in Newborn Care, India

Robert Pattinson
University of Pretoria, South Africa

Amit Revankar
Jawaharlal Nehru Medical College, India

Craig Rubens
Seattle Children's Hospital, USA

Zakari Saley
Mali

Harshad Sanghvi
Jhpiego, USA

Susan Sheridan
Consumers Advancing Patient Safety, USA

Youssef Tawfik
USAID, USA

Phommady Vesaphong
Silk Home Project, Laos

Jean-José Wolomby-Molondo
University of Kinshasa Democratic Republic of the Congo

Linda Wright
National Institute of Health (NIH) and National Institute of Child Health and Human Development (NICHD), USA

Blair Wylie
Massachusetts General Hospital, USA

Juliana Yartey
UNICEF, USA

Jing Yuan
Peking University First Hospital, China

WHO Safe Childbirth Checklist Concept Designer

Chris Barnes
Emphatic Communications, USA

WHO Contributors

WHO also wishes to acknowledge the contribution of the following WHO Staff who provided technical contribution:

Benedetta Allegranzi, Carmen Audera-Lopez, Gwendolen Eamer, William Perry, Kevin Wang and Sepideh Bagheri Nejad of the *WHO Patient Safety Programme; and* Nigel Rollins and Wilson Were of the *WHO Department of Newborn, Child and Adolescent Health*
Also, the secretarial and editorial support of Armorel Duncan and Laura Pearson.

WHO would like to thank the following individuals for their guidance and leadership in the development of the checklist and management of the programme:

Najeeb Al-Shorbaji, *ai Director WHO Patient Safety Programme;*
Marie-Paule Kieny, *Assistant Director-General for Innovation, Information, Evidence and Research;*
Sir Liam Donaldson, *WHO Envoy for Patient Safety.*

References

1. Haynes ABet al. A Surgical Safety Checklist to Reduce Morbidity and Mortality in a Global Population. *The New England Journal of Medicine*, 2009, 360(5):491-9.
2. Pronovost P et al. An Intervention to Decrease Catheter-Related Bloodstream Infections in the ICU. *The New England Journal of Medicine,* 2006,355(26):2725–32.
3. Spector JM et al. Improving Quality of Care for Maternal and Newborn Health: Prospective Pilot Study of the WHO Safe Childbirth Checklist Program. *PLoS ONE,* 2012, 7(4): e35151.
4. WHO. World Health Organization partograph in management of labour. *Lancet,* 1994, 343:1399-404.
5. *Pregnancy, Childbirth, Postpartum and Newborn Care: A guide for essential practice.* Geneva, World Health Organization, 2006.
6. *Managing prolonged and obstructed labour.* Geneva, World Health Organization, 2008.
7. WHO. *WHO Guidelines for Safe Surgery.* Geneva, World Health Organization, 2009.
8. *WHO recommendations for Prevention and treatment of pre-eclampsia and eclampsia.* Geneva, World Health Organization, 2011.
9. *Antiretroviral drugs for treating pregnant women and preventing HIV infection in infants: recommendations for a public health approach.* Geneva,World Health Organization, 2010.
10. Hodnett ED et al. Continuous support for women during childbirth. *The Cochrane Database of Systematic Reviews*, 2007, (3):CD003766.
11. *Managing Complications in Pregnancy and Childbirth: A guide for midwives and doctors.* Geneva, World Health Organization, 2007.
12. *WHO Pocket book of hospital care for children: guidelines for the management of common illnesses with limited resources.* Geneva, World Health Organization, 2005.
13. *WHO Guidelines on basic newborn resuscitation.* Geneva,World Health Organization, 2012.
14. Singhal N et al. Helping Babies Breathe: Global neonatal resuscitation program development and formative educational evaluation. *Resuscitation,* 2011.
15. *WHO guidelines for the management of postpartum haemorrhage and retained placenta*. Geneva, World Health Organization, 2009.
16. *Education material for teachers of midwifery. Midwifery education modules – second edition. Managing postpartum haemorrhage.* Geneva, World Health Organization, 2008.
17. *Managing Newborn Problems: A guide for doctors, nurses, and midwives.* Geneva, World Health Organization, 2003.
18. *Recommendations for management of common childhood conditions.* Geneva, World Health Organization, 2012.
19. WHO and UNICEF. *Integrated Management of Childhood Illness: Caring for Newborns and Children in the Community.* Geneva, World Health Organization, 2011.
20. *Thermal Protection of the Newborn: A practical guide.* Geneva, World Health Organization,1997.
21. WHO, UNAIDS, UNFPA and UNICEF. *Guidelines on HIV and infant feeding: principles and recommendations for infant feeding in the context of HIV and a summary of evidence.* Geneva, World Health Organization, 2010.
22. *Infant and young child feeding: Model Chapter for textbooks for medical students and allied health professionals.* Geneva, World Health Organization, 2009.
23. *The World Health Report 2005: Make every mother and child count.* Geneva, World Health Organization, 2005.
24. WHO and UNICEF. *Home visits for the newborn child: A strategy to improve survival.* Geneva, World Health Organization, 2009.

THIS PILOT EDITION IS FOR RESEARCH PURPOSES

25. *Medical eligibility criteria for contraceptive use, 4th ed: A WHO family planning cornerstone.* Geneva, World Health Organization, 2010.

26. UNDP/UNFPA/WHO/ World Bank Special Programme of Research, Development and Research Training in Human Reproduction (HRP). *Progestogen-only contraceptive use during lactation and its effects on the neonate.* Geneva, World Health Organization, 2008 (Statement).

27. *Combined hormonal contraceptive use during the postpartum period.* Geneva, World Health Organization, 2010 (Statement).

28. *Mother-Baby Package: Implementing safe motherhood in countries.* Geneva, World Health Organization,1996.

29. Pronovost PJ et al. Creating high reliability in health care organizations. *Health Services Research,* 2006,41(4 Pt 2):1599-617.

30. King HB et al. TeamSTEPPS™: Team Strategies and Tools to Enhance Performance and Patient Safety. In: Henriksen Ket al (editors). *Advances in Patient Safety: New Directions and Alternative Approaches (Vol 3: Performance and Tools).* Rockville, MD, Agency for Healthcare Research and Quality, 2008.

31. Pronovost PJ, Berenholtz SM, Needham DM. Translating evidence into practice: a model for large scale knowledge translation. *BMJ*, 2008,337:a1714.

32. Caldwell D et al. Implementing strategic change in a health care system: the importance of leadership and change readiness. *Health Care Management Review*, 2008, 33(2):124-33.

World Health Organization

World Health Organization
20 Avenue Appia
CH-1211 Geneva 27
Switzerland
Tel.: +41 22 791 5060

Email: patientsafety@who.int
Please visit us at:
www.who.int/patientsafety

9 789241 504140

Appendix C: HEALTH INSTITUTION CONSENT FORM

Appendix C: HEALTH INSTITUTION CONSENT FORM

Project Information	
Principal Investigator: Prof. Dr. Sebastian Vollmer	Organization: Georg-August University of Goettingen
Location:<br>Georg-August Universität Goettingen<br>Platz der Goettinger Sieben 3<br>37073 Goettingen<br>Germany	Lennart Kaplan<br>Phone: +49 (0) 6221 - 543739<br>Email: lennart.kaplan@wiwi.uni-goettingen.de<br><br>Katharina Richert<br>Phone: +49 (0) - 6221 - 543739<br>Email: Katharina.Richert@awi.uni-heidelberg.de
Other Investigators: Lennart Kaplan<br><br>Katharina Richert	Organization: Georg-August University of Goettingen<br><br>Organization: Heidelberg University

My name is ___________________ and I am from a social research organization [NAME]. Currently I am working with a team from the Universities in Goettingen, Germany and Syiah Kuala University, Indonesia. We are conducting a survey about safe childbirth practices in public health facilities in Aceh. Although the research we conduct is independent from the government, the district health offices of Aceh support its introduction and the Ethical Clearance Committee at the Medical Faculty of Syiah Kuala University has given approval to conduct this study [show support letters].

The World Health Organization has developed the Safe Childbirth Checklist. This is a tool that integrates the international best practices during childbirth into checklist items that shall support the health personnel concerned with delivery to remember all important steps during childbirth and hence, ensure a standardized quality standard during all births in your health facility. The Safe Childbirth Checklist will be introduced in the participating health facilities through workshops to explain and motivate its use.

The aim of the study is that in the end, the checklist will be introduced in all participating public health facilities. But due to budget constraints we cannot introduce the checklist together with a complementary training in all facilities at the same time. Hence, we will start to implement it in 50% of all health facilities and the other half of the facilities will get it within the first half of 2017 in the case that the instrument proves effective. It will be determined by a lottery which institution will get the intervention first. Right now we are visiting all health facilities to ask their consent to take part in the research study. By the mid of 2016, a person will come back to your health institution and tell you if your facility got selected for the implementation at the mid of 2016 or in the first half of 2017.

Before we start to introduce the Safe Childbirth Checklist in the health facilities, we would like to ask you some general questions about your health facility, including number of staff and patients, and observe the birth process. Furthermore, we would like to ask your health personnel that is concerned with delivery services some questions on their work environment, including some information about teamwork and communication structures.

We are planning to come back to your health facility after six months to ask you similar questions again. Any new information developed during the study that may affect your willingness to continue participation will be communicated to you. The interview with you will last for approximately 15 minutes, with your health personnel about 20 minutes. Whatever information you provide will be kept strictly confidential and anonymous. Bits of what you say will be stored on a computer and used to prepare a report that we write after we have conducted our research study. We are sharing the information that we collect with other trusted researchers from Indonesia and other countries. We hope this report will be helpful to local and national governments when planning childbirth services in the future.

Our research may not change things in the short term, because that depends on local and national governments. We are here to learn from you and your health personnel, but we cannot promise to improve things. Participation in this research study is voluntary. However, we hope that you will participate in this survey since your participation is important to help us learn about childbirth practices in your province and throughout Indonesia.

In case you have any further questions, when I have already left, you can contact:

German research team

Lennart Kaplan
Email: lennart.kaplan@wiwi.uni-goettingen.de
Phone: +49 (0) 6221 – 543739
Mobile: +49 (0) 160 – 99 65 56 83

Katharina Richert
Email: Katharina.Richert@awi.uni-heidelberg.de
Phone: +49 (0) - 6221 - 543739

Do you have any question at this point? You can ask me anything that you did not understand or anything you want to know.

Would you like to participate?

CONSENT FORM

IDENTIFICATION INFORMATION		
II 1	Health Facility Name and ID	Name:<br>ID_HF: |___|___|___|___|

I, _________________________________, after being informed about all aspects of this project described in this format, and having all my questions and concerns about this project answered, I voluntarily accept to participate this project in my health facility. I commit myself and my health facility to support the procedures described above. I have had the opportunity to ask any questions related to the project. I understand the procedures of the project and how the information will be treated in a confidential manner, without revealing the identity of any person participating in the project in any result reported or published. I give my authorization to give access to this information to all members of the research team, knowing that this information will be used confidentially. I understand that my consent does not take away any legal rights in the case of negligence or other legal fault of anyone who is involved in this study. I further understand that nothing in this consent form is intended to replace any applicable federal, state, or local laws.

Principal Name (Printed or Typed):

Principal Signature:

Date:

Field Worker Name (Printed or Typed):

Signature of Field Worker:

Date:

Appendix D: Health Personnel Consent Form

Appendix D: HEALTH PERSONNEL CONSENT FORM

**Project Information**	
Principal Investigator: Prof. Dr. Sebastian Vollmer	Organization: Georg-August University of Goettingen
Location:<br>Georg-August Universität<br>Goettingen<br>Platz der Goettinger Sieben 3<br>37073 Goettingen<br>Germany	Lennart Kaplan<br>Phone: +49 (0) 6221 - 543739<br>Email: lennart.kaplan@wiwi.uni-goettingen.de<br><br>Katharina Richert<br>Phone: +49 (0) - 6221 - 543739<br>Email: Katharina.Richert@awi.uni-heidelberg.de
Other Investigators: Lennart Kaplan<br><br>Katharina Richert	Organization: Georg-August University of Goettingen<br><br>Organization: Heidelberg University

General Fieldworker note: No project staff should pressurize, coerce or deceive respondents in an effort to ensure their participation. Staff should also try to ensure that respondents are not pressurized by other health personnel or by principal of health facility. Staff should not make any promises they cannot or are unlikely to keep. The respondents will be free to withdraw from the study at any time. Whilst the study procedures are designed to ensure that consent is informed and voluntary, the only person who can really ensure that is you, the fieldworker. You must make every effort to make sure the participants understand the study and feel free not to take part or to withdraw if they wish to.

Fieldworker note: Introduce yourself.

My name is ___________________ and I am from a social research organization [NAME].
Currently I am working with a team of researchers from Germany and Indonesia.

Fieldworker note: Explain the purpose of the study and what the study is about.

We are conducting a research study about safe childbirth practices in public health facilities in Aceh province. Your health facility has been chosen to participate in the study. The principal of your health facility [NAME] has agreed to take part in the study and has allowed us to select you for a short questionnaire. [The study has been approved by the Government of Indonesia and is supported by the district health office. The Medical Ethics Committee from the University of Goettingen in Germany and the Ethical Clearance Committee of Syiah Kuala University approved the study.]

Fieldworker note: Explain what the participant is supposed to do if she/he decides to participate.

We would like to ask you some questions about your work here at the health facility, including some information about teamwork and communication structures, and your general satisfaction within your job

and everyday work processes. There are no right or wrong answers; we just want to learn more about your work as [gynecologist/obstetrician/midwife/lady health worker/nurse] in this facility as you play an important role in the childbirth practices in this facility. We are planning to come back to your health facility after five to six months to ask you similar questions concerning your work. Any new information developed during the study that may affect your willingness to continue participation will be communicated to you.
Apart from you and some of your colleagues, we are also going to ask the same questions to other health personnel working in different health facilities in the province of Aceh.

Fieldworker note: Explain how long the interview and investigations take.

The interview will last for approximately 20 minutes.

Fieldworker note: Ensure that all information is confidential and anonymous.

Whatever information you provide will be kept strictly confidential. This means what you will say will be shared with other members of the research team, but I am not going to tell your colleagues, your principal, or anybody in the community what you tell me. Your name will not be used so we can describe what you think without anyone knowing that it is you. We will also disguise the name of the health facility you are working in.

Fieldworker note: Explain about archiving procedure.

Bits of what you say will be stored on a computer and used to prepare a report that we write after we have talked to all the health personnel. We are sharing the information that we collect with other trusted researchers from Indonesia and other countries. We hope this report will be helpful to local and national governments when trying to improve childbirth practices in health facilities in the future.

Fieldworker note: Ensure that you do not raise expectations.

Our research may not change things in the short term, because that depends on local and national governments. We are here to learn from you, but we cannot promise to improve your working environment.

Fieldworker note: Ensure that participant understands that she/he can drop out or not answer any of the questions at any point.

Participation in this survey is voluntary and you can choose not to answer any question or all of the questions. You have the right to reject your participation or to stop participating in this study at any time that you want. You are also free to answer or not to any questions that you want. You are free to change your mind at any time during this project, without affecting your job. However, we hope that you will participate in this survey since your participation is important to help us learn about childbirth practices in health facilities in Aceh and Indonesia.

Fieldworker note: Give the participant contact details of a person in charge of the project who can be consulted in case questions arise after the interview is finished and hand out a sheet of paper which gives all the information you just have written out.

In case you have any further questions, when I have already left, you can contact:

German research team

Lennart Kaplan
Email: lennart.kaplan@wiwi.uni-goettingen.de
Phone: +49 (0) 6221 – 543739
Mobile: +49 (0) 160 – 99 65 56 83

Katharina Richert
Email: Katharina.Richert@awi.uni-heidelberg.de
Phone: +49 (0) - 6221 - 543739

Fieldworker note: Give the participant the chance and sufficient time to formulate a question.

Do you have any question at this point? You can ask me anything that you did not understand or anything you want to know.

Would you like to participate?

Fieldworker note: If participant responds "no" ask again if she/he has some questions, which prevent her/him from participating. If she/he still does not want to participate stop the interview. If she/he responds "yes" ask her/him to sign the consent form.

May I ask you to sign this consent form?

CONSENT FORM

IDENTIFICATION INFORMATION		
II 1	Health Facility Name and ID	ID_HF: └───┴───┴───┴───┘
II2	Health Personnel Name and ID	ID_HP: └───┴───┴───┴───┘

QUESTIONNAIRE NUMBER: ______________________________

I, __________________________________, have read and understood the consent form, and I volunteer to participate in this research study. I understand that I will receive a copy of this form. I voluntarily choose to participate, but I understand that my consent does not take away any legal rights in the case of negligence or other legal fault of anyone who is involved in this study. I further understand that nothing in this consent form is intended to replace any applicable Federal, state, or local laws.

Participant Name (Printed or Typed):

Participant Signature:

Date:

Field Worker Name (Printed or Typed):

Signature of Field Worker:

Date:

Appendix E: PATIENT CONSENT FORM FOR FOLLOW-UP CONTACT

Appendix E: PATIENT CONSENT FORM FOR FOLLOW-UP CONTACT

**Project Information**	
Principal Investigator: Prof. Dr. Sebastian Vollmer	Organization: Georg-August University of Goettingen
Location:<br>Georg-August Universität<br>Goettingen<br>Platz der Goettinger Sieben 3<br>37073 Goettingen<br>Germany	Lennart Kaplan<br>Phone: +49 (0) 6221 - 543739<br>Email: lennart.kaplan@wiwi.uni-goettingen.de<br><br>Katharina Richert<br>Phone: +49 (0) - 6221 - 543739<br>Email: Katharina.Richert@awi.uni-heidelberg.de
Other Investigators: Lennart Kaplan<br><br>Katharina Richert	Organization: Georg-August University of Goettingen<br><br>Organization: Heidelberg University

General note: No facility staff should pressurize, coerce or deceive respondents in an effort to ensure their participation. Staff should not make any promises the research team cannot or are unlikely to keep. The respondents will be free to withdraw from the study at any time. Whilst the study procedures are designed to ensure that consent is informed and voluntary, the only person who can really ensure that is you. You must make every effort to make sure the participants understand the study and feel free not to take part or to withdraw if they wish to.

Health worker note: Introduce yourself.

My name is ___________________, employed at the health facility [NAME].
Currently our facility is participating in a research study by a team of researchers from Germany and Indonesia in order to improve the quality of maternal and neonatal care. As you have been a patient in this health facility, we would like to ask for your consent to share your contact details with the international research team in order to allow for follow-up contact.

Health worker note: Explain what the participant is supposed to do if she/he decides to participate.

The research team would like to ask you some questions about your personal experience within this health facility during your latest childbirth, including some information about your satisfaction with their service quality and also general information about you, including your age, education, and health during pregnancy. Apart from you, the same questions will also be asked to other patients from this and other health facilities that are part of the study. Above that, you might be invited for a group discussion led by the international research team.

Health worker note: Ensure that all information is confidential and anonymous.

Your contact details will be treated with highest confidentiality and would be only shared with the researchers involved in this project. Whatever further information you provide will be kept strictly confidential.

Health worker note: Ensure that you do not raise expectations.

This research project was set up to learn from you and your experience during childbirth and it may not change things in the short term, because that depends on local and national governments.

Health worker note: Ensure that participant understands that she/he can drop out or not answer any of the questions at any point.

Participation in this study is voluntary and you can choose to reject your participation at any later point in time. You would also be free to answer or not to any questions that you want. You are free to change your mind at any time during this project. However, we hope that you will provide your contact details since your participation is important to learn about childbirth in this regency and throughout Indonesia.

Health worker note: Give the participant contact details of a person in charge of the project who can be consulted in case questions arise after the interview is finished and hand out a sheet of paper which gives all the information you just have written out.

In case you have any further questions when I have already left you can contact:
German research team

Lennart Kaplan
Email: lennart.kaplan@wiwi.uni-goettingen.de
Phone: +49 (0) 6221 – 543739
Mobile: +49 (0) 160 – 99 65 56 83

Katharina Richert
Email: Katharina.Richert@awi.uni-heidelberg.de
Phone: +49 (0) - 6221 - 543739

Health worker note: If participant responds “no” ask again if she/he has some questions, which prevent her/him from participating. If she/he still does not want to participate do not collect consent. If she/he responds “yes” ask her/him to sign the consent form.

May I ask you to sign this consent form?

CONSENT FORM

IDENTIFICATION INFORMATION		
**II1**	Health Facility Name and ID	ID_HF: |___|___|___|___|
**II2**	Patient Name and ID	ID_P: |___|___|___|___|

I, __________________________________, have read and understood the consent form, and I agree to share my contact details in the framework of this research study. I understand that I will receive a copy of this form. I voluntarily choose to share my contact details, but I understand that my consent does not take away any legal rights in the case of negligence or other legal fault of anyone who is involved in this study. I further understand that nothing in this consent form is intended to replace any applicable Federal, state, or local laws.

Patient Name (Printed or Typed):

Patient Address:

Patient Signature:

Date:

Health Worker Name (Printed or Typed):

Signature of Health Worker:

Date:

Appendix F: Patient Consent Form

Appendix F: PATIENT CONSENT FORM

**Project Information**	
Principal Investigator: Prof. Dr. Sebastian Vollmer	Organization: Georg-August University of Goettingen
Location:<br>Georg-August Universität<br>Goettingen<br>Platz der Goettinger Sieben 3<br>37073 Goettingen<br>Germany	Lennart Kaplan<br>Phone: +49 (0) 6221 - 543739<br>Email: lennart.kaplan@wiwi.uni-goettingen.de<br><br>Katharina Richert<br>Phone: +49 (0) - 6221 - 543739<br>Email: Katharina.Richert@awi.uni-heidelberg.de
Other Investigators: Lennart Kaplan<br><br>Katharina Richert	Organization: Georg-August University of Goettingen<br><br>Organization: Heidelberg University

General Fieldworker note: No project staff should pressurize, coerce or deceive respondents in an effort to ensure their participation. Staff should also try to ensure that respondents are not pressurized by other household or community members, nor by health staff at the facility. Staff should not make any promises they cannot or are unlikely to keep. The respondents will be free to withdraw from the study at any time. Whilst the study procedures are designed to ensure that consent is informed and voluntary, the only person who can really ensure that is you, the fieldworker. You must make every effort to make sure the participants understand the study and feel free not to take part or to withdraw if they wish to.

Fieldworker note: Introduce yourself.

My name is ____________________ and I am from a social research organization [NAME].
Currently I am working with a team of researchers from Germany and Indonesia.

Fieldworker note: Explain the purpose of the study and what the study is about.

We are conducting a research study about safe childbirth practices in public health facilities in Aceh province. As you have been a patient in one of our studied health facilities, you have been chosen to participate in this survey. You have given your consent to the health facility to share your contact details with us. That is how we were able to choose you for our short questionnaire. [The study has been approved and is supported by the Government of Indonesia and by the Medical Ethics Committee from the University of Goettingen in Germany and the Ethical Clearance Committee at the Medical Faculty of Syiah Kuala University.]

Fieldworker note: Explain what the participant is supposed to do if she/he decides to participate.

We would like to ask you some questions about your personal experience within this health facility during your latest childbirth, including some information about your satisfaction with their service quality and also general information about you, including your age, education, and health during pregnancy.
There are no right or wrong answers; we just want to learn about childbirth services in your province.

Apart from you, we are also going to ask the same questions to other patients from this and other health facilities that are part of our study.

Fieldworker note: Explain how long the interview and investigations take.

The interview will last for approximately 15 to 20 minutes.

Fieldworker note: Ensure that all information is confidential and anonymous.

Whatever information you provide will be kept strictly confidential. This means what you will say will be shared with other members of the research team, but I am not going to tell your doctor, family or anyone in your community what you tell me. Your name will not be used so we can describe what you think without anyone knowing that it is you.

Fieldworker note: Explain about archiving procedure.

Bits of what you say will be stored on a computer and used to prepare a report that we write after we have talked to the patients. We are sharing the information that we collect with other trusted researchers from Indonesia and other countries. We hope this report will be helpful to local and national governments, when planning childbirth services in the future.

Fieldworker note: Ensure that you do not raise expectations.

Our research may not change things in the short term, because that depends on local and national governments. We are here to learn from you and your experience during childbirth, but we cannot promise to improve your or your family's life.

Fieldworker note: Ensure that participant understands that she/he can drop out or not answer any of the questions at any point.

Participation in this survey is voluntary and you can choose not to answer any question or all of the questions. You have the right to reject your participation or to stop participating in this study at any time that you want. You are also free to answer or not to any questions that you want. You are free to change your mind at any time during this project. However, we hope that you will participate in this survey since your participation is important to help us learn about childbirth in your regency and throughout Indonesia.

Fieldworker note: Give the participant contact details of a person in charge of the project who can be consulted in case questions arise after the interview is finished and hand out a sheet of paper which gives all the information you just have written out.

In case you have any further questions when I have already left you can contact:
German research team

Lennart Kaplan
Email: lennart.kaplan@wiwi.uni-goettingen.de
Phone: +49 (0) 6221 – 543739
Mobile: +49 (0) 160 – 99 65 56 83

Katharina Richert
Email: Katharina.Richert@awi.uni-heidelberg.de
Phone: +49 (0) - 6221 - 543739

Fieldworker note: Give the participant the chance and sufficient time to formulate a question.

Do you have any question at this point? You can ask me anything that you did not understand or anything you want to know.
Would you like to participate?

Fieldworker note: If participant responds “no” ask again if she/he has some questions, which prevent her/him from participating. If she/he still does not want to participate stop the interview. If she/he responds “yes” ask her/him to sign the consent form.

May I ask you to sign this consent form?

CONSENT FORM

IDENTIFICATION INFORMATION		
**II1**	Health Facility Name and ID	ID_HF: |___|___|___|___|
**II2**	Patient Name and ID	ID_P: |___|___|___|___|

QUESTIONNAIRE NUMBER: ____________________________

I, ________________________________, have read and understood the consent form, and I volunteer to participate in this research study. I understand that I will receive a copy of this form. I voluntarily choose to participate, but I understand that my consent does not take away any legal rights in the case of negligence or other legal fault of anyone who is involved in this study. I further understand that nothing in this consent form is intended to replace any applicable Federal, state, or local laws.

Participant Name (Printed or Typed):

Participant Signature:

Date:

Field Worker Name (Printed or Typed):

Signature of Field Worker:

Date:

Appendix G: Delivery Skills Assessment Consent Form

Appendix G: DELIVERY SKILLS ASSESSMENT CONSENT FORM

Project Information	
Principal Investigator: Prof. Dr. Sebastian Vollmer	Organization: Georg-August University of Goettingen
Location:<br>Georg-August Universität<br>Goettingen<br>Platz der Goettinger Sieben 3<br>37073 Goettingen<br>Germany	Lennart Kaplan<br>Phone: +49 (0) 6221 - 543739<br>Email: lennart.kaplan@wiwi.uni-goettingen.de<br><br>Katharina Richert<br>Phone: +49 (0) - 6221 - 543739<br>Email: Katharina.Richert@awi.uni-heidelberg.de
Other Investigators: Lennart Kaplan<br><br>Katharina Richert	Organization: Georg-August University of Goettingen<br><br>Organization: Heidelberg University

General Fieldworker note: No project staff should pressurize, coerce or deceive respondents in an effort to ensure their participation. Staff should also try to ensure that respondents are not pressurized by other health personnel or by principal of health facility. Staff should not make any promises they cannot or are unlikely to keep. The respondents will be free to withdraw from the study at any time. Whilst the study procedures are designed to ensure that consent is informed and voluntary, the only person who can really ensure that is you, the fieldworker. You must make every effort to make sure the participants understand the study and feel free not to take part or to withdraw if they wish to.

Fieldworker note: Introduce yourself.

My name is ___________________ and I am from a social research organization [NAME].
Currently I am working with a team of researchers from Germany and Indonesia.

Fieldworker note: Explain the purpose of the study and what the study is about.
We are conducting a research study about safe childbirth practices in public health facilities in the province of Aceh. You have been chosen to participate in the study. The principal of your health facility [NAME] has agreed to take part in the study and has allowed us to select you for this study. [The study has been approved by the Government of Indonesia and is supported by the district health office. The medical ethics committee from the University of Goettingen in Germany and the Ethical Clearance Committee at the Medical Faculty of Syiah Kuala University approved the study.]

Fieldworker note: Explain what the participant is supposed to do if she/he decides to participate.

We would like to ask you some questions about your work here at the health facility. The questions will be part of a "Delivery Skills Assessment" in order to assess your competence in skills such as communication, clinical examination, medical procedures and interpretation of diagnostic results. This might also include a practical evaluation of your every-day clinical work using an OSCE format (Objective Structured Clinical Examination). The OSCE is a modern type of examination often used in health sciences. It is designed to test clinical skill performance and various competences. The "Delivery Skills Assessment OSCE" will comprise a circuit of short (5-15min) stations, in which each candidate is examined on a one-to-one basis

with one or two impartial examiners and simulated patients (actors or simulators). Each station has a different examiner. Candidates rotate through the stations and complete all the stations on their circuit. In this way, all the candidates take the same standardized stations, enabling a fair peer comparison.
”The Delivery Skills Assessment OSCE” will inform us on how to best design a training package that matches your current set of skills as well as that of your peers. This tailored training will be made available to ***all*** participants of this study throughout the course of this year. We hope that this will further improve your clinical expertise, given the vital role you play in the childbirth practices in this facility.
We are planning to come back to you and repeat the “Delivery Skills Assessment OSCE” after around 4 to 6 months after the training to see how the training was able to improve your skills in the long term. Any new information developed during the study that may affect your willingness to continue participation will be communicated to you. Apart from you, we are also going to ask the same questions and perform the same “Delivery Skills Assessment OSCE” with other health personnel working in the province of Aceh.

Fieldworker note: Explain how long the interview and investigations take.

The interview and “Delivery Skills Assessment OSCE” will last approximately 1h.

Fieldworker note: Ensure that all information is confidential and anonymous.

Whatever information you provide as well as the results of your “Delivery Skills Assessment OSCE” will be kept strictly confidential. This means what you will say and how you perform will be shared with other members of the research team, but I am not going to tell your colleagues, your principal/supervisor, or anybody in the community what you tell me or how you scored. Your name will not be used so we can describe what you think without anyone knowing that it is you. We will also disguise the name of the health facility you are working in.

Fieldworker note: Explain about archiving procedure.

Bits of what you say will be stored on a computer and used to prepare a report that we write after we have assessed all the health personnel participating in our study. We are sharing the information that we collect with other trusted researchers from Indonesia and other countries. We hope this report will be helpful to local and national governments when trying to improve childbirth practices in the future.

Fieldworker note: Ensure that you do not raise expectations.

Our research may not change things in the short term, because that depends on local and national governments. We are here to learn from you, but we cannot promise to improve your working environment.

Fieldworker note: Ensure that participant understands that she/he can drop out or not answer any of the questions at any point.

Participation in this study is voluntary and you can choose not to answer any question or all of the questions as well as not to perform the “Delivery Skills Assessment OSCE”. You have the right to reject your participation or to stop participating in this study at any time that you want. You are also free to answer or not to any questions that you want or to drop out of the “Delivery Skills Assessment OSCE”. You are free to change your mind at any time during this project, without affecting your job. However, we hope that you will participate in this study since your participation is important to help us learn about childbirth practices in your regency and throughout Indonesia.

Fieldworker note: Give the participant contact details of a person in charge of the project who can be consulted in case questions arise after the interview is finished and hand out a sheet of paper which gives all the information you just have written out.

In case you have any further questions when I have already left you can contact:
German research team

Lennart Kaplan
Email: lennart.kaplan@wiwi.uni-goettingen.de
Phone: +49 (0) 6221 – 543739
Mobile: +49 (0) 160 – 99 65 56 83

Katharina Richert
Email: Katharina.Richert@awi.uni-heidelberg.de
Phone: +49 (0) - 6221 - 543739

Fieldworker note: Give the participant the chance and sufficient time to formulate a question.

Do you have any question at this point? You can ask me anything that you did not understand or anything you want to know.
Would you like to participate?

Fieldworker note: If participant responds “no” ask again if she/he has some questions, which prevent her/him from participating. If she/he still does not want to participate stop the interview. If she/he responds “yes” ask her/him to sign the consent form.

May I ask you to sign this consent form?

CONSENT FORM

IDENTIFICATION INFORMATION		
II1	Health Facility Name and ID	ID_HF: |___|___|___|___|
II2	Health Personnel Name and ID	ID_HP: |___|___|___|___|
II3	Health Personnel Designation	□ General Practitioner<br>□ Gynecologist<br>□ Obstetrician<br>□ FMO<br>□ LHV<br>□ Nurse<br>□ Midwife<br>□ Other<br>Specify:

QUESTIONNAIRE NUMBER: ______________________________

I, __________________________________, have read and understood the consent form, and I volunteer to participate in this research study. I understand that I will receive a copy of this form. I voluntarily choose to participate, but I understand that my consent does not take away any legal rights in the case of negligence or other legal fault of anyone who is involved in this study. I further understand that nothing in this consent form is intended to replace any applicable Federal, state, or local laws.

Participant Name (Printed or Typed):

Participant Signature:

Date:

Field Worker Name (Printed or Typed):

Signature of Field Worker:

Date:
