# Supplementary material for: Effects of the World Health Organization Safe Childbirth Checklist on Quality of Care and Birth Outcomes in Aceh, Indonesia: A Cluster-Randomized Clinical Trial
Source: JAMA Netw Open. 2021 Dec 3;4(12):e2137168. doi: 10.1001/jamanetworkopen.2021.37168 (PMC8642783; doi:10.1001/jamanetworkopen.2021.37168)
Supplement: Supplement 2. — eAppendix. Supplementary Materials eFigure 1. Mortality and Morbidity (Subsample of Facilities Where Observations Were Conducted) eFigure 2. Adapted SCC eTable 1. Minimal Detectable Effect Sizes for Applied Essential Practices (Different % of Take-up) eTable 2. Minimal Detectable Effect Sizes for Health Outcomes eTable 3. Mortality (Individual Births) eTable 4. Mortality and Morbidity (Facility Level Rates) eTable 5. Mortality and Morbidity (Subsample of Facilities Where Observations Were Conducted) eTable 6. Point Estimates for Covariates eTable 7. Case Numbers per Facility eTable 8. Depiction of Safe Childbirth Checklist and Research Team Exposure Among Treatment and Control Group eTable 9. Background Information—Complications [file jamanetwopen-e2137168-s002.pdf]

Supplementary Online Content

Kaplan LC, Ichsan I, Diba F, et al. Effects of the World Health Organization Safe Childbirth Checklist on quality of care and birth outcomes in Aceh, Indonesia: a cluster-randomized clinical trial. *JAMA Netw Open*. 2021;4(12):e2137168. doi:10.1001/jamanetworkopen.2021.37168

eAppendix. Supplementary Materials

eFigure 1. Mortality and Morbidity (Subsample of Facilities Where Observations Were Conducted)

eFigure 2. Adapted SCC

eTable 1. Minimal Detectable Effect Sizes for Applied Essential Practices (Different % of Take-up)

eTable 2. Minimal Detectable Effect Sizes for Health Outcomes

eTable 3. Mortality (Individual Births)

eTable 4. Mortality and Morbidity (Facility Level Rates)

eTable 5. Mortality and Morbidity (Subsample of Facilities Where Observations Were Conducted)

eTable 6. Point Estimates for Covariates

eTable 7. Case Numbers per Facility

eTable 8. Depiction of Safe Childbirth Checklist and Research Team Exposure Among Treatment and Control Group

eTable 9. Background Information—Complications

This supplementary material has been provided by the authors to give readers additional information about their work.

© 2021 Kaplan LC et al. *JAMA Network Open*.

eAppendix: Supplementary Materials

Statistical analysis

For treatment assignment, we used the publicly available minMSE code in Stata.[26] We estimated Intention To Treat (ITT) effects and Complier Average Causal Effects (CACE) using Stata, whereas supplementary Generalized Linear Models (GLM) estimates were computed via the statistical computing package R. Finally, we used a publicly available program in Stata to adjust p-values for false discovery rates.[33,34]

Intention To Treat (ITT) Effect Analysis

The basic estimation equation for the Intention to Treat (ITT) effect reads as follows:

$$Y_{ij} = \alpha + \beta_1 T_i + \beta_2 X_i + \varepsilon_I \qquad (1)$$

Outcome Y_{ij} is a binary indicator with the value 1, if the respective practice was applied at facility i for birth j. T_i indicates if the facility was in the treatment group, X_i is a vector of covariates at the facility-level (facility type, urban-rural, CEmONC-status, district dummy), and ε_i is the error term.

Complier Average Causal Effect (CACE) Analysis

Our CACE approach builds on a two-step analysis, where the treatment allocation serves as an instrument to predict compliance in the first stage:

$$C_{ij} = \alpha + \beta_1 T_i + \beta_2 X_i + \varepsilon, \qquad (2)$$

Where C_{ij} indicates compliance (checklist use) at facility i for birth j. Predicted compliance $\hat{C}_{ij}$ is inserted into the

2nd stage to estimate Equation (3) analogous to Equation (1):

$$Y_{ij} = \alpha + \beta_1 \hat{C}_{ij} + \beta_2 X_i + \varepsilon_I, \qquad (3)$$

where Y_{ij} refers to outcomes at facility i for birth j, which are regressed on predicted compliance. Compliance is measured at the birth level by midwives actively using or looking at the SCC during clinical observations. When analyzing facility-level outcomes with a CACE, we measured compliance by calculating the number of completed SCCs over the total numbers of births. While individuals from the control group may also theoretically qualify as compliers if they would use the SCC (also labeled "always-takers" in the literature), this case did not materialize during observed births.[31] Our clustered trial design (provision of SCC at facility-level) made spill-overs very unlikely to happen. Cragg-Donald Wald F statistics were on both levels larger than 10, which suggests that the treatment is a sufficiently strong predictor of compliance to warrant reliable inference (e.g., we do not face weak instrumental variable issues which may inflate our estimates).

Penalized maximum likelihood logistic regression

Although neonatal and maternal mortality, as well as stillbirths in particular, impose a burden for the considered sample, their frequencies still qualify them as rare events in a statistical sense.. To account for this, we apply a penalized maximum likelihood logistic regression estimator, which applies a penalization to generalized linear models to correct for potential bias.[32] This approach is implemented via the firthlogit package in Stata.

Minimal detectable effects

eTables 1 and 2 describe minimal detectable effect sizes for the essential practices covered under the SCC as well as for health outcomes. We estimate minimal detectable effects based on the following formula:

$$MDE = \left(t_{1-\frac{\alpha}{2}} + \rho\right) \sqrt{\frac{\sigma^2}{Nf(1-f)}}$$

© 2021 Kaplan LC et al. *JAMA Network Open*.

where MDE refers to the minimal detectable effect, $t_{1-\frac{\alpha}{2}}$ to the level of intended statistical significance and ρ to the intended power (in our case 0.8). σ^2 is the variance, N indicates the number of observations per cluster and f the fraction of observations in treatment and control group. Effect sizes suggest that the study is sufficiently powered to analyze effects for essential practices, but that the sample was not sufficiently large to draw broader conclusions with regard to mortality and morbidity outcomes.

Mortality and morbidity (Facility-level estimates)

eTable 4 presents the outcomes for maternal and neonatal mortality and stillbirths (Rows 1, 2 and 3) as well as complications (Rows 4 and 5). Columns 2 and 4 in eTable 4 display the mean values in the treatment and control groups, whereas columns 5 and 7 depict our regression estimates from the ITT analysis and from the CACE estimation in columns 9 and 11. In the CACE estimations, the coefficients' confidence interval [-68.0 | 4.0] includes a larger range of negative values and turns out to be significant at the 10%-level when adding covariates. As only one maternal death was recorded in the 32 facilities over the six months intervention period results for maternal mortality are insignificant. With regard to stillbirths and neonatal mortality, eTable 3 indicates consistently negative coefficients for the ITT, which are in line with the expectations, but are generally insignificant. In the CACE estimations, the coefficient for neonatal mortality turns significant when adding covariates. However, those results should be treated cautiously as a reduction in the mean neonatal mortality rate in the treatment facilities from 2/1000 at baseline to 1.1/1000 at endline is contrasted by an increase in the mean of the control facilities from 2.7/1000 at baseline to 15/1000 at endline. Similarly, the stillbirth rate both increased in treatment and control group between base- and endline from 11/1000 to 17/1000 and from 13/1000 to 21/1000 respectively. Nonetheless, in line with the consistently negative signs across specifications, this provides some weak evidence that the SCC could contribute to a reduction in neonatal mortality. Effects for complication rates (Rows 4 and 5) are statistically insignificant, for a list of complications see eTable 9.

Background information – coaching approach:

The coaching comprised of three visits in the first month, two visits in the second, third and fourth month and finally one visit in the fifth and sixth month. The coaching consisted of a two-hour visit of the coach at the facility and (i) a meeting with the checklist quality coordinator (CQC), (ii) filling out a short survey on usage and barriers and (iii) an opportunity for consultation on correct SCC use. Additionally, when possible, the coaches (iv) provided feedback on observed births and (v) gave input regarding the previously collected checklists. CQCs were selected among the midwives to ensure regular use of the SCC and support other midwives with the application. The CQCs did not receive any remuneration.

Checklist adjustment

Together with local health staff, our team adjusted the checklist to the local context and needs as listed below:

- Change item
- Pause Point 2:
    - Confirm essential supplies are at bedside and prepare for delivery: Adapted item "Sterile blade to cut cord" to "Sterile scissors/knife to cut cord" to account for local practice
- Pause Point 3:
    - Is mother bleeding abnormally?: Added items "Yes, treat, but if cause cannot be treated, refer," and "If cause cannot be treated, refer based on your criteria" to accommodate the fact that community health centers (puskesmas) may not be able to address abnormal bleeding
    - Start breastfeeding and skin-to-skin contact (IMD) (if mother and baby are well): Added item "No, start later because mother or baby are not well" to provide midwives with an option to check if patient's status does not allow the starting of breastfeeding and skin to skin contact
- Pause Point 4:
    - Discuss and offer family planning options to mother: Add item "No, already done in antenatal care" and "No, will be done later" to allow midwives to check item if the point cannot be addressed immediately

Besides contextual adjustments, we added two major modifications according to practitioners' feedback. First, we included a slot for SCC users to note the time and date of given medication. This documentation should make it easier for the next user to assess which medication was needed and at what time. Second, we included a field to add information on the mother to attach it to the patient file (e.g., mother's name, age, weight, height). Third, we prepared a separate sheet for the danger signs to hand over to mothers and relatives before discharge, as a means for families to be better able to remember and assess situations in which they should return to the health facilities. An English version of the adapted SCC can be found in eFigure 2.

© 2021 Kaplan LC et al. *JAMA Network Open*.

eFigure 1: Mortality and Morbidity (Subsample of facilities where observations were conducted)

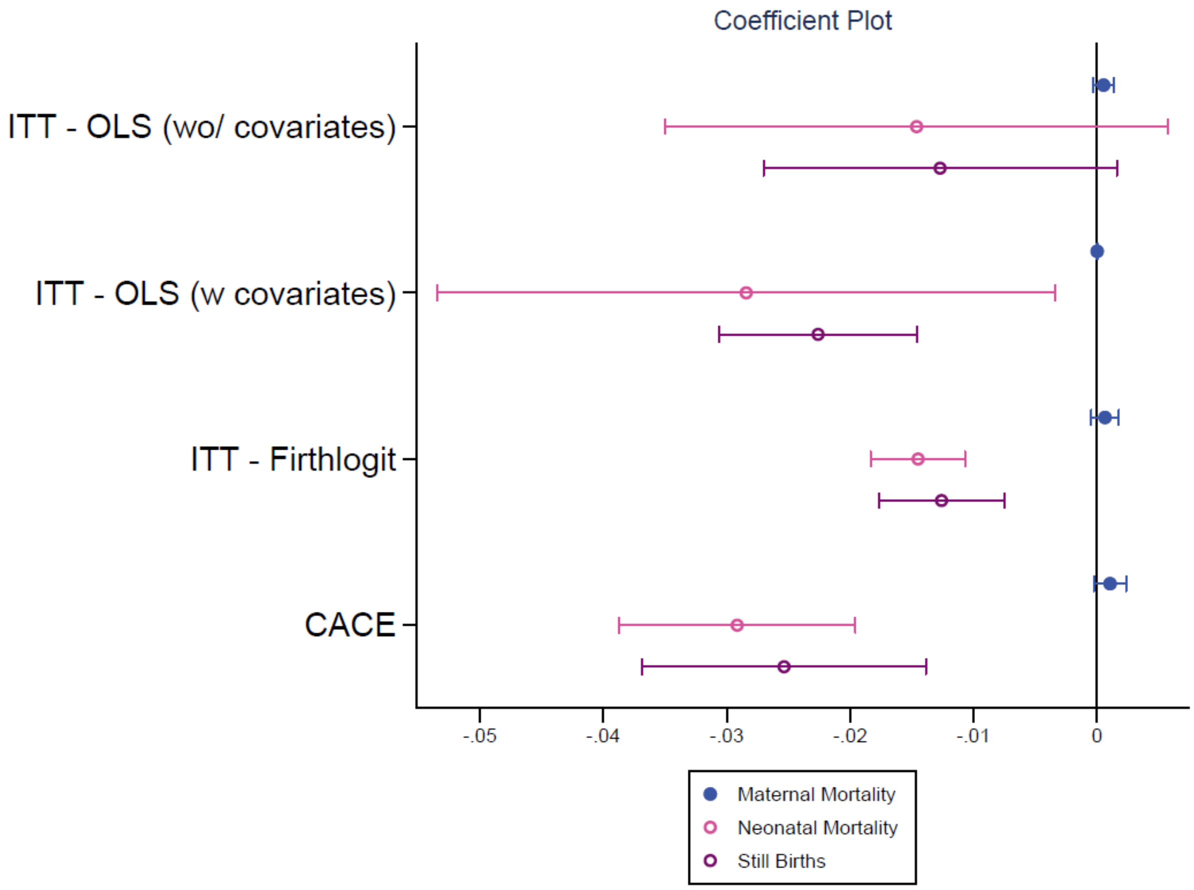

Note: Coefficient plots indicate 90% confidence intervals. eTable 5 provides the corresponding point estimates. Covariates included in row 2 refer to facility type, districts, urban-rural samples and CEmONC provision. The penalized maximum likelihood estimator is described in the appendix.[32]

© 2021 Kaplan LC et al. *JAMA Network Open*.

eFigure 2: Adapted SCC

Source: WHO (2015) and own adaptation.

World Health Organization

Mother's name____________ Age_____ Height/Weight__cm/____kg Insurance class__________ G
(No of pregnancies):__ P (No of deliveries): __ A (No of abortion):__ Facility name________ Date________

1 On Admission

Does mother need referral?
- ☐ No
- ☐ Yes, organized

Check your faciliy's criteria

Partograf started?
- ☐ No, will start when ≥ 4 cm
- ☐ Yes

Start plotting when cervix ≥4 cm, then cervix should dilate ≥1 cm/hr
- Every 30 min: plot HR, contractions, fetal HR
- Every 2 hrs: plot temperature
- Every 4 hrs: plot BP

Does mother need to start:

Antibiotics?
- ☐ No
- ☐ Yes, given

Date, time:

Ask for allergies before administration of any medication Give antibiotics to mother if any of:
- Mother's temperature ≥38°C
- History of foul-smelling vaginal discharge
- Rupture of membranes > 6 hrs

Magnesium sulfate (MgSO4) and antihypertensive treatment?
- ☐ No
- ☐ Yes, magnesium sulfate given
- ☐ Yes, antihypertensive given

Date, time:

Give magnesium sulfate to mother if any of:
- Diastolic BP ≥110 mmHg and 3+ proteinuria
- Diastolic BP ≥90 mmHg, 2+ proteinuria, and any: severe headache, visual disturbance, epigastric pain

Give antihypertensive medication to mother if systolic BP >160 mmHg
- Goal: keep BP <150/100 mmHg

☐ **Confirm supplies are available to clean hands and wear gloves for each vaginal exam.**

☐ **Encourage birth companion to be present at birth.**

☐ **Confirm that mother or companion will call for help during labor.**

Call help if any of:
- Bleeding
- Severe abdominal pain
- Severe headache or visual disturbance
- Unable or difficulty to urinate
- Urge to push
- Breathing difficulty
- Fever or chills
- Epigastric pain

This checklist is not intended to be comprehensive and should not replace the case notes or partograph. The checklist is based on the WHO 2015 pilot version, which was translated by Dr. Samuel J Oiam, MPH for WHO Country Office for Indonesia. As recommended by the WHO, the research team from University of Syiah Kuala and Göttingen University added modifications to fit local practices. This checklist is a pilot version for Aceh Province, Indonesia from 2016.

© WHO 2015

WHO Safe Childbirth Checklist **Completed by** ______________

WHO Safe Childbirth Checklist

World Health Organization

2 Just Before Pushing (Or Before Caesarean)

Does mother need to start:

Antibiotics?
- ☐ No
- ☐ No, already started (when?)
- ☐ Yes, given

Date, time:

Ask for allergies before administration of any medication. Give antibiotics to mother if any of:
- Mother's temperature ≥38°C
- History of foul-smelling vaginal discharge
- Rupture of membranes >6 hrs
- Caesarean section

Magnesium sulfate (MgSO4) and antihypertensive treatment?
- ☐ No
- ☐ No, already started (when?)
- ☐ Yes, given

Date, time:

Antihypertensive treatment?
- ☐ No
- ☐ No, already started (when?)
- ☐ Yes, given

Date, time:

Give magnesium sulfate to mother if any of:
- Diastolic BP ≥110 mmHg and 3+ proteinuria
- Diastolic BP ≥90 mmHg, 2+ proteinuria, and any: severe headache, visual disturbance, epigastric pain

Give antihypertensive medication to mother if systolic BP >160 mmHg
- Goal: keep BP <150/100 mmHg

Confirm essential supplies are at bedside and prepare for delivery:

For mother:
- ☐ Alcohol based handrub or soap and clean water
- ☐ APD
- ☐ Oxytocin 10 units in syringe
- ☐ Partus set

Prepare to care for mother immediately after birth:
Confirm single baby only (not multiple birth)
1. Give oxytocin within 1 minute after birth
2. Deliver placenta 1-15 minutes after birth
3. Massage uterus after placenta is delivered
4. Confirm uterus is contracted

For baby
- ☐ Clean towel
- ☐ Tie or cord clamp
- ☐ Sterile scissors/knife to cut cord
- ☐ Suction device
- ☐ Bag and mask
- ☐

Prepare to care for baby immediately after birth:
1. Dry baby, keep warm
2. If not breathing, stimulate and clear airway
3. If still not breathing:
- Clamp and cut cord
- Clean airway if necessary
- Ventilate with bag- and mask
- Shout for help

☐ **Assistant identified and ready to help at birth if needed.**

This checklist is not intended to be comprehensive and should not replace the case notes or partograph. The checklist is based on the WHO 2015 pilot version, which was translated by Dr. Samuel J Oiam, MPH for WHO Country Office for Indonesia. As recommended by the WHO, the research team from University of Syiah Kuala and Göttingen University added modifications to fit local practices. This checklist is a pilot version for Aceh Province, Indonesia from 2016.

© WHO 2015

WHO Safe Childbirth Checklist **Completed by** ______________

© 2021 Kaplan LC et al. *JAMA Network Open*.

WHO Safe Childbirth Checklist

World Health Organization

3 Soon After Birth (Within 1 Hour)

Is mother bleeding abnormally?

- ☐ No.
- ☐ Yes, shout for help.
- ☐ P: Yes, treat, but if cause cannot be treated, refer,

If bleeding abnormally:

- Massage uterus
- Consider more uterotonic
- Start IV fluids and keep mother warm
- Treat cause: uterine atony, retained placenta/fragments, vaginal tear, uterine rupture, blood coagulation
- If cause cannot be treated, refer based on your criteria

Does mother need to start:

Antibiotics?

- ☐ No
- ☐ No, already started (when?)
- ☐ Yes, given

Date, time:

Ask for allergies before administration of any medication

Give antibiotics to mother if placenta manually removed or if mother's temperature ≥38 °C and any of:

- Chills
- Foul-smelling vaginal discharge

If the mother has a third or fourth degree of perineal tear give antibiotics to prevent infection

Magnesium sulfate (MgSO4)?

- ☐ No
- ☐ No, already started (when?)
- ☐ Yes, given

Date, time:

Give magnesium sulfate to mother if any of:

- Diastolic BP ≥110 mmHg and 3+ proteinuria
- Diastolic BP ≥90 mmHg, 2+ proteinuria, and any: severe headache, visual disturbance, epigastric pain

Antihypertensive treatment?

- ☐ No
- ☐ No, already started (when?)
- ☐ Yes, given

Date, time:

Give antihypertensive medication to mother if systolic BP >160 mmHg

- Goal: keep BP <150/100 mmHg

Does baby need:

Referral?

- ☐ No
- ☐ Yes, organized

Check your facility's criteria.

Refer, if the following cannot be treated:

Antibiotics?

- ☐ No
- ☐ Yes, given
- ☐ Yes, but antibiotics cannot be given, refer based on your referral criteria

Date, time:

Give baby antibiotics if antibiotics given to mother for treatment of maternal infection during childbirth or if baby has any of: :

- Respiratory rate >60/min or <30/min
- Chest in-drawing, grunting, or convulsions
- Poor movement on stimulation
- Baby's temperature <35 °C (and not rising after warming) or baby's temperature ≥38 °C

Special care and monitoring needed?

- ☐ No
- ☐ Yes, organized
- ☐ Yes, treat, and refer based on your referral criteria, if no specialist present

Arrange special care / monitoring for baby if any:

- More than 1 month early
- Birth weight <2500 grams or >4000 grams
- Needs antibiotics
- Required resuscitation

Start breastfeeding and skin-to-skin contact (IMD) (if mother and baby are well).

- ☐ Yes, started immediately
- ☐ No, start later because mother or baby are not well

☐ **Inform mother / companion about danger signs and confirm thathelp will be called for if these signs present.**

This checklist is not intended to be comprehensive and should not replace the case notes or partograph. The checklist is based on the WHO 2015 pilot version, which was translated by Dr. Samuel J Oam, MPH for WHO Country Office for Indonesia. As recommended by the WHO, the research team from University of Syiah Kuala and Göttingen University added modifications to fit local practices. This checklist is a pilot version for Aceh Province, Indonesia from 2016.

© WHO 2015

WHO Safe Childbirth Checklist **Completed by** ______________________

Mother well and alive? Yes / No	Mother complications No / Yes, if any, mention:
Child well and alive? Yes / No	Child complications? No / Yes, if any, mention:

World Health Organization

4 Before Discharge

☐ **Confirm stay at facility for 24 hours after delivery.**

Does mother need to start antibiotics?

- ☐ No
- ☐ No, already started (when?)
- ☐ Yes, given and delay discharge.

Date, time:

Ask for allergies before administration of any medication Give antibiotics to mother if any of:

- Mother's temperature ≥38 °C
- Foul-smelling vaginal discharge

Is mother's blood pressure normal?

- ☐ Yes
- ☐ No, treat, delay discharge and check your referral criteria.

Give magnesium sulfate to mother if any of:

- Diastolic BP ≥110 mmHg and 3+ proteinuria
- Diastolic BP ≥90 mmHg, 2+ proteinuria, and any: severe headache, visual disturbance, epigastric pain

Give antihypertensive medication to mother if systolic BP >160 mmHg

- Goal: keep BP <150/100 mmHg

Is mother bleeding abnormally?

- ☐ No
- ☐
- ☐ Yes, treat, delay discharge and check your referral criteria

If pulse >110 beats per minute and blood pressure <90 mmHg

- Start IV and keep mother warm
- Treat cause (hypovolemic shock)

Does baby need to start antibiotics?

- ☐ No
- ☐ No, already given (when?)
- ☐ Yes, give antibiotics, delay discharge, give special care
- ☐ Yes, but antibiotics cannot be given → refer based on your referral criteria

Date, time:

Give antibiotics to baby if any of:

- Respiratory rate >60/min or <30/min
- Chest in-drawing, grunting, or convulsions
- Poor movement on stimulation
- Baby's temperature <35 °C (and not rising after warming) or baby's temperature ≥38 °C
- Stopped breastfeeding well
- Umbilicus redness extending to skin or draining pus

Is baby feeding well?

- ☐ No, establish good breastfeeding practices and delay discharge or refer for special treatment
- ☐ Yes

Discuss and offer family planning options to mother.

- ☐ Yes, done
- ☐ No, already done in antenatal care
- ☐ No, will be done later

☐ **Arrange follow-up for mother and baby**

☐ **(Inform mother / companion about danger signs) and confirm mother / companion will seek help if danger signs appear after discharge.**

Danger signs

***Mother** has any of:*

- Bleeding
- Severe abdominal pain
- Severe headache or visual disturbance
- Breathing difficulty
- Fever or chills
- Difficulty emptying bladder
- Epigastric pain

***Baby** has any of:*

- Fast/difficult breathing
- Fever
- Unusually cold
- Stops feeding well
- Less activity than normal
- Whole body becomes yellow
- (clean cord care) / cord care infection

This checklist is not intended to be comprehensive and should not replace the case notes or partograph. The checklist is based on the WHO 2015 pilot version, which was translated by Dr. Samuel J Oam, MPH for WHO Country Office for Indonesia. As recommended by the WHO, the research team from University of Syiah Kuala and Göttingen University added modifications to fit local practices. This checklist is a pilot version for Aceh Province, Indonesia from 2016.

© WHO 2015

WHO Safe Childbirth Checklist **Completed by** ______________________

© 2021 Kaplan LC et al. *JAMA Network Open*.

eTable 1: Minimal detectable effect sizes for applied essential practices (different % of take-up)

	sd	Baseline ICC	MDE (c = 0.2)	MDE (c = 0.5)	MDE (c = 0.9)
Practices PP1	1.575	0.120	5.026	2.011	1.117
Practices PP2	3.337	0.382	16.913	6.765	3.758
Practices PP3	1.873	0.205	7.307	2.923	1.624
Practices PP4	1.936	0.309	8.953	3.581	1.990
Practices All	6.691	0.278	29.585	11.834	6.574

SD: Standard deviation. ICC: Intra-cluster correlation. MDE: Minimal detectable effect. C:Take up rate. Assumptions: Sample size: 300, number of clusters: 15 providers, deviation of observations between treatment and control: 70:30. PP refers to Pause Points as depicted in the SCC in Figure 2.

© 2021 Kaplan LC et al. *JAMA Network Open*.

eTable 2: Minimal detectable effect sizes for health outcomes

	Mean - Full sample	MDE - 0.95 CI	MDE - 0.90 CI
Maternal Death	0.001	0.008	0.007
Stillbirth	0.012	0.044	0.039
Newborn Death	0.002	0.011	0.010
Complications of Mother	0.285	0.666	0.591
Complications of Newborn	0.289	1.136	1.008

Notes: Based on 50% uptake from compliance measure used.

© 2021 Kaplan LC et al. *JAMA Network Open*.

eTable 3: Mortality (individual births)

	Control		Treatment		OLS - ITT w.o. covariates		OLS - ITT w. covariates		Firth Logit		CACE		Benjamini-Hochberg Correction
	N	Mean Rate	N	Mean Rate	Diff.	95% CI	Diff.	95% CI	Diff.	95% CI	Diff.	95% CI	CACE Significant at 10% level
Maternal Mortality[#]	3,599	<0.0001	2,179	0.0005	0.0005	[-0.0004| 0.0013]	<0.0001	[-0.0002| 0.0001]	0.0005	[-0.0006| 0.0017]	0.0010	[-0.0005|0.0024]	No
Stillbirth[#]	3,599	0.0192	2,179	0.0073	-0.0118	[-0.0272| 0.0036]	-0.0224***	[-0.0318|- 0.0130]	-0.0117***	[-0.0175| -0.0060]	-0.0249***	[-0.0384| -0.0114]	Yes
Neonatal Mortality[#]	3,599	0.0147	2,179	0.0014	-0.0133	[-0.0352| 0.0085]	-0.0263*	[-0.0530| 0.0003]	-0.0133***	[-0.0176| -0.0090]	-0.0281***	[-0.0391| -0.0172]	Yes

Notes: Confidence intervals based on ordinary least squares (Intention To Treat) and two-stage-least-squares (Complier Average Causal Effect) estimations in brackets. p-val *<10%, **<5% and ***<1%. [#]Outcomes refer to individual probabilities and point estimates and 95% CI refer to marginal effects. Covariates are depicted in eTable 6.

For two facilities, we had to impute rates based on one observation month (March 2017), as data at the facility-level was not available for multiple months. Yearly birth volumes from the baseline data collection, however, strongly correspond to the monthly births in March 2017.

© 2021 Kaplan LC et al. *JAMA Network Open*.

eTable 4: Mortality and morbidity (facility level rates)

<table>
<tr><th></th><th colspan="2">Control</th><th colspan="2">Treatment</th><th colspan="4">ITT</th><th colspan="4">CACE</th><th>Benjamini-Hochberg Correction</th></tr>
<tr><th></th><th>N (Facilities)</th><th>Mean Rate</th><th>N (Facilities)</th><th>Mean Rate</th><th>Diff. w.o. covariates</th><th>95% CI</th><th>Diff. w. covariates</th><th>95% CI</th><th>Diff. w.o. covariates~</th><th>95% CI</th><th>Diff. w. covaraites~~</th><th>95% CI</th><th>CACE Significant at 10% level</th></tr>
<tr><td>Maternal Mortality</td><td>15</td><td>0</td><td>15</td><td>0.0002</td><td>0.0002</td><td>[0.0 | .001]</td><td>0</td><td>[-0.001 | 0.0]</td><td>0.0004</td><td>[0.0 | .001]</td><td>0</td><td>[-0.001 | 0.0]</td><td>No</td></tr>
<tr><td>Stillbirth</td><td>15</td><td>0.0213</td><td>15</td><td>0.0154</td><td>-0.0059</td><td>[-0.0032 | 0.021]</td><td>-0.0023</td><td>[-0.034 | 0.029]</td><td>-0.0121</td><td>[-0.063 | 0.039]</td><td>-0.0052</td><td>[-0.063 | 0.053]</td><td>No</td></tr>
<tr><td>Neonatal Mortality</td><td>15</td><td>0.0152</td><td>15</td><td>0.0017</td><td>-0.0135</td><td>[-0.0310 | 0.0040]</td><td>-0.0139</td><td>[-0.034 | 0.006]</td><td>-0.0278</td><td>[-0.062 | 0.007]</td><td>-0.0322*</td><td>[-0.068 | 0.004]</td><td>No</td></tr>
<tr><td>Maternal Complications</td><td>15</td><td>0.1564</td><td>14^</td><td>0.3113</td><td>0.1549</td><td>[-0.216 | 0.526]</td><td>0.2723</td><td>[-0.181 | 0.726]</td><td>0.3081</td><td>[-0.356 | 0.972]</td><td>0.5903</td><td>[-0.141 | 1.322]</td><td>No</td></tr>
<tr><td>Neonatal Complications</td><td>15</td><td>0.1671</td><td>14^</td><td>0.1062</td><td>-0.061</td><td>[-0.257 | 0.135]</td><td>-0.0359</td><td>[-0.223 | 0.151]</td><td>-0.1213</td><td>[-0.489 | 0.246]</td><td>-0.0779</td><td>[-0.394 | 0.238]</td><td>No</td></tr>
</table>

Notes: Confidence intervals based on ordinary least squares (Intention To Treat) and two-stage-least-squares (Complier Average Causal Effect) estimations in brackets. p-val *<10%, **<5% and ***<1%. The set of covariates include District, Urban-Rural, CEmONC and Facility Type.

^ One facility did not report any outcomes with regard to complications in the endline data collection.

For two facilities, we had to impute rates based on one observation month (March 2017), as data at the facility-level was not available for multiple months. Yearly birth volumes from the baseline data collection, however, strongly correspond to the monthly births in March 2017.

© 2021 Kaplan LC et al. *JAMA Network Open*.

eTable 5: Mortality and morbidity (subsample of facilities where observations were conducted)

	Control		Treatment		ITT				CACE				Benjamini-Hochberg Correction
	N (Facilities)	Mean Rate	N (Facilities)	Mean Rate	Diff. w.o. covariates	95% CI	Diff. w. covariates	95% CI	Diff. w.o. covariates[~]	95% CI	Diff. w. covaraites[~~]	95% CI	CACE Significant at 10% level
Maternal Mortality[#]	7	0	9	0.000003	0.000003	[-0.000| 0.001]	0	[-0.001| 0.001]	0.000006	[-0.001| 0.002]	0	[-0.001| 0.001]	No
Stillbirth[##]	7	0.0168	9	0.0191	0.0022	[-0.033| 0.038]	0.0013	[-0.035| 0.060]	0.004	[-0.051| 0.059]	0.0253	[-0.029| 0.080]	No
Neonatal Mortality[##]	7	0.0254	9	0.013	-0.0241	[-0.056| 0.008]	-0.0252	[-0.065| 0.0014]	-0.0438*	[-0.095| 0.007]	-0.0492**	[-0.096| -0.002]	Yes
Maternal Complications[##]	7	0.1499	9	0.4304	0.2805	[-0.420| 0.981]	0.777	[-0.210| 1.764]	0.477	[-0.529| 1.483]	1.3066**	[0.296| 2.317]	Yes
Neonatal Complications[##]	7	0.329	9	0.1259	-0.2028	[-0.567| 0.161]	-0.0773	[-0.546| 0.392]	-0.345	[-0.895| 0.206]	-0.13	[-0.582| 0.322]	No

Notes: Confidence intervals based on ordinary least squares (Intention To Treat) and two-stage-least-squares (Complier Average Causal Effect) estimations in brackets. p-val *<10%, **<5% and ***<1%. The set of covariates include District, Urban-Rural, CEmONC and Facility Type.

[^] One facility did not report any outcomes with regard to complications in the endline data collection. Rates refer to [#]x/100,000, [##]x/1,000.

For two facilities, we had to impute rates based on one observation month (March 2017), as data at the facility-level was not available for multiple months. Yearly birth volumes from the baseline data collection, however, strongly correspond to the monthly births in March 2017.

© 2021 Kaplan LC et al. *JAMA Network Open*.

eTable 6: Point estimates for covariates

	Neonatal Mortality	Stillbirth	Maternal Mortality
**Treatment**	-0.0263*<br>[-0.0541; 0.0014]	-0.0224***<br>[-0.0322|-0.0126]	-0.0000<br>[-0.0002|0.0001]
**Public Hospital**	0.0680*<br>[-0.0076|0.1437]	0.0286*<br>[-0.0034|0.0606]	0.0014<br>[-0.0003|0.0032]
**Private Hospital**	0.0194<br>[-0.0215|0.0602]	0.0096<br>[-0.0208|0.0400]	0.0016<br>[-0.0003|0.0035
**Private Midwife Clinic**	0.0220<br>[-0.0205|0.0645]	-0.0017<br>[-0.0311|0.0277]	0.0000<br>[-0.0001|0.0002]
**Bireuen District**	0.0264<br>[-0.0060|0.0587]	0.0213***<br>[0.0150|0.0276]	-0.0000<br>[-0.0002|0.0001]
**Aceh Besar District**	-0.0369**<br>[-0.0692|-0.0046]	-0.0101<br>[-0.0281|0.0079]	-0.0009<br>[-0.0022|0.0004]
**Rural**	0.0340<br>[-0.0076|0.0756]	0.0040<br>[-0.0297|0.0377]	0.0007<br>[-0.0004|0.0018]
**CEmONC Services**	-0.0271*<br>[-0.0570|0.0029]	-0.0271***<br>[-0.0398|-0.0143]	-0.0015<br>[-0.0034|0.0003]
**N**	5778	5778	5778

© 2021 Kaplan LC et al. *JAMA Network Open*.

eTable 7: Case Numbers per Facility

FacilityID	Births	Maternal Deaths	Neonatal Deaths	Stillbirhts	Mat. Compl.	Neon. Compl.	Part of Observations
**1**	329	0	39	17	60	406	Yes
**2**	696	0	6	0	0	0	Yes
**3**	965	0	5	29	79	368	Yes
**4**	26	0	0	1	1	1	No
**5**	20	0	1	0	2	9	Yes
**7**	3	0	0	0	1	0	No
**8**	5	0	0	0	0	0	No
**9**	0	0	0	0	0	0	No
**10**	20	0	1	1	0	1	No
**11**	14	0	0	0	10	3	No
**12**	18	0	0	0	1	3	No
**13**	7	0	0	1	6	1	No
**14**	8	0	0	1	20	0	Yes
**15**	168	0	0	1	4	15	Yes
**16**	14	0	0	0	1	0	No
**17**	65	0	0	0	2	3	No
**18**	167	0	0	0	75	5	Yes
**19**	1	0	0	0	0	0	No
**20**	50	0	0	1	8	2	Yes
**21**	73	0	1	0	4	1	No
**22**	21	0	0	0	5	0	Yes
**23**	228	0	0	2	8	3	No
**25**	94	0	0	2	2	0	No
**28**	0	0	0	0	0	0	No
**29**	202	0	0	2	8	17	Yes
**30**	168	0	0	2	21	34	Yes
**32**	4	0	0	0	0	0	No
**33**	325	1	0	5	36	42	Yes
**34**	1584	0	7	10	58	129	Yes

© 2021 Kaplan LC et al. *JAMA Network Open*.

**35**	302	0	1	4	106	167	Yes
**37**	81	0	0	0	7	2	Yes
**38**	120	0	0	0	1	1	Yes

© 2021 Kaplan LC et al. *JAMA Network Open*.

eTable 8: Depiction of Safe Childbirth Checklist and Research Team Exposure among Treatment and Control Group

Treatment	*Control*
SCC exposure:<br><br>- 1 x Checklist Introduction event (2 hours): Presentation| checklist explanation| role play (no training)| selection of (non-remunerated) checklist quality coordinators<br>- 11 x Monitoring visits over six months (2 hours each): SCC provision & collection| interviews with providers| feedback on previous performance| opportunity to ask questions<br>- 2 x Meetings of facility-based checklist quality coordinators (2 hours each) three- and six-months post introduction: Focus group discussions to exchange best practice<br>- Checklist provision and provision of danger sign sheets<br>Exposure to research team:<br><br>- Information event for facility leadership to present study design<br>- Observations (24 hours over six days in larger facilities and one month on call in smaller facilities)<br>- Survey on provider characteristics and perceptions (30-40 minutes)	SCC exposure:<br><br>- None<br>Exposure to research team:<br><br>- Information event for facility leadership to present study design<br>- Observations (24 hours over six days in larger facilities and one month on call in smaller facilities)<br>- Survey on provider characteristics and perceptions (30-40 minutes)

eTable 9: Background information – Complications

Maternal complications	*Neonatal complications*
Pre-eclampsia| eclampsia| rupture uterus| postpartum hemorrhage| wound infections| obstructed or prolonged labor| sepsis| bad or foul-smelling discharge| antepartum hemorrhage| others	Birth trauma| Asphyxia| Hypothermia| Respiratory distress syndrome| neonatal sepsis| prematurity| small for gestational age| low birth weight (<2500g)| umbilical cord infection| fever| jaundice| others

© 2021 Kaplan LC et al. *JAMA Network Open*.
