# Supplementary material for: Effects of the World Health Organization Safe Childbirth Checklist on Quality of Care and Birth Outcomes in Aceh, Indonesia: A Cluster-Randomized Clinical Trial
Source: JAMA Netw Open. 2021 Dec 3;4(12):e2137168. doi: 10.1001/jamanetworkopen.2021.37168 (PMC8642783; doi:10.1001/jamanetworkopen.2021.37168)
Supplement: Supplement 3. — Data Sharing Statement [file jamanetwopen-e2137168-s003.pdf]

Data Sharing Statement

Kaplan. Effects of the World Health Organization Safe Childbirth Checklist on Quality of Care and Birth Outcomes in Aceh, Indonesia. *JAMA Netw Open*. Published December 03, 2021.
doi:10.1001/jamanetworkopen.2021.37168

Data
Data available: Yes
Data types: Deidentified participant data, Data dictionary
How to access data: Once the paper gets published, we would also make our data available.
When available: With publication

Supporting Documents
Document types: None

Additional Information
Who can access the data: Researchers whose proposed use of the data has been approved
Types of analyses: Replication
Mechanisms of data availability: After approval of a proposal
Any additional restrictions: Data would be deidentified.
